# Enhanced detection and molecular modeling of adaptive mutations in SARS-CoV-2 coding and non-coding regions using the c/µ test

**DOI:** 10.1093/ve/veae089

**Published:** 2024-11-06

**Authors:** Nicholas J Paradis, Chun Wu

**Affiliations:** Department of Chemistry and Biochemistry, Rowan University, 201 Mullica Hill Rd., Glassboro, NJ 08028, United States; Department of Chemistry and Biochemistry, Rowan University, 201 Mullica Hill Rd., Glassboro, NJ 08028, United States; Department of Biological & Biomedical Sciences, Rowan University, 201 Mullica Hill Rd., Glassboro, NJ 08028, United States

**Keywords:** SARS-COV-2, c/µ, Ks/µ, Ka/Ks, nonsynonymous, synonymous, TR, UTR

## Abstract

Accurately identifying mutations under beneficial selection in viral genomes is crucial for understanding their molecular evolution and pathogenicity. Traditional methods like the Ka/Ks test, which assesses non-synonymous (Ka) versus synonymous (Ks) substitution rates, assume that synonymous substitutions at synonymous sites are neutral and thus is equal to the mutation rate (µ). Yet, evidence suggests that synonymous sites in translated regions (TRs) and untranslated regions (UTRs) can be under strong beneficial selection (Ks > µ) and strongly conserved (Ks ≈ 0), leading to false predictions of adaptive mutations from codon-by-codon Ka/Ks analysis. Our previous work used a relative substitution rate test (c/µ, c: substitution rate in UTR/TR, and µ: mutation rate) to identify adaptive mutations in SARS-CoV-2 genome without the neutrality assumption of the synonymous sites. This study refines the c/µ test by optimizing µ value, leading to a smaller set of nucleotide and amino acid sites under beneficial selection in both UTR (11 sites with c/µ > 3) and TR (69 nonsynonymous sites: c/µ > 3 and Ka/Ks > 2.5; 107 synonymous sites: Ks/µ > 3). Encouragingly, the top two mutations in UTR and 70% of the top nonsynonymous mutations in TR had reported or predicted effects in the literature. Molecular modeling of top adaptive mutations for some critical proteins (S, NSP11, and NSP5) was carried out to elucidate the possible molecular mechanism of their adaptivity.

## Introduction

Correctly identifying the top mutations under strong beneficial selection for individual nucleotide (NT) and amino acid (AA) sites within viral genomes is critical for understanding their role in molecular evolution. Such mutations reportedly enhance enzyme activity (Cassari et al. 2023), viral replication (Lanahan et al. 2021), and promote drug/vaccine/antibody resistance (Harvey et al. 2021). For SARS-CoV-2, a single-stranded positive-sense RNA virus emerging in December 2019, point mutations within several proteins (e.g. S, N, NSP5, NSP11) have been associated with their fitness change, from drug/vaccine resistance (Chand et al. 2020, Pachetti et al. 2020, Grabowski et al. 2021, Ullrich et al. 2022, Iketani et al. 2023, Ip et al. 2023, Jiang et al. 2023, Kawashima et al. 2023), increased host infectivity (Walls et al. 2020, Liu et al. 2022b), easier spreading to children (Waghmare and Hijano 2023), lower mortality, and higher residence time (Hedberg et al. 2024). Diagnostic tools such as conventional Ka/Ks test (Ka: nonsynonymous substitution rate, Ks: synonymous substitution rate) is one of the most promising methods to quantify the top adaptive nonsynonymous (i.e. amino acid change) mutations within proteins, but Ka/Ks has critical limitations. The Ka/Ks test quantifies the fitness change in only the translated region (TR) of a genome (neutral selection: Ka/Ks = 1; beneficial selection: Ka/Ks > 1; purifying selection: Ka/Ks < 1), under a critical assumption that synonymous mutations are neutral from a protein-centric viewpoint (Kimura 1977). The assumption implies that the Ka/Ks test is not applicable to both synonymous sites in TR and the non-coding NT sites in untranslated region (UTR), both assumed to be selectively neutral from the protein-centric viewpoint. On the contrary, mounting evidence supporting non-neutral nucleic acid molecular evolution points to synonymous sites in TR and regulatory sites in UTR being under strong natural selection (Ks>>µ or Ks<<µ), supporting their critical roles in the transcription and translation of DNA and RNA (Clarke 1970, Minchin and Lodge 2019). Thereby, developing a robust diagnostic tool to quantify the top mutations in UTR and TR under strong beneficial selection within a viral genome can enhance the focus on mutations to characterize experimentally.

Our recently proposed replication-selection model provides a simple approach to quantify the different selection pressure types on any NT site in the TR and UTR of a genome using empirical sequence data (Wu et al. 2023): Our model assumes the mutation and selection steps are independent from each other (Teufel et al. 2018). From this model, the substitution rate (c) in the population can be understood as the product of the mutation rate (µ) and a fixation ratio (c/µ, i.e. the relative rate ratio test). The c/µ test can detect the fitness change due to mutations in both TR and UTR and while c/µ is similar to the Ka/Ks ratio test, it does not require the critical assumption of synonymous mutations to be under neutral selection (neutral selection: c/µ = 1; beneficial selection: c/µ > 1; purifying selection: c/µ < 1). Because the total substitution rate (c) in TR is the weighted average of the nonsynonymous rate (Ka) and the synonymous rate (Ks) (i.e. c = Ps*Ks + Pa*Ka, Ps and Pa: proportions of synonymous and nonsynonymous sites in TR), a general equation linking c/µ with weighted Ks/µ and Ka/µ can be derived: c/µ = Ps*(Ks/μ) + Pa*(Ka/μ). This general equation demonstrates that Ka/Ks infers the same mutation type as c/µ does only if synonymous mutations are neutral (i.e. Ks/µ = 1), otherwise the overall fitness change (c/µ) is determined from both synonymous mutations (Ks/µ) and nonsynonymous mutations (Ka/µ). Using SARS-CoV-2 as a case study, the c/µ for each NT site in the viral genome (29,903 NTs) was obtained from its first 19 months of genomic data (three independent genomic datasets totaling 11,198 genome sequences from NextStrain; Hadfield et al. 2018) to quantify the fitness change due to mutations for the very first time. It was observed that most sites in TR and UTR are under non-neutral selection, with most sites being conserved and few sites showing high c/µ values indicating adaptive selection.

When compared to c/μ, the Ka/Ks test quantifies the fitness change resulting from nonsynonymous mutations changing the protein sequence (Yang and Bielawski 2000) and does not include the fitness change resulting from synonymous mutations changing the nucleic acid sequence only (Ks/µ). By not including Ks/µ, the fitness change that can impact transcription and translation processes, mRNA splicing, and mRNA thermostability cannot be observed (Clarke 1970); in contrast, Ks/µ can be easily determined from the c/µ test, thus the top synonymous NT mutations under beneficial selection can be detected, thus their impact on nucleic acid processes can be inferred. Furthermore, the enhanced robustness of c/µ compared to Ka/Ks is due to the mutation rate µ being a well-defined non-zero constant; in contrast, Ka/Ks analysis is not well-defined for segments with very low Ks (Ks ≈ 0), very short segments and when performing site-by-site analysis (Pond and Frost 2005). Specifically, while Ks can vary between sites, µ is a constant, thus performing site-by-site analyses using c/µ and Ks/µ will not invoke technical errors similarly to Ka/Ks. From this, c/µ can provide a more comprehensive analysis of fitness change across both synonymous and nonsynonymous coding sites and all non-coding sites within the genomic space.

In this report, we extended our c/μ test to SARS-CoV-2 genomic data from our previous study, performing a site-by-site analysis to identify NT and AA sites under strong beneficial selection in both UTR and TR within 10 coding genes in TR (25 proteins), 11 UTRs and 9 Transcriptional Regulatory Sequences (TRSs) by using a numerical cutoff for UTR (c/µ > 3), non-synonymous sites in TR (c/μ > 3 and Ka/Ks > 2.5), and synonymous sites in TR (c/μ > 3 and Ks/µ > 3). We refined the c/µ test by optimizing µ value, leading to a smaller set of nucleotide and amino acid sites under beneficial selection. We compared the capabilities of c/µ + Ka/Ks versus Ka/Ks in identifying the top nonsynonymous mutations, opting for the combined c/µ + Ka/Ks approach as c/µ reduces the number of technical false beneficials (i.e. Ks = 0) that are encountered using Ka/Ks. We confirmed the fitness increase of the top nonsynonymous mutations in TR and top mutations in UTR were validated by the activity data from the literature, supporting that our c/µ test is more robust than the Ka/Ks test to detect molecular adaption. The assessment of Ks/µ for each site in TR was also performed to demonstrate the non-neutrality of many synonymous sites in TR. Lastly, to elucidate the molecular mechanism of top nonsynonymous mutations in critical proteins, we performed protein–protein and small molecule molecular docking on wild-type and top mutant homology models of S, NSP11, and NSP5 proteins. The observed changes in binding free energy between the wild-type and mutant structures will be inferred in the discussion section.

## Methods

### Computational workflow for identifying the top adaptive mutations in SARS-CoV-2 UTR and TR

**Figure 1.**
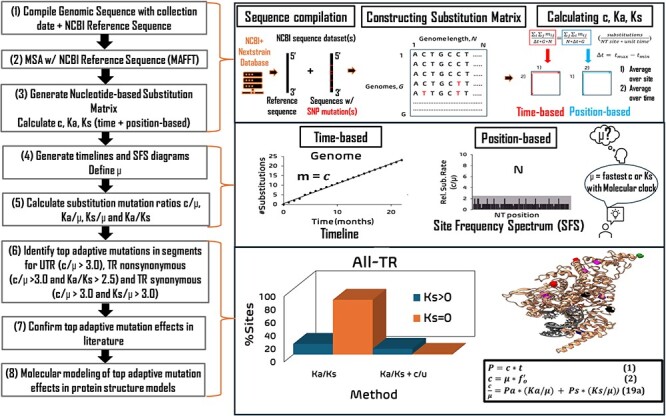
Computational workflow to identify and validate the top adaptive mutations under strong positive selection for the SARS-CoV-2 genomic datasets in this study.

The computational workflow for identifying the top adaptive mutations in SARS-CoV-2 UTR and TR is shown in Fig. 1. Absolute rate values (c, Ka, Ks) and relative rate values (c/μ, Ka/μ, and Ks/μ) were calculated for each NT and AA site in the SARS-CoV-2 genome (29,903 NTs), TR and UTR and each coding segment in TR, and each non-coding UTR and TRS (where applicable) using the genomic sequence data (11,198 genome sequences) and methods described in our previous study (Wu et al. 2023). The parameter µ is set to be the substitution rate of Orf1ab 5ʹUTR which is the highest one over all segments. Multiple sequence alignment was performed using the default MAFFT protocol, using the Wuhan-Hu-1 SARS-CoV-2 isolate as the reference sequence (Katoh et al. 2019). The nonsynonymous substitutions (Ka), synonymous substitutions (Ks), and Ka/Ks ratio values were calculated across each AA codon and across each coding gene segment for the three datasets using the Nei–Gojobori (NG) (Nei and Gojobori 1986, Nei and Jin 1989) method (JC69 model). NG was chosen, because our previous paper (Wu et al. 2023) have demonstrated that the NG results are consistent with that by Li–Wu–Luo (LWL) (Li et al. 1985) method, Pamilo–Bianchi–Li (PBL) (Pamilo and Bianchi 1993) method, and maximum likelihood (ML) (Goldman and Yang 1994) method in processing these SARS-CoV-2 datasets. To illustrate the sites which cannot be defined by Ka/Ks (i.e. when Ks = 0 and/or Ka = Ks = 0), the difference between Ka and Ks was also calculated for each site in TR (Ka − Ks).

The workflow proceeds in the following step-wise manner. (1) Download and compile three SARS-CoV-2 genomic datasets with explicit collection date (December 2019 to June 2021) from the NextStrain database; download the NCBI reference sequence (Wuhan-Hu-1). (2) Multiple sequence alignment (MSA) of the three SARS-CoV-2 genomic datasets against the reference sequence in chronological order (MAFFT). (3) Generate the nucleotide-based substitution matrix for the three SARS-CoV-2 genomic datasets (including segments in UTR and TR); calculate the total substitution rate for all nucleotide sites in TR and UTR (c), nonsynonymous substitution rate for all nonsynonymous sites in TR (Ka) and synonymous substitution rate for all synonymous sites in TR (Ks) in time-based and position-based approaches. (4) Generate timelines (time-based method, data not shown in this study) and site-frequency spectra (SFS) diagrams for the genome and each segment. Approximate the fundamental genomic mutation rate (µ) from the highest c, Ka, or Ks value for the segment exhibiting molecular clock (correlation coefficient, *R*^2^ > 0.6000). (5) Calculate the substitution-mutation ratios c/µ, Ka/µ and Ks/µ. Calculate Ka/Ks (Nei–Gojobori). (6) From SFS and cutoff values, identify the top adaptive mutation sites under strong beneficial selection in UTR, nonsynonymous sites in TR and synonymous sites in TR. (7) Confirm the mutation effects of the top adaptive mutations in the reported literature. (8) Perform computational modeling of the top mutation effects on protein function and biological processes (e.g. drug binding affinity).

### Identifying top NT/AA sites under strong beneficial selection in UTR and TR

The NT sites in UTR were sorted by position-based c/µ and top NT sites under strong beneficial selection were identified using the cutoff (c/μ > 3). The AA sites in All-TR were sorted by position-based c/µ; the top nonsynonymous AA sites under strong beneficial selection (c/μ > 3 and Ka/Ks > 2.5) and the top synonymous AA sites under strong beneficial selection (Ks/µ > 3) were sorted by position-based c/µ + Ka/Ks and by position-based Ks/µ, respectively.

### Prediction of RNA secondary structures

The Wuhan-Hu-1 reference DNA sequence of each SARS-CoV-2 segment in TR was submitted to the RNAFold webserver (Kerpedjiev et al. 2015) to predict their secondary RNA structures and to map the top synonymous mutations under strong beneficial selection.

### Protein homology modeling and molecular docking

Full-length homology models of wild-type SARS-CoV-2 proteins were obtained from I-TASSER (Yang et al. 2015) or the Protein Data Bank if provided. For the molecular docking protocol, the crystal structures of S (PDB ID: 6VSB) (Wrapp et al. 2020), S trimer-JMB2002 Fab complex (PDB ID: 7WPD) (Yin et al. 2022a), S RBD-hACE2 complex (PDB ID: 8ASY) (Huo et al. 2023), NSP5-nirmatrelvir complex (PDB ID: 8DZ2) (Noske et al. 2023), and NSP11-RNA-Remdesivir triphosphate-Mg^2+^ complex (PDB ID: 7BV2) (Yin et al. 2020) were obtained from the Protein Data Bank. The following protein backbones of crystal structures and homology models were aligned together (6VSB-8ASY, 6VSB-7WPD, NSP5 homology model-8DZ2, NSP11 homology model-7BV2), and the protein and/or non-protein components were then merged together using Maestro 2022-3. Specifically for S, the hACE2 (8ASY) and Fab (7WPD) were merged with the chain A monomer of S (6VSB). For NSP5, nirmatrelvir (8DZ2) was merged with the NSP homology model. For NSP11, remdesivir triphosphate, viral RNA and Mg^2+^ metal cofactors (7BV2) were merged with the NSP11 homology model. Maestro’s Protein Preparation wizard with a three-step protocol (pKa calculation → hydrogen optimization → restrained heavy atom geometry minimization) was used to prepare each *holo* structure prior to molecular docking. The mutant homology models were generated by introducing several top nonsynonymous mutations into each *holo* structure using Maestro. Molecular docking of the JMB2002 Fab antibody and the hACE2 receptor was performed on wild-type and mutant S protein structures using the mCSM-PPI and mCSM-AB2 webservers (Pires et al. 2014; Myung et al. 2020). For NSP5 and NSP11, several FDA-approved small molecule drug compounds were docked to their respective wild-type and top mutant protein homology structures using the Glide program of the Schrödinger 2022-3 software package (Friesner et al. 2006). The absolute binding free energy (ΔG_bind_) and relative binding free energy (ΔΔG_bind_) values were calculated for each ligand against the wild-type and top mutant structures where applicable.

### Supplementary data

Two data files will be supplied for the following information: (i) c, c/µ and the top mutations for each NT site of UTR. (ii) c, Ka, Ks, c/µ, Ka/Ks, Ka-Ks, Ka/µ, Ks/µ values and the top mutations for each AA site for each coding gene and the top nonsynonymous mutations identified using c/µ, Ka/Ks, c/µ + Ka/Ks, and Ks/µ. This information is sorted by decreasing c/µ and the top mutations are highlighted for ease-of-viewing.

## Results

The top mutations under strong beneficial selection were identified in both UTR and TR using our c/µ, combined c/μ + Ka/Ks and Ks/μ approaches where applicable. A methodological comparison between top mutant identification reported in our previous paper (Wu et al. 2023) and in this study is explained. Technical issues in using Ka/Ks alone to identify the top mutations were also highlighted. The inferred fitness change from sites identified via c/μ + Ka/Ks and Ks/μ approaches were compared with their reported mutation effects in the current literature. Key literature pieces of mutation effects in some UTRs and critical proteins (S, N, NSP11, and NSP5) were mainly discussed. Lastly, molecular docking analysis generally confirmed a beneficial mutation effect on S, NSP11, and NSP5 viral entry and drug resistance mechanisms.

### Refinement on µ reduces false positive predictions on top adaptive mutations

The top mutations identified using our combined c/µ + Ka/Ks approach was performed in our previous paper (Wu et al. 2023) for the UTR (54 NT sites) and TR (247 AA sites). In this study, the identified mutation count in UTR (3 NT sites) and in TR (69 nonsynonymous AA sites and 107 synonymous AA sites) (Supplementary Table S1) are attributed to changing the fundamental genomic mutation rate (µ), which we assume to be constant throughout all genomic space in our dataset. In the first paper, the position-based genomic substitution rate was defined as µ ($\mu = {\ }$GSR = $\frac{{0.0902\% {\ }substitutions}}{{NT{\ }site*{\ }19{\ }months}}$). In this study, we set µ to be the fastest position-based substitution rate, the Orf1ab 5ʹUTR substitution rate ($\frac{{0.4762\% {\ }substitutions}}{{NT{\ }site*19{\ }months}}$) to remove the violation of any segment exhibiting a fraction of nearly-neutral mutation sites as greater than 1 ($\frac{c}{\mu } = f_0^{\prime}= 1$) as we discuss in a separate study (under review). Thus, c/µ, Ka/µ and Ks/µ for each NT and codon site in each TR and UTR were calculated using the Orf1ab 5ʹUTR substitution rate as µ. As µ increases, c/µ, Ka/µ and Ks/µ values also decrease across each NT, codon, and segment, explaining the decreased top mutation count in the UTR (54 to 4 mutations) and nonsynonymous sites in TR (247 to 69 mutations) between our first paper and this study. Additionally, Ks/μ was included to identify the top 107 synonymous mutations in TR in this study only. Lastly, we did not exhaustively consult the known literature to infer the fitness change for all identified top mutations in UTR or TR; in this study, our literature search revealed 67% of the top mutations in UTR and ∼70% of nonsynonymous mutations in TR reported beneficial SARS-CoV-2 fitness changes.

### C/µ + Ka/Ks reduces technical false beneficial sites compared to Ka/Ks alone

**Figure 2.**
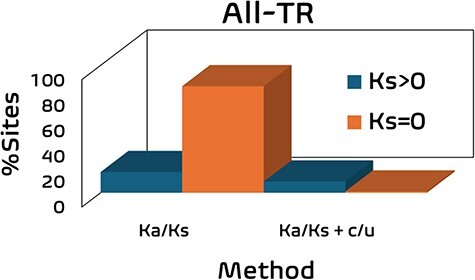
Graphical representation of the top nonsynonymous mutations under strong beneficial selection identified using Ka/Ks alone and combined c/µ + Ka/Ks methods in Table 1 and the number of mutations exhibiting valid Ka/Ks values (Ks > 0) and invalid Ka/Ks values (Ks = 0).

To demonstrate the improved accuracy combining c/µ + Ka/Ks methods in identifying the top nonsynonymous mutations, we compared this capability between c/µ alone, Ka/Ks alone, and c/µ + Ka/Ks. Moreover, we highlight the technical false beneficial sites detected using Ka/Ks (i.e. codon sites exhibiting Ka > 0 and Ks = 0, Ka/Ks = Undefined), suggesting these sites are under extreme beneficial selection. While conserved sites also provide the same undefined result (Ka = 0 and Ks = 0, Ka/Ks = Undefined), these sites are not under beneficial selection and were thus removed from this analysis. The results are summarized in Table 1 and Fig. 2.

**Table 1.** The number of top nonsynonymous mutations in TR identified using Ka/Ks alone, c/µ alone and combined c/µ + Ka/Ks methods within the SARS-CoV-2 datasets

c/µ^a^	Ka/Ks^a^^,^^b^	c/µ + Ka/Ks^a^^,^^b^
Total: 90	Total: 2972 Ks > 0: 485 (16.3%) Ks = 0: 2487 (83.7%)	Total: 69 Ks > 0: 43 (43/485: 8.9%) Ks = 0: 26 (26/2487: 1.0%)

a Top mutations were identified using the criteria for nonsynonymous sites in TR (c/µ > 3 and Ka/Ks > 2.5).

b Ka/Ks method: Nei–Gojobori (NG). For Ka/Ks, the identified mutation count in TR is further decomposed between with valid Ka/Ks values (Ks > 0) and invalid Ka/Ks values (Ks = 0, Ka/Ks = Undefined).

Using Ka/Ks alone, 2972 top nonsynonymous sites were detected; 485 sites (16.3%) exhibited valid Ka/Ks values and 2487 sites (83.7%) exhibited invalid Ka/Ks values. Using c/µ alone, 90 top sites were identified containing both valid and invalid Ka/Ks values. Using c/µ + Ka/Ks, 69 top nonsynonymous sites were identified; 43 sites exhibited valid Ka/Ks and 26 sites exhibited invalid Ka/Ks values. Compared to using Ka/Ks alone, c/µ + Ka/Ks reduced the number of valid Ka/Ks values by 0.5-fold and reduced the number of invalid Ka/Ks values by 83-fold (Table 1). Therefore, combining c/µ and Ka/Ks methods significantly reduced the Ka/Ks technical error in the codon-by-codon analysis. A caveat to utilizing c/µ alone is that this method infers selection type at both synonymous and nonsynonymous sites; using c/µ identified 21 sites where Ka/Ks < 2.5 due to increasing Ks (e.g. Ks/µ 4.5-610.8), indicating these synonymous sites are under strong beneficial selection. Thus, Ka/Ks is required with c/µ to obtain only nonsynonymous sites. From here, we will focus on the top mutations identified in UTR and TR.

### Top 11 mutations in Orf1ab 5ʹUTR and Orf10 3ʹUTR confirmed by c/µ test and literature mutation effects

c/µ was calculated for each NT site in each UTR and TRS (Supplementary Figs S1–S3). The c/µ distributions within Orf1ab 5ʹUTR and Orf10 3ʹUTR exhibits multiple sites likely strong beneficial selection (Supplementary Fig. S1), whereas the remaining UTRs and TRSs were highly conserved (Supplementary Figs S2–S3), indicating each UTR and TRS are not under neutral selection (c/µ ≠ 1). From Supplementary Fig. S1, 11 top NT mutations under strong beneficial selection were identified within the Orf1ab 5ʹUTR, Orf10 3ʹUTR, Orf7a 5ʹUTR, and N 5ʹUTR [in order of decreasing c/µ (3.0 to 178.1): C241T, G2794T, G210T, G174T, A28271T, T29834A, A28272T, C222T, C203T, and G29543T] (Table 2).

**Table 2.** Top mutations identified within ORF1ab 5ʹUTR, Orf7a 5ʹUTR, N 5ʹUTR, and ORF10 3ʹUTR, their c/μ values, and reported effects on SARS-CoV-2 fitness

Segment	Top Mut^a^	c/μ	Mutation effect^a^	Ref^a^
Orf1ab 5ʹUTR	C241T	178.1	May modulate viral packaging and titer volumes; co-mutation with NSP3-C3037T, NSP12-C14408T attenuated viral replication	Zhang et al. 2021
Orf10 3ʹUTR	G29742T	33.5	N/A	N/A
Orf1ab 5ʹUTR	G210T	31.8	Predicted to have reduced binding properties to RNA-binding protein SRSF7	Horlacher et al. 2023
Orf7a 5ʹUTR	C27874T	21.2	N/A	N/A
Orf1ab 5ʹUTR	G174T	8.5	N/A	N/A
N 5ʹUTR	A28271T	6.3	N/A	N/A
Orf10 3ʹUTR	T29834A	5.5	N/A	N/A
N 5ʹUTR	A28272T	4.9	N/A	N/A
Orf1ab 5ʹUTR	C222T	4.9	N/A	N/A
Orf1ab 5ʹUTR	C203T	4.7	N/A	N/A
Orf10 3ʹUTR	G29543T	3.0	N/A	N/A

a The UTR mutation NT residue number is relative to the genome position.

N/A: Not available.

No other top mutations were observed in the remaining UTRs or TRSs. Single-point mutations occurring within the UTRs of SARS-CoV-2 reportedly modulate viral pathogenicity, replication, and immune escape (Chan et al. 2020, Mishra et al. 2020, Young et al. 2020, Zhang et al. 2021). Moreover, Zhang et al. (2021) reported that SARS-CoV-2 plasmid constructs containing Orf1ab 5ʹUTR-C241T, NSP3-C3037T, and NSP12-C14408T mutations significantly reduced SARS-CoV-2 viral replication, which may be partially driven by changes in the Orf1ab 5ʹUTR secondary structure. Chaudhari et al. (2021) utilized in-silico docking and molecular dynamics simulation analysis to investigate this decreased SARS-CoV-2 replication due to Orf1ab 5ʹUTR C241T mutation, and indicated the mutant exhibits weaker binding affinity to host transcription factors MADP1 and hnRNPA1 (normally mediates viral replication), due to destabilized Orf1ab 5ʹUTR stem loop 1 interactions. Horlacher et al. (2023) conducted in-silico analysis of the Orf1ab 5ʹUTR mutation G210T in Delta and kappa SARS-CoV-2 strains, and predicted the mutation reduced the binding affinity of Orf1ab 5ʹUTR to the RNA-binding protein SRSF7, which may alter host cell splicing events. To summarize, about 18% of the top mutations under strong beneficial selection in Orf1ab 5ʹUTR and Orf10 3ʹUTR were identified in the literature and their fitness changes were inferred computationally.

### Ks/µ > 3 identified the top 107 synonymous mutations in TR that could impact viral RNA architectures, transcription, and translation

c/µ and Ks/μ were calculated for each AA site in each coding segment (Supplementary Figs S4–S5). Numerous Ks/µ peaks were observed in the synonymous sites of certain segments (e.g. S, M, Orf8, NSP1, NSP3, NSP11), indicating the synonymous sites in TR are not under neutral selection (Ks/µ ≠ 1). From Supplementary Figs S4–S5, 107 top synonymous mutations under strong beneficial selection were identified in S(12), M(4), N(11), Orf3a(2), Orf6(1), Orf8(2), Orf10(0), NSP1(3), NSP2(9), NSP3(19), NSP4(4), NSP5(4), NSP6(2), NSP7(0), NSP8(0), NSP9(5), NSP10(0), NSP11(11), NSP12(6), NSP13(6), NSP14(4), NSP15(2), with c/µ ranging from 0.03 to 82.23 and Ks/µ ranging from 3.00 to 610.78 (Supplementary Table S3). No top mutations were identified in Orf10, NSP7, NSP8, or NSP10. From this, 70.1% of the top synonymous mutations were identified in the NSPs, with which NSP11 contained 29.9% of the total synonymous mutations. This observation could suggest that nonsynonymous mutations are less tolerated in the NSPs, which mainly have roles in viral replication.

Since no mutation effects could be confirmed within the literature, the top synonymous sites (i.e. 3rd NT position within a codon) were mapped to their predicted RNA secondary structures to infer their mutation effect on RNA structure and chemical interaction networks (Supplementary Figs S8a–S8x). We chose to map the 3rd NT position of each codon as missense mutations within this position are more likely to be synonymous than missense mutations in the 1st or 2nd NT positions (more likely to be nonsynonymous); this is based on the codon structure, where mutations in the 1st and 2nd NT positions are more likely to change the resulting AA sequence, whereas mutating the 3rd NT position is more likely to change just the NT sequence. We visually analyzed whether each top synonymous mutation occurs within base-pairing interactions (not in a loop motif) or not (within a loop motif). In all, 44.8% of the top synonymous mutations are located within hydrogen bonding base pair interactions, whereas 55.2% of the top synonymous mutations were found neighboring other RNA nucleotides within loop structures. The location of these mutations may impact the thermostability or codon usage within the RNA structures, which will be revisited in the discussion section.

### C/µ >3 and Ka/Ks >2.5 identified the top 69 nonsynonymous adaptive mutations probably conferring immune escape and drug resistance in most coding proteins

c/µ and Ka/Ks were calculated for each AA site in each coding segment of TR (Supplementary Figs S6–S7). Only valid Ka/Ks values are shown for visual clarity. High Ka/Ks values were observed in sites where c/µ values were low in the major and accessory proteins (Supplementary Fig. S4) and NSP1-15 (Supplementary Fig. S6); while non-overlapping c/µ and Ka/Ks sites are not technical false beneficials (Ks = 0), this indicates the total mutation rate at these sites is low, and Ka/Ks can still become inflated due to low Ks. Therefore, the selection type for these sites inferred by Ka/Ks should be interpreted cautiously.

After sorting by c/µ + Ka/Ks, the 69 top nonsynonymous mutations under strong beneficial selection, weak beneficial selection, and weak purifying selection were identified in S (32), N (7), E (1), M (1), Orf3a (4), Orf7a (2), Orf8 (3), NSP2 (1), NSP3 (9), NSP4 (3), NSP5 (4), NSP6 (3), NSP11 (7), NSP12 (2), and NSP13 (1) (Table 3 and Supplementary Table S1), whereas no top mutations were identified in Orf6, Orf10, NSP1, NSP7-10, or NSP14-15. We also discuss several mutations of critical importance which do not meet our c/µ + Ka/Ks criteria for strong beneficial selection due to the timespan of our datasets. For simplicity’s sake, several notable top nonsynonymous mutations identified using our c/µ + Ka/Ks criteria in S, N, NSP11, and NSP5 (Table 3) will be examined as case studies, as they are implicated in viral pathogenicity, infectivity, and drug resistance mechanisms. The mutation effects for proteins not mentioned here in the main text are placed in the supporting document (Supplementary Table S3) (Aiewsakun et al. 2021, Azzeri et al. 2024, Brinkac et al. 2022, Cruz and Medina 2022, Ghosh et al. 2022, Lin et al. 2023, McGrath et al. 2024, Mou et al. 2021, Rashid et al. 2021, Saha et al. 2021, Wang et al. 2021b, Wu et al. 2021b, Zhang et al. 2022).

**Table 3.** Identified top nonsynonymous mutations in the spike glycoprotein (S), nucleocapsid (N), RNA-dependent RNA polymerase (NSP11), and 3CL-Protease (NSP5) of SARS-CoV-2, their position-based c/µ, Ka/Ks, Ka/µ and Ks/µ values, and reported mutation effects

Seg	Domain^d^^,^^e^^,^^f^^,^^g^	TopMut^b^	c/µ^a^	Ka/Ks^a^^,^^c^	Ka/µ^a^	Ks/µ^a^	Mutation effect	Ref
**S**	**RBD**	**D614G**	**68.67**	**Ks = 0!**	**87.68**	**0.00**	**Increased neutralization, viral replication, vaccine susceptibility**	Plante et al. 2021, Liu et al. 2022a
**S**	**RBD**	**P681H**	**34.05**	**Ks = 0!**	**48.76**	**0.00**	**Increased Moderna and Pfizer mRNA vaccine resistance**	Wang et al. 2021a
**S**	**RBD**	**N501Y**	**24.16**	**516.07**	**27.31**	**0.05**	**Increased replicative fitness, infection, antibody resistance**	Liu et al. 2022c, Liu et al. 2022a
**S**	**S2**	**D1118H**	**17.17**	**350.34**	**19.31**	**0.06**	**Decreased replicative fitness**	Liu et al. 2022c, Liu et al. 2022a
**S**	**S2**	**T716I**	**16.86**	**Ks = 0!**	**24.81**	**0.00**	**Decreased replicative fitness**	Liu et al. 2022c, Liu et al. 2022a
**S**	**S1**	**A570D**	**16.81**	**608.95**	**24.25**	**0.04**	**Increased replicative fitness**	Liu et al. 2022c, Liu et al. 2022a
**S**	**S2**	**S982A**	**16.71**	**966.59**	**28.54**	**0.03**	**Decreased replicative fitness**	Liu et al. 2022c, Liu et al. 2022a
**S**	**RBD**	**L452R**	**14.74**	**211.23**	**26.33**	**0.12**	**Increased weight loss, antibody resistance**	Liu et al. 2022a
**S**	**RBD**	**T478K**	**12.83**	**970.56**	**18.77**	**0.02**	**Increased susceptibility to BNT162b2 mRNA vaccine**	Liu et al. 2021a
**S**	**S2-HR1**	**D950N**	**11.62**	**237.12**	**13.07**	**0.06**	**Enhances S cleavage mechanism**	Furusawa et al. 2023
**S**	**NTD**	**T19R**	**11.61**	**Ks = 0!**	**17.25**	**0.00**	**Reduced viral infection and S processing**	Pastorio et al. 2022
**S**	**RBD**	**E484K**	**11.07**	**258.05**	**12.64**	**0.05**	**Significantly increased weight loss**	Liu et al. 2022a
**S**	**RBD**	**K417N**	**7.05**	**Ks = 0!**	**8.11**	**0.00**	**Increased BNT162b2 mRNA vaccine susceptibility**	Liu et al. 2022a
**S**	**NTD**	**L18F**	**6.06**	**236.75**	**8.94**	**0.04**	**Decreased S2L28 antibody binding to NTD**	McCallum et al. 2021
**S**	**NTD**	**G142D**	**5.87**	**Ks = 0!**	**8.69**	**0.00**	**Increased viral load, immune evasion**	Shen et al. 2021
**S**	**S2-HR2**	**V1176F**	**5.01**	**Ks = 0!**	**7.43**	**0.00**	**Increased patient mortality**	Majumdar and Niyogi 2021
**S**	**NTD**	**D138Y**	**4.82**	**24.66**	**5.40**	**0.22**	**Increased antibody resistance**	Alkhatib et al. 2021
**S**	**S2**	**T1027I**	**4.75**	**Ks = 0!**	**7.09**	**0.00**	**N/A**	N/A
**S**	**NTD**	**T95I**	**4.67**	**Ks = 0!**	**6.97**	**0.00**	**Increased viral load, immune evasion**	Shen et al. 2021
**S**	**NTD**	**T20N**	**4.64**	**4.36**	**6.21**	**1.42**	**N-glycosylation, increased antibody resistance**	Pegg et al. 2023
**S**	**NTD**	**P26S**	**4.55**	**370.95**	**6.82**	**0.02**	**Associated with higher transmission, reinfection, antibody resistance**	Gómez et al. 2021
**S**	**S1**	**H655Y**	**4.26**	**43.64**	**4.78**	**0.11**	**Associated with decreased S furin cleavage, pathogenicity**	Park et al. 2023
**S**	**S2**	**A701V**	**4.00**	**Ks = 0!**	**6.00**	**0.00**	**Associated with decreased S furin cleavage, pathogenicity**	Ou et al. 2022
**S**	**NTD**	**R190S**	**3.94**	**44.85**	**5.01**	**0.11**	**N-glycosylation, increased antibody resistance**	Pegg et al. 2023
**S**	**NTD**	**D80A**	**3.60**	**77.06**	**4.06**	**0.05**	**Associated with reduced CD4^+^ immune response**	Riou et al. 2022
**S**	**NTD**	**D215G**	**3.28**	**8.64**	**3.66**	**0.42**	**Increased antibody escape**	Wibmer et al. 2021
**S**	**NTD**	**A222V**	**3.28**	**Ks = 0!**	**4.91**	**0.00**	**Increased RBD-ACE2 binding**	Ginex et al. 2022
S	RBD	S477N	1.76	8.88	1.96	0.22	Increased weight loss, antibody resistance	Liu et al. 2022a
S	RBD	F490S	1.29	Ks = 0!	1.45	0.00	Increased resistance to neutralizing human immune sera	Liu et al. 2021b
S	RBD	N439K	1.16	23.65	1.30	0.06	Increased RBD-hACE2 binding affinity	Thomson et al. 2021
S	RBD	R346K	0.90	Ks = 0!	1.20	0.00	Increased lung pathogenicity/damage	Mohandas et al. 2022
S	RBD	N440K	0.42	Ks = 0!	0.47	0.00	Increased weight loss and antibody resistance	Liu et al. 2022a
**N**	**LKR**	**G204R**	**36.42**	**Ks = 0!**	**61.05**	**0.00**	**Increased infectivity**	Wu et al. 2021a
**N**	**LKR**	**S235F**	**16.97**	**58.14**	**24.32**	**0.42**	**Modulates N-epitope and vaccine-induced immune response**	Mohammad et al. 2021b
**N**	**CTD**	**D377Y**	**12.43**	**86.32**	**13.99**	**0.16**	**Increased viral titers and increased hospitalization risk**	Alsuwairi et al. 2023
**N**	**NTD**	**D63G**	**11.32**	**Ks = 0!**	**12.99**	**0.00**	**Increased viral titers and increased hospitalization risk**	Alsuwairi et al. 2023
**N**	**LKR**	**G215C**	**7.59**	**Ks = 0!**	**11.22**	**0.00**	**Predicted to stabilize N oligomerization, mediate viral assembly**	Kubinski et al. 2024
**N**	**LKR**	**T205I**	**6.95**	**20.83**	**10.11**	**0.49**	**Enhanced diagnostic evasion**	Bourassa et al. 2021
**N**	**NTD**	**P80R**	**4.18**	**173.30**	**6.29**	**0.04**	**Predicted to increase N-ssRNA binding affinity, facilitate viral assembly**	Manish et al. 2024
**NSP11**	**Interface**	**P323L**	**68.44**	**Ks = 0!**	**102.48**	**0.00**	**Increased viral RNA binding affinity and infectivity, Slightly decreased Remdesivir and GS-441 524 resistance**	Pitts et al. 2022, Takashita et al. 2022, Kim et al. 2023
**NSP11**	**Fingers**	**G671S**	**11.80**	**Ks = 0!**	**17.23**	**0.00**	**Increased viral RNA binding affinity and infectivity, slightly increased Remdesivir and GS-441 524 resistance**	Pitts et al. 2022, Kim et al. 2023
**NSP11**	**NiRAN**	**P227L**	**3.28**	**67.13**	**4.90**	**0.07**	**N/A**	N/A
NSP11	NiRAN	A97V	0.66	Ks = 0!	0.99	0.00	Slightly impacts RdRp secondary structure	Mohammad et al. 2021a
NSP11	Palm	V776L	0.58	22.97	0.84	0.04	Decrease structural flexibility in NSP8–NSP11 interaction region	Kumar et al. 2022
NSP11	NiRAN	A185V	0.57	46.00	0.85	0.02	N/A	N/A
NSP11	Fingers	M666I	0.45	Ks = 0!	0.45	0.00	N/A	N/A
NSP11	Palm	V792I	0.00	Ks = 0!	0.00	0.00	Increased Remdesivir resistance	Tanino et al. 2024, Hogan et al. 2022
NSP11	Palm	E802D	0.00	Ks = 0!	0.00	0.00	Increased Remdesivir resistance	Gandhi et al. 2022
**NSP5**	**Chy1**	**K90R**	**4.15**	**99.50**	**4.76**	**0.05**	**Modulated MPro drug resistance, decreased enzyme efficiency**	Bei et al. 2023b, Greasley et al. 2022, Ullrich et al. 2022, Kawashima et al. 2023, Jiang et al. 2023
NSP5	Chy1	G15S	2.42	96.62	3.59	0.04	Modulated MPro drug resistance, decreased enzyme efficiency	Bei et al. 2023b, Greasley et al. 2022, Ip et al. 2023, Jiang et al. 2023, Kawashima et al. 2023
NSP5	Chy1	L89F	1.36	109.47	2.02	0.02	Modulated MPro drug resistance	Iketani et al. 2023, Bei et al. 2023, Jiang et al. 2023, Kawashima et al. 2023
NSP5	Chy1	T21I	0.73	59.35	1.09	0.02	Modulated MPro drug resistance	Ullrich et al. 2022, Bei et al. 2023
NSP5	Pc3CPro2	C145A/S	0.00	Ks = 0!	0.00	0.00	Disrupts critical C145-H41 dyad interaction; inactivates NSP5	Ferreira et al. 2021
NSP5	Chy1	H41A/D/E	0.00	Ks = 0!	0.00	0.00	Disrupts critical C145-H41 dyad interaction; inactivates NSP5	Ferreira et al. 2021

a Bolded values represent mutations which meet the c/µ and Ka/Ks criteria for strong beneficial selection (c/µ > 3 and Ka/Ks > 2.5). Unbolded values represent mutations included for their reported beneficial fitness change despite not satisfying our c/µ and Ka/Ks criterion.

b TopMut: Top nonsynonymous mutation relative to the gene position. See Supplementary Data for mutation position relative to the TR.

c Ks = 0! Indicates a technical error when calculating Ka/Ks for the AA site. These sites were still recorded under strong beneficial selection.

¤Selection types as per c/µ criterion: strong beneficial selection (c/µ > 3), weak beneficial selection (1 > c/µ > 3), weak purifying selection (0.5 ≥ c/µ > 1), and strong purifying selection (c/µ < 0.5).

d S protein domains: S1 domain (AA 14-667), NTD: N-terminus domain (AA 14-290), RBD: Receptor binding domain (AA 319-541); S2 domain (668-1273), HR1: Heptad repeat 1 (AA 942-990); HR2: Heptad repeat 2 (AA 1163-1202).

e N protein domains: N-arm (AA 1-43); NTD: N-terminal domain/RNA-binding domain (AA 44-174); LKR: Linker region (AA 175-254); CTD: C-terminal domain/Dimerization domain/RNA-binding domain (AA 255-364).

f NSP11 protein domains: NiRAN: Nucleotidyl transferase domain (AA 115-250); Interface (AA 251-365); Fingers (AA 397-581, 621-679); Palm (582-620, 680-815).

g NSP5 protein domains: Chy1: Chymotrypsin-like domain 1 (AA 8-101); Pc3CPro2: Picornavirus 3C protease-like domain (AA 102-184); GC3: globular cluster domain (AA 201-303).

See Supplementary Tables S1–S2 for the top non-synonymous and synonymous mutations for the remaining proteins.

### Top 32 nonsynonymous S protein adaptive mutations confirmed to increase SARS-CoV-2 replicative fitness and reduce vaccine–antibody neutralization

**Figure 3.**
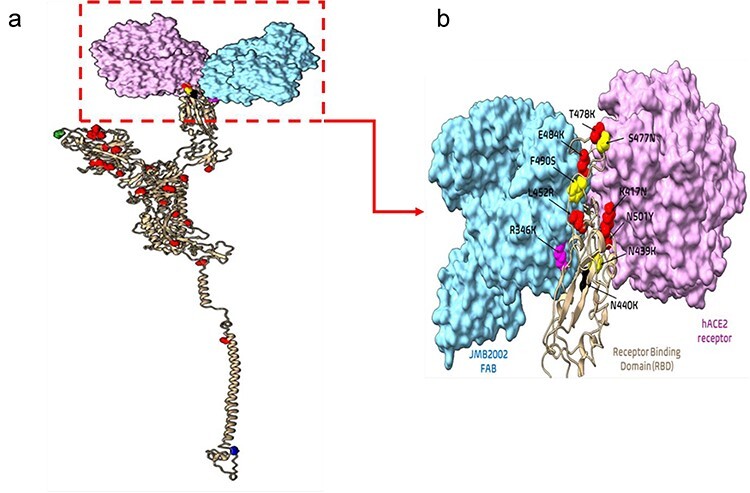
Top 32 nonsynonymous mutations in the S protein (cream ribbons) complexed with JMB2002 Fab antibody (blue surface) and hACE2 receptor (purple surface).

The S protein mediates viral entry into the host cell by binding its receptor binding domain (RBD-S1 domain) to a host cell receptor, such as hACE2, following viral membrane fusion (Beyerstedt et al. 2021). As current SARS-CoV-2 vaccines are constructed from a modified S protein sequence, identifying breakthrough mutations leading to vaccine resistance is critical for preventing severe COVID-19 infection. Encouragingly, c/µ + Ka/Ks identified 32 top nonsynonymous mutations (in order of decreasing c/μ: D614G, P681H, N501Y, D1118H, T716I, A570D, S982A, L452R, T478K, D950N, T19R, E484K, K417N, L18F, G142D, V1176F, D138Y, T1027I, T95I, T20N, P26S, H655Y, A701V, R190S, D80A, D215G, A222V, S477N, F490S, N439K, R346K, and K440K) (Table 3, Fig. 3) and are all located within the S1 region (N-terminal domain/NTD and Receptor-binding domain/RBD) and S2 region (including Heptad-Repeat 1/HR1 and Heptad-Repeat 2/HR2).

The top nonsynonymous mutations are mapped within the entire S protein structure (Fig. 3A) and within the RBD (Fig. 3B). While the first 27 top mutations are under strong beneficial selection (c/µ: 3.28-68.67), S477N, F490S, and N439K are under weak beneficial selection (c/µ: 1.16 to 1.76), R346K is under weak purifying selection (c/µ: 0.90), and K440N is under strong purifying selection (c/µ: 0.42). S477N, F490S, N439K, R346K, and K440N were included due to their positive impacts on antibody resistance and hACE2 binding (Liu et al. 2021b, 2022a, Mohandas et al. 2022, Thomson et al. 2021). To note, 14 out of 32 top mutations exhibit technical error (Ks = 0), while the remaining 18 top mutations exhibited valid Ka/Ks values (Ka/Ks: 4.36 to 970.56). A total of 31 out of the 32 mutations (∼97%) were experimentally characterized as endowing positive fitness changes to SARS-CoV-2, including increased infectivity (Furusawa et al. 2023, Ginex et al. 2022, Gómez et al. 2021, Liu et al. 2022a, 2022c), replicative fitness (Liu et al. 2022a), pathogenicity (Majumdar and Niyogi 2021, Liu et al. 2022a), and vaccine/antibody/immunity resistance (Alkhatib et al. 2021, Gómez et al. 2021, Liu et al. 2022a, McCallum et al. 2021, Pegg et al. 2023, Riou et al. 2022, Shen et al. 2021, Wang et al. 2021a, Wibmer et al. 2021); some studies report reduced pathogenicity (Ou et al. 2022, Park et al. 2023), vaccine/antibody susceptibility (Plante et al. 2021, Liu et al. 2021a), and replicative fitness (Liu et al. 2022a). Several mutations will be discussed in detail to simplify the discussion.

Reverse genetic engineering and single-point mutagenesis studies are commonly utilized to help characterize their phenotypic changes, including infectivity (Liu et al. 2021a, 2022a, 2022c, Plante et al. 2021), and will comprise the bulk of evidence for molecular adaptation due to the top mutations in S. Plante et al. (2021) investigated vaccine escape and replicative fitness of D614G in mutant S proteins. D614G displayed higher neutralization titers (1.4 to 2.3-fold increase) in hamster sera, thus increasing vaccine potency, and faster viral replication rates (2.1-8.6-fold increase) in human airway tissues (Plante et al. 2021). Additionally, initial D614:G614 virus ratios at the start of infection (1:1, 3:1, and 9:1) in human airway tissues demonstrated rapid rises in the relative G614 population 5 days post infection (d.p.i.) (13.9, 9.1, and 5, respectively), suggesting D614G variants, even as a minority in the viral population, vastly outcompete wild-type SARS-CoV-2 (Plante et al. 2021). In a similar study, Liu et al. (2021a) conducted 50% Plaque Reduction Neutralization Tests (PRNT50) on human sera immunized with two doses of BNT162b2 mRNA vaccine before challenge by modified B.1.351 SARS-CoV-2 variants containing K417N, E484K, N501Y, and D614G mutations. PRNT50 values significantly decreased in B.1.351-spike (PRNT50 = 194) and B.1.351-RBD + D614G-spike (PRNT50 = 331) compared to the wild-type USA-WA1/2020-spike (PRNT50 = 532), demonstrating K417N, E484K, D614G, and N501Y increased SARS-CoV-2 susceptibility to the BNT162b2 vaccine and quelling concerns (Liu et al. 2021a).

In another study, Liu et al. (2022c) elucidated the replicative fitness change from the N501Y mutation in a hamster model. The absolute replicative fitness of N501Y mutant compared to the wild-type strain increased slightly in hamster nasal washes (Y501: 3.3-fold increase; N501: 2.8-fold increase), trachea (Y501: 5.3-fold increase; N501: 3.5-fold increase), and lung sera (Y501: 2.3-fold increase; N501: 2.0-fold increase) (Liu et al. 2022c). Lastly, Liu et al. (2022a) measured the pathogenicity α-S (E484K), δ-S (L452R, T478K, D614G and D950N), ο-S (K417N, N440K, S477N, T478K, N501Y and D614G), β-S, and ε-S SARS-CoV-2 chimeras in hamster models. α-S caused the greatest total weight loss (∼18% body weight) 8 d.p.i. compared to the remaining strains (∼1% to ∼5%), indicating E484K is highly pathogenic (Liu et al. 2022a). Furthermore, 50% Fluorescent Foci Reduction Neutralization Test (FFRNT50) analysis revealed a 62- to 9.7-fold relative difference in neutralizing titers between the chimeric S variants (up to 22-fold increase) and wild-type S (up to 2.2-fold increase) 45 d.p.i., indicating that E484K, K417N, N440K, L452R, S477N, T478K, N501Y, and D614G confer high antibody resistance (Liu et al. 2022a).

In summary, the fitness change inferred by c/µ and Ka/Ks for the top mutations under strong beneficial selection (D614G, L452R, T478K, N501Y, E484K, and K417N) were confirmed in the literature, correlated with increased antibody resistance, increased pathogenicity, and replicative fitness. In contrast, D614G seemed to confer increased susceptibility to vaccines.

### Top 9 nonsynonymous adaptive mutations in SARS-CoV-2 nucleocapsid (N) increase infectivity, hospitalization risk, and diagnostic evasion

**Figure 4.**
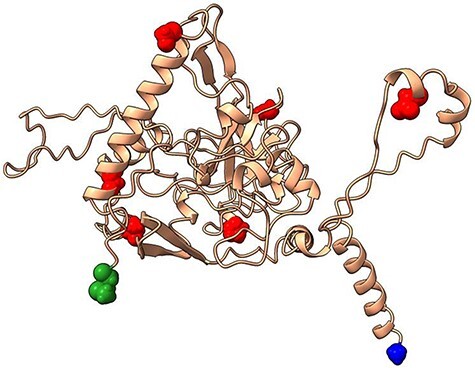
Top seven mutations (red balls) under strong beneficial selection in the N protein (cream ribbons).

The N protein encapsulates and protects the SARS-CoV-2 viral genome through its journey to the host cytosol, facilitating viral assembly and mature virion formation (Bai et al. 2021). The N protein is also an antigenic biomarker in several diagnostic tests (Nicollete et al. 2023) and has been proposed as a vaccine candidate (Hajnik et al. 2022, Primard et al. 2023). Identifying the top mutations leading to diagnostic evasion is critical for detecting the virus in human patients. Our combined c/μ and Ka/Ks approach identified the top seven nonsynonymous mutations (in order of decreasing c/μ: G204R, S235F, D377Y, D63G, G215C, T205I, and P80R) (Table 3). The top nonsynonymous mutations which are mapped in Fig. 4, are located in the NTD, CTD, and LKR regions. All the identified mutations are categorized as under strong beneficial selection (c/μ: 4.18 to 36.42). To note, three out of seven mutations exhibited Ks = 0, whereas the remaining four mutations exhibited valid Ka/Ks values (Ka/Ks: 20.83 to 173.30). All seven top mutations (100%) were either characterized experimentally (5/7 = 71%) or inferred through computational analysis (2/7 = 29%), suggesting beneficial fitness changes in SARS-CoV-2 N including increased infectivity and virulence (Wu et al. 2021a), modulating the immune response (Mohammad et al. 2021b), increased hospitalization and virion levels (Alsuwairi et al. 2023), and enhanced diagnostic evasion (Bourassa et al. 2021).

The following studies focus on diagnostic evasion and pathogenicity evaluation of N mutants of SARS-CoV-2. Bourassa et al. (2021) evaluated the detection of T205I mutation in BinaxNOW and Sofia2 antigen assays. BinaxNow successfully detected three out of three wild-type N samples and two out of three T205I N samples (1:100,000 viral dilution), indicating diagnostic evasion. Sofia antigen detection for T205I/D399N N samples decreased from four out of four samples (1:10 dilution), to two out of six samples (1:100 dilution) to 0 out of three samples (1:1000 to 1:100,000 dilution), also indicating diagnostic evasion (Bourassa et al. 2021). Wu et al. (2021a) utilized reverse genetic engineering to elucidate the fitness change of G204R mutation on N in mice and human lung cell models. The G2044 mutant increased the viral titer and E subgenomic RNA levels in hamster nasal wash sera compared to the wild-type strains, indicating greater replicative fitness (Wu et al. 2021a). Additionally, G204R mutant increased inflammation in hamster lung samples, suggesting higher pathogenicity (Wu et al. 2021a). Similarly, Kubinski et al. (2024) utilized a reverse genetics system to investigate the replication, pathogenesis, and nucleocapsid packing capabilities modulated by G215C in a hamster model. Relative to the wild-type strain, the G215C mutant elevated viral titer levels in hamster nasal washes and lung samples (10-fold increase), indicating greater replicative fitness (Kubinski et al. 2024). The G215C mutant also increased the pathogenicity and immune invasion, prompting subendothelial mononuclear cell invasion and severe epithelial cytopathology leading to peribrochiolitis (Kubinski et al. 2024). The G215C mutant promoted N-dimerization more than the wild-type strain, of which G215C N-dimers demonstrated higher viral encapsulation profiles compared to the wild-type N (Kubinski et al. 2024).

In summary, the top seven nonsynonymous mutations under strong beneficial selection were confirmed to increase N protein fitness by conferring diagnostic evasion, increasing replicative fitness and pathogenicity profiles and stabilizing N-dimers to make viral packing more efficient.

### Top seven nonsynonymous mutations in RdRp (NSP11) modulate protein stability and increase Remdesivir resistance in SARS-CoV-2

**Figure 5.**
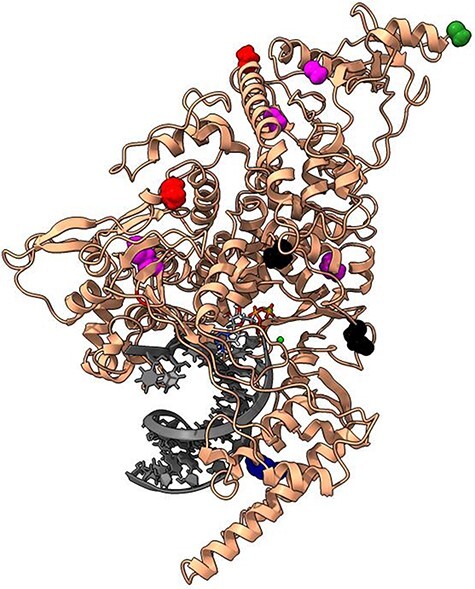
Top seven nonsynonymous mutations (red balls) and two conserved AA sites (black balls) in the NSP11 protein (cream ribbons) complexed with viral RNA (gray), remdesivir triphosphate (licorice colored by element) and Mg^2+^ cofactors (green balls).

NSP11 constitutes the viral RNA-dependent RNA polymerase (RdRp), which catalyzes the formation of a complementary strand from an RNA viral template, with several domains (palm, thumb, finger, NiRAN, and interface) facilitating this process (Hillen et al. 2020). Nucleoside analogs suppress NSP11 activity by competing with viral ribonucleotides for incorporation into the mRNA transcript, inducing delayed chain termination (e.g. Remdesivir TP) (Kokic et al. 2021) and G→A and C→U mutations to sabotage protein translation (e.g. Molnupiravir) (Yip et al. 2022). Identifying the top nonsynonymous mutations which enhance RdRp fitness through improved replication or drug resistance is therefore critical. Our combined c/µ and Ka/Ks approach identified the top seven nonsynonymous mutations in NSP11 (in order of decreasing c/µ: P323L, G671S, P227L, A97V, V776L, A185V, M666I, P323L, A185V, and M666I) (Table 3). The top nonsynonymous mutations which are mapped in Fig. 5, are located in the interface, fingers, and NiRAN domains, where P323L and G671S are located near the NSP11 active site.

Categorically, based on their c/µ values, P323L, G671S, and P227L are under strong beneficial selection (c/µ: 3.28 to 68.44), A97V, V776L, and A185V are under weak purifying selection (c/µ: 0.57 to 0.66), and M666I is under strong purifying selection (c/µ: 0.45). To note, six out of nine mutations exhibited Ks = 0, whereas the remaining three mutations exhibited valid Ka/Ks values (Ka/Ks: 22.97 to 67.13). Although the mutations V792I and E802D were completely conserved in our dataset (c/µ = 0.00), we justify their inclusion here due to their potential for spreading within the viral population, as inferred from their reported enhancement on RdRp activity and overcoming Remdesivir resistance (Gandhi et al. 2022, Hogan et al. 2022, Tanino et al. 2024). Otherwise, six out of nine mutations (∼66%) reported fitness changes to SARS-CoV-2 NSP11, including increased infectivity (Kim et al. 2023), Takashita et al. 2022), increased or decreased drug resistance (experimentally-characterized) (Gandhi et al. 2022, Hogan et al. 2022, Pitts et al. 2022, Tanino et al. 2024), or altered structure (computational analysis) (Kumar et al. 2022, Mohammad et al. 2021a).

Kim et al. (2023) investigated activity change, polymerase–substrate complex stability, viral kinetics, and replication capacity due to P323L, G671S, and P323L/G671S mutations. They reported a temperature-dependent change in RdRp activity following P323L (∼4-fold increase), G671S (∼2-fold increase), and P323L/G671S co-mutation (∼5-fold increase) at 33°C, whereas the activity of the mutants dropped to the WT levels at 37°C (Kim et al. 2023). Next, binding affinity analysis of RdRp to viral RNA revealed a significant decrease in K_D_ in the P323L (81 nM, 0.27-fold), G671S (74 nM, 0.24-fold decrease), and P323L/G671S co-mutants (67 nM, 0.22-fold change) compared to the wild-type strain (298 nM), indicating enhanced RdRp-viral RNA binding and a more stable substrate–polymerase complex (Kim et al. 2023). Viral growth kinetics in ferret nasal turbinates revealed higher viral titers in P323L (∼4.1 log_10_TCID_50_/g, 1.1-fold increase), G671S (∼3.9 log_10_TCID_50_/g, 1.1-fold increase), and P323L/G671S (∼4.9 log_10_TCID_50_/g, 1.3-fold increase) mutants compared to those of the wild-type (∼3.7 log_10_TCID_50_/g). From this, growth competition assays from ferrets infected with a 1:1 WT-P323L virus seeding ratio revealed fast domination of the P323L population quickly from 0 d.p.i. (50%), 2 d.p.i. (∼80%) to 6 d.p.i. (∼93%) over the wild-type population, demonstrating the replicative advantage conferred by P323L (Kim et al. 2023).

Pitts et al. (Pitts et al. 2022) performed *in-vitro* characterization of remdesivir and GS-441 524 potency against the WA1-NanoLuciferase NSP11 wild-type and mutant (P323L and P323L/G671S) strains. Luciferase signaling revealed the relative change in effective concentration 50% (EC50) for Remdesivir (WT: 80 μM; P323L: 71 μM, 0.89-fold decrease; P323L/G671S: 104 μM, 1.3-fold increase) and GS-441 524 (WT: 1880 μM; P323L: 1580 μM, 0.84-fold decrease; P323L/G671S: 3450 μM, 1.83-fold increase), indicating P323L slightly enhances the potencies of these compounds, whereas the P323L/G671S slightly decreases their potencies. Using a different methodology, Takashita et al. (2022) examined NSP11 P323L mutation from human patient samples and characterized their impact on GS-441 524, molnupiravir, and PF-00835231 drug potency. Focus reduction neutralization assays revealed the relative EC50 changes between wild-type and mutant binding potencies for GS-4 415 245 (WT: 1.04 μM, P323L: 0.83-0.13 μM; 0.8-1.2-fold change), molnupiravir (WT: 0.51 μM; P323L: 0.41-3.95 μM; 0.8-1.9-fold change), and PF-00835231 (WT: 9.40-14.81 μM; P323L: 9.40-14.81 μM; 0.51-0.80-fold change). As in the Pitts et al. (2022) study, the mutation effect of P323L and G671S on nucleoside analog potencies is minor.

To date, the mutations P227L, A97V, V776L, A185V, and M666I have not been characterized experimentally; however, computational analysis has been performed to elucidate their mutation effects. The V776L mutation is predicted to decrease the flexibility of the interaction site between NSP8 and NSP11, which may increase their binding affinity and stabilize the replication complex (Kumar et al. 2022) and exhibit remdesivir resistance (Tanino et al. 2024). In-silico analysis suggests A97V mutation impacts RdRp structure, though further investigation has not been pursued (Mohammad et al. 2021a).

In summary, two of the top nonsynonymous mutations under strong beneficial selection (P323L and G671S) were confirmed to impact NSP11 fitness by conferring replicative advantages, stabilized replication assembly and viral RNA processing, and modulation of drug potencies.

### Top four nonsynonymous mutations in MPro (NSP5) modulate drug resistance in SARS-CoV-2

**Figure 6.**
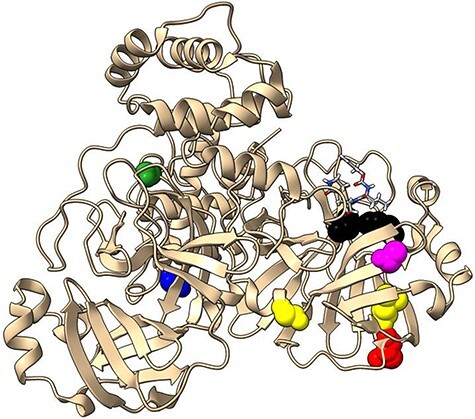
Top four nonsynonymous mutations (red, yellow, purple and black balls) in NSP5 protein (cream ribbons) complexed with nirmatrelvir (licorice colored by element).

NSP5 is the main protease (MPro) which cleaves the ORF1ab polyprotein into several NSPs critical for viral replication (Chen et al. 2022). NSP5 consists of three domains, of which the substrate binding site is located between domains 1 and 2, containing the critical Cys145–His41 dyad interaction driving Orf1ab cleavage (Ferreira et al. 2021). In fact, the FDA-approved MPro inhibitor Paxlovid has successfully prevented severe COVID-19 infection by inhibiting this mechanism via covalent binding to C145, blocking the C145–H41 interaction (Owen et al. 2021). Identifying mutations which drive MPro inhibitor resistance is critical for preventing severe COVID-19 infection. Our combined c/µ and Ka/Ks approach identified the top four nonsynonymous mutations in NSP5 (in order of decreasing c/μ: K90R, G15S, L89F, T21I) (Table 3) and were mapped to the wild-type homology model structure in Fig. 6, located in the first domain. Categorically, K90R is under strong beneficial selection (c/µ: 4.15), G15S and L89F are under weak beneficial selection (c/µ: 1.36 to 2.42), and T21I is under weak purifying selection (c/µ: 0.73). To note, all four mutations exhibit Ks > 0 and their respective Ka/Ks values are considered valid (Ka/Ks: 59.35 to 109.47). In Table 3, we also included two mutations induced within the catalytic dyad (C145A/S and H41A/D/E) which was investigated by Ferreira et al. (2021) but was conserved in our dataset (c/µ and Ka/Ks: 0.00) to demonstrate their critical importance in NSP5 function.

Numerous studies have investigated the impact of various MPro inhibitors on NSP5 mutations. Bei et al. (2023) utilized site-directed mutagenesis and a dual-reporter assay to characterize the drug potencies of GC376, S-217 622, and PF-07321332 on wild-type and mutant (G15S, T21I, L89F, and K90R) strains. Compared to the wild-type IC50 values (GC376: 2.13 µM; S-217 622: 0.67 µM; PF-07321332: 0.69 µM), the relative IC50 change dropped moderately in the G15S (GC376: 0.3-fold; S-217 622: 0.6-fold; PF-07321332: 0.3-fold), T21I (GC376: 0.5-fold; S-217 622: 0.8-fold; PF-07321332: 0.5-fold), L89F (GC376: 0.2-fold; S-217 622: 0.3-fold 0.67; PF-07321332: 0.3-fold), and K90R mutants (GC376: 0.2-fold; S-217 622: 0.3-fold; PF-07321332: 0.3-fold), indicating moderate drug resistance. Similarly, Greasley et al. (2022) characterized the *in-vitro* enzyme kinetics and drug potency of nirmatrelvir against the G15S and K90R mutations. FRET analysis of substrate peptide cleavage showed the catalytic efficiency [k_cat_ (turnover number)/K_m_ (Michaelis Menten constant)] of wild-type NSP5 (31,500 S^−1^ M^-1^) is better than those of G15S (16,483 S^−1^ M^-1^) and K90R (28,255 S^−1^ M^-1^). Moreover, the relative K_i_ changes with the G15S mutant (4.4-fold) and K90R mutant (1.1-fold) with respect to the wild-type NSP5 indicates slightly decreased binding affinity of nirmatrelvir to these mutants.

Our c/µ and Ka/Ks approach can also detect the functionally critical sites under strong purifying selection, such as the catalytic dyad C145-H41 in NSP5. Using site-directed mutagenesis, Ferreira et al. (2021) constructed a pET28b(+) bacterial plasmid to express wild-type and mutant NSP5 (C145A/S or H41A/D/E) and to characterize their ability to process a 25-mer peptide substrate. FRET-based enzymatic assays revealed the proteolytic rate of wild-type NSP5 at different enzyme concentrations (0.5–5.0 μM) increased in a concentration-dependent manner (12 s^−1^ at NSP5 = 5.0 µM); however, the C145A/S and H41A/D/E mutations completely inactivated NSP5 even at the highest enzyme concentration (0 s^−1^), indicating the wild-type C145 and H41 residues are undoubtedly critical to NSP5 function. Interestingly, Bei et al. (2023) also confirmed the complete loss of activity in the C145A mutant, treating it as a blank control relative to other mutants (G15S, T21I, L89F, K90R, P108S, P132H, and L205V).

To summarize, the fitness effects of the top four nonsynonymous mutations identified in NSP5 using our combined c/µ and Ka/Ks approach were confirmed in the literature, demonstrating these mutations modulate drug binding and catalytic efficiency.

## Discussion

Using a robust method to identify the top nonsynonymous and synonymous mutations under strong beneficial selection is paramount to elucidating their effects on viral proteins and nucleic acids, including UTRs. The conventional Ka/Ks analysis has been used to assess both temporal and spatial patterns in protein fitness change. We provide a comparison between this study and two other Ka/Ks studies which utilized Ka/Ks to identify the top nonsynonymous mutations within SARS-CoV-2 and other sarbecoronavirus genomes in Table 4.

**Table 4.** Literature comparison between top mutation detection methods and the number of top mutations identified versus the methods employed in this study

Ref	Method	Genomic datasets	#TopMutation Sites
Habib et al. 2023	**Ka/Ks** (Position-based)	311 SARS-CoV-2 genomes (Human) (Nov. 2021–April 2023)	**Nonsynonymous: 26** (S, M, Orf3c, Orf6, Orf9b, Orf9c, NSP3, NSP6)
Tang et al. 2023	**Ka/Ks** (Position-based)	7,269,791 million sarbecoronavirus genomes (Human, Bovine, Murine, Avian, Canine, Feline, Bat, Camel, Porcine) (Nov. 2019–Nov. 2020)	**Nonsynonymous: 186** (S, E, M, N, NSP2, NSP3 and NSP12)
This study	**c/µ, Ka/Ks, Ks/µ** (Position-based)	11,198 SARS-CoV-2 genomes (Human) (Dec. 2019–June 2021)	**Nonsynonymous: 69** (S, E, M, N, Orf3a, Orf7a, Orf8, NSP2, NSP3, NSP4, NSP5, NSP6, NSP11, NSP12, NSP13) **Synonymous: 107** (S, M, N, Orf3a, Orf6, Orf8, NSP1, NSP2, NSP3, NSP4, NSP5, NSP6, NSP9, NSP11, NSP12, NSP13, NSP14, NSP15) **UTR: 11** (Orf1ab 5ʹUTR, Orf10 3ʹUTR, Orf7a 5ʹUTR, N 5ʹUTR)

Habib et al. (2023) utilized Ka/Ks based on the random-sites model 8 to allow for beneficial selection on 311 SARS-CoV-2 Omicron genomes from Bangladesh, and identified 26 total nonsynonymous sites under strong beneficial selection (Ka/Ks > 8) in S, M, and ORF6 genes. Tang et al. (2023) utilized Ka/Ks and recorded >100 nonsynonymous sites from S, E, M, N, NSP2, NSP3, and NSP12 genes of 26 closely related coronaviruses including SARS-CoV-2. Najar et al. (2023) calculated the Ka, Ks, and Ka/Ks values for the SARS-CoV-2 genome and each coding gene for 6.4 million viral sequences (November 2019 to November 2022) to evaluate Ka or Ka/Ks as a reliable predictor for infection surge events. Their data reveal Ka exhibited a clear signal increase for S, M, and E proteins during the Omicron infection surge, whereas Ka/Ks produced very noisy signals for S, M, E, and NSP11 proteins (November 2019 to May 2021). To our suspicion, the COVID-19 Surge Prediction Tool (https://pandemics.okstate.edu/covid19/index.html) revealed that the Ks values for S, E, M, and NSP11 were close to zero during the early pandemic phase (November 2019 to November 2020), which likely generated the noisy Ka/Ks signal and justified the use of Ka as the infection surge parameter. While Ka/Ks provides useful insights into the overall protein fitness change, it can underrepresent the fitness change in synonymous sites of nucleic acid sequences and can inflate the Ka parameter in sites where Ks ≈ 0, potentially misrepresenting sites as being under strong adaptive selection. Moreover, Ka/Ks is difficult to assess in a site-by-site approach. Indeed, we confirmed this for each codon in All-TR, showing most sites exhibiting an undefined Ka/Ks due to Ks = 0 or Ka and Ks = 0 (Table 1).

To enhance the detection of top mutations in both UTR and TR, we utilized our c/µ test to identify the top 11 mutations in UTR and combined both c/μ and Ka/Ks and Ks/µ to identify the top 69 nonsynonymous and top 107 synonymous mutations in the TR, respectively, using SARS-CoV-2 genomic datasets in our previous reports. We then conducted an exhaustive literature report of the phenotypic and genotypic changes of SARS-CoV-2 mutants exhibiting these top mutations to link the fitness change inferred from high c/μ, Ks/μ, and Ka/Ks values. Encouragingly, 18% of the top mutations in UTR and 70% of the top nonsynonymous mutations in TR had reported or predicted effects in the literature; specifically, most top nonsynonymous mutations in S, N, NSP11, and NSP5 beneficially enhanced their function, supporting our combined c/μ and Ka/Ks approach in identifying sites under strong adaptive selection. Yet, the mutation effects in coding genes M (I82T), Orf7a (V82A), Orf8 (Y73C), NSP3 (A890D, A488S, K977Q and S370L), NSP4 (A446V), and NSP13 (A394V) and non-coding UTR are uncharacterized. This might be due to mutations within the S, NSP11, and NSP5 proteins being prioritized above the remaining proteins within the general community, since they are targets for vaccination and small molecule inhibitors, and resistance to either therapy remains a concern. Experimental methods such as reverse genetic engineering can help characterize these mutations that have been known since the early SARS-CoV-2 pandemic.

While the fitness change of the top 107 synonymous mutations were unreported in the literature, they could have implications in viral nucleic acid processes (in this context, RNA), including codon optimization, transcription, and translation (Supplementary Table S3). The proportion of top synonymous mutations found within base-pairing interactions (44.8%) or loop regions (55.2%) suggests there is no preference for their placement in either region (Supplementary Figs S8a–S8x). Aside from this, mutations can either strengthen base-pairing interactions and increase nucleic acid thermostability or weaken them and cause bulge or loop structures to appear. The presence of RNA motifs can modulate the accessibility of nucleotide binding sites with viral polymerases or host ribosomes, thereby altering transcription and translation processes. Rigby et al. (2023) revealed the destabilization of T-loops, transient RNA structures which form around the viral polymerase, in evolving SARS-CoV-2 and Influenza A virus genomes, mediating host cell adaptation and hastening viral replication. Zhang et al. (2023) demonstrated that synonymous codon optimization of SARS-CoV-2 S mRNA via their algorithm LinearDesign enhanced the potency of the SARS-CoV-2 mRNA vaccine by >128-fold in infected mice. Furthermore, viral codon usage in different host species (e.g. mink, bat, human) is analyzed through synonymous mutations to assess their molecular evolution and fitness change (Yanan et al. 2022, Kumar et al. 2021, Mogro et al. 2022). Ramazzotti et al. (2022) examined 400,000 SARS-CoV-2 genomic samples (March 2020 to September 2021) containing more than 3 million synonymous mutations and demonstrated a temporal increase toward matching SARS-CoV-2 codon usage with human codon usage, suggesting host adaptation. From this, our combined c/µ + Ks/µ approach can help shed light on correctly identifying top synonymous mutations in TR to assess their molecular effects *in vitro* and *in vivo*. Biochemical and computational analysis of one and/or several synonymous mutations in coding structures is recommended to elucidate their fitness effects.

The fitness changes due to non-synonymous mutations could be due to multiple factors such as drug and antibody escape, viral capsid assembly, interactions with host DNA and transcription factors, epistatic interactions, and others. To probe the drug and antibody escape effects, we utilized molecular docking to determine the ligand binding affinity change between wild-type and top mutant protein targets as mentioned in the “Methods” section. The resulting change in ligand binding free energy between mutant and wild-type structures (ΔΔG_bind_) and the individual ligand binding free energy between mutant and wild-type structures (ΔG_bind_) are tabulated in Supplementary Tables S3 and S4. The top mutations generally exhibited a beneficial impact on viral protein fitness through their ΔΔG_bind_ values. In S, all mutations except N440K increased binding affinity toward hACE2 (−0.1 to −1.3 kcal/mol), and all mutations except L452R and K417N increased binding affinity toward the JMB2002 Fab (−1.4 to +0.5 kcal/mol). The JMB2002 Fab, which was demonstrated to have enhanced binding affinity toward numerous SARS-CoV-2 variants (Alpha-Omicron) (Yin et al. 2022a), demonstrates the encouraging notion that beneficial viral fitness can be overcome with human innovation. Considering NSP11, the FDA-approved inhibitors Remdesivir TP, Favipiravir TP, Ribavirin TP, Galidesivir TP, Sofosbuvir T,P and Dasabuvir mostly saw decreased binding affinity with N501Y (−0.5 to −1.0 kcal/mol), L452R (+0.5 to −1.0 kcal/mol), T478K (−0.1 to −0.3 kcal/mol), E484K (−0.3 to −1.0 kcal/mol), K417N (−0.2 to +1.1 kcal/mol), S477N (−0.1 to −0.7 kcal/mol), F490S (−1.3 to −1.4 kcal/mol), N439K (−0.3 to −0.7 kcal/mol), R346K (<−0.0 kcal/mol), and N440K (<0.0 to +0.2 kcal/mol). In NSP5, the FDA-approved inhibitors Nirmatrelvir, GC376, Lufotrelvir, and Ensitrelvir saw mixed binding affinity changes with G15S (0.0 to +0.6 kcal/mol), K90R (−0.9 to +0.1 kcal/mol), L89F (−0.9 to +0.1 kcal/mol), and T21I (−0.5 to +0.9 kcal/mol). From this, the change in ligand binding affinities due to the top non-synonymous mutations are generally consistent with the reported literature, indicating host infectivity and drug resistance effects.

## Conclusion

In this study, we refined our c/µ test to identify the top mutations under adaptive selection in each coding and noncoding segment for the SARS-CoV-2 datasets from our previous report. Our c/µ approach identified 11 mutations in UTRs, our combined c/µ and Ka/Ks approach and Ks/µ identified the top 69 nonsynonymous and top 107 synonymous mutations in most proteins. The adaptive nonsynonymous mutations of key proteins and most other proteins were validated by the activity data from the literature, supporting that the c/µ test is more robust and should be used to extend the Ka/Ks test to detect molecular adaption. Additionally, the impact of several nonsynonymous mutations [M (I82T), Orf3a (S171L), Orf7a (T120I), Orf8 (Y73C), NSP2 (T85I), NSP3 (A890D, I412T, A488S K977Q, and S370L), NSP4 (V167L and A446V), NSP6 (T77A and V149A), NSP13 (A394V)] and the top synonymous mutations on SARS-CoV-2 fitness is unresolved, which should be investigated using both computational and experimental techniques. The impact of strong beneficial mutations on protein and nucleic acid function may be better explored using more accurate computational methods, such as molecular dynamics simulations. Furthermore, reverse genetics can help characterize point mutations on SARS-CoV-2 protein fitness and function.

## Supplementary Material

veae089_Supp

## Data Availability

The data underlying this article are available in the article. Supporting data are available as online supplementary material.
