# Supplementary material for: Enhanced detection and molecular modeling of adaptive mutations in SARS-CoV-2 coding and non-coding regions using the c/µ test
Source: Virus Evol. 2024 Nov 6;10(1):veae089. doi: 10.1093/ve/veae089 (PMC11584280; doi:10.1093/ve/veae089)
Supplement: veae089_Supp [file veae089_supp.zip › suppl_data/SI_v41_nocomment-notrack.docx]

**Table S1.** The comparison of the identified mutation count between our first paper and this current study.

| **Region** | **Top mutations**  **(1^st^ paper)**  **c/µ+Ka/Ks (SP)** | **Lit. I.D.**  **(1^st^ paper)**  **c/µ+Ka/Ks** | **Top mutations**  **(This study)**  **c/µ+Ka/Ks (NG)** | **Lit. I.D.**  **(This study)**  **c/µ+Ka/Ks** |
| --- | --- | --- | --- | --- |
| UTR | 54 | N/A | 11 | 2 |
| TR (Nonsyn) | 247 | N/A | 69 | 51 (70%) |
| TR (Syn) | N/A | N/A | 107 | 0% |

***Top mutations were identified using the criteria for UTR (c/µ>3), nonsynonymous sites in TR (c/µ>3 and Ka/Ks>2.5) and synonymous sites in TR (Ks/µ>3).**

****Ka/Ks methods: Single-Point Mutation Approximation (SP); Nei-Gojobori (NG).**

**1^st^ paper citation: Wu, C. et al. (2023), 'L-shaped distribution of the relative substitution rate (c/μ) observed for SARS-COV-2's genome, inconsistent with the selectionist theory, the neutral theory and the nearly neutral theory but a near-neutral balanced selection theory: Implication on “neutralist-selectionist” debate', Computers in Biology and Medicine, 153, 1-24.**

**N/A:** Not available.

**Table S2.** Identified top synonymous mutations under strong positive selection, weak positive selection and weak negative selection for S, M, Orf8, N, NSP1, NSP2, NSP3, NSP4, NSP5, NSP6, NSP9, NSP11, NSP13 and NSP14, their c/µ, Ks/µ, Ka/Ks and Ka/µ values and location within the predicted secondary RNA structure. See **Figures S8a-S8x** for the mutations mapped to their secondary RNA structures.

| **Seg** | **Topmut (AA)** | **NT position** | **c/µ** | **Ks/µ** | **Ka/Ks** | **Ka/µ** | **H-bonding?** | **W/in loop?** |
| --- | --- | --- | --- | --- | --- | --- | --- | --- |
| S | T723T | 2169 | 2.72 | 7.99 | 0.01 | 0.08 | Y | N |
| S | D294D | 882 | 0.86 | 7.72 | 0.00 | 0.00 | N | Y |
| S | F1062F | 3186 | 0.67 | 6.06 | 0.00 | 0.00 | Y | N |
| S | D111D | 333 | 0.51 | 4.35 | 0.01 | 0.03 | N | Y |
| S | H146H | 438 | 0.57 | 4.35 | 0.02 | 0.10 | N | Y |
| S | Y789Y | 2367 | 0.59 | 4.16 | 0.00 | 0.00 | N | Y |
| S | T1238T | 3714 | 1.22 | 3.66 | 0.00 | 0.00 | N | Y |
| S | N1178N | 3534 | 0.4 | 3.58 | 0.00 | 0.01 | N | Y |
| S | S929S | 2787 | 0.42 | 3.58 | 0.01 | 0.03 | Y | N |
| S | Q613Q | 1839 | 0.85 | 3.38 | 0.14 | 0.49 | Y | N |
| S | K1045K | 3135 | 0.46 | 3.28 | 0.02 | 0.06 | Y | N |
| S | Q321Q | 963 | 0.39 | 3.14 | 0.00 | 0.00 | Y | N |
| M | Y71Y | 213 | 2.95 | 20.66 | 0.00 | 0.00 | N | Y |
| M | F53F | 159 | 1.66 | 14.94 | 0.00 | 0.00 | Y | N |
| M | N41N | 123 | 0.51 | 4.52 | 0.00 | 0.01 | N | Y |
| M | L93L | 279 | 1.38 | 4.12 | 0.00 | 0.00 | N | Y |
| N | M1M | 3 | 0.18 | 216.37 | 0.00 | 0.14 | Y | N |
| N | R203R | 609 | 82.23 | 192.58 | 0.30 | 57.80 | Y | N |
| N | D3D | 9 | 50.58 | 55.85 | 0.89 | 49.65 | Y | N |
| N | F346F | 1038 | 1.22 | 11.02 | 0.00 | 0.00 | N | Y |
| N | D128D | 384 | 1.01 | 8.32 | 0.01 | 0.10 | N | Y |
| N | F110F | 330 | 0.60 | 5.40 | 0.00 | 0.00 | N | Y |
| N | F363F | 1089 | 0.50 | 4.41 | 0.00 | 0.01 | Y | N |
| N | A308A | 924 | 1.47 | 4.30 | 0.01 | 0.06 | N | Y |
| N | N192N | 576 | 0.48 | 4.19 | 0.00 | 0.02 | Y | N |
| N | F274F | 822 | 0.43 | 3.86 | 0.00 | 0.00 | N | Y |
| N | N228N | 684 | 0.37 | 3.31 | 0.00 | 0.01 | Y | N |
| Orf3a | W193W | 579 | 0.15 | 28.85 | 0.00 | 0.14 | Y | N |
| Orf3a | F43F | 129 | 0.60 | 4.96 | 0.01 | 0.06 | N | Y |
| Orf6 | D61D | 183 | 0.88 | 5.45 | 0.06 | 0.30 | N | Y |
| Orf8 | F120F | 360 | 7.06 | 55.88 | 0.02 | 0.88 | Y | N |
| Orf8 | H17H | 51 | 0.72 | 5.95 | 0.01 | 0.06 | N | Y |
| NSP1 | D156D | 468 | 4.14 | 37.20 | 0.00 | 0.01 | N | Y |
| NSP1 | V60V | 180 | 1.12 | 3.22 | 0.02 | 0.07 | N | Y |
| NSP1 | L16L | 48 | 1.02 | 3.00 | 0.01 | 0.03 | N | Y |
| NSP2 | S36S | 108 | 16.18 | 48.52 | 0.00 | 0.01 | Y | N |
| NSP2 | N435N | 1305 | 3.11 | 27.78 | 0.00 | 0.03 | N | Y |
| NSP2 | F231F | 693 | 0.64 | 5.79 | 0.00 | 0.00 | N | Y |
| NSP2 | G154G | 462 | 1.86 | 5.53 | 0.00 | 0.02 | Y | N |
| NSP2 | T629T | 1887 | 1.83 | 5.48 | 0.00 | 0.00 | Y | N |
| NSP2 | K618K | 1854 | 0.64 | 4.90 | 0.01 | 0.03 | N | Y |
| NSP2 | N8N | 24 | 0.46 | 4.08 | 0.00 | 0.01 | N | Y |
| NSP2 | N254N | 762 | 0.43 | 3.86 | 0.00 | 0.00 | Y | N |
| NSP2 | Y537Y | 1611 | 0.53 | 3.69 | 0.00 | 0.00 | Y | N |
| NSP3 | F106F | 318 | 67.87 | 610.78 | 0.00 | 0.00 | N | Y |
| NSP3 | F1089F | 3267 | 16.85 | 151.5 | 0.00 | 0.01 | N | Y |
| NSP3 | D10D | 30 | 4.10 | 36.65 | 0.00 | 0.03 | Y | N |
| NSP3 | V1298V | 3894 | 4.18 | 12.49 | 0.00 | 0.03 | Y | N |
| NSP3 | P1200P | 3600 | 4.08 | 11.70 | 0.02 | 0.27 | Y | N |
| NSP3 | D1075D | 3225 | 1.29 | 11.63 | 0.00 | 0.00 | Y | N |
| NSP3 | D1755D | 5265 | 0.93 | 8.38 | 0.00 | 0.00 | Y | N |
| NSP3 | T955T | 2865 | 2.20 | 6.60 | 0.00 | 0.01 | Y | N |
| NSP3 | H1274H | 3822 | 1.76 | 5.61 | 0.23 | 1.28 | N | Y |
| NSP3 | S1106S | 3318 | 0.67 | 5.57 | 0.01 | 0.06 | Y | N |
| NSP3 | F1107F | 3321 | 0.48 | 4.30 | 0.00 | 0.00 | N | Y |
| NSP3 | N1104N | 3312 | 0.48 | 4.19 | 0.00 | 0.02 | N | Y |
| NSP3 | I414I | 1242 | 0.94 | 4.08 | 0.01 | 0.05 | Y | N |
| NSP3 | Y246Y | 738 | 0.53 | 3.69 | 0.00 | 0.01 | N | Y |
| NSP3 | T1189T | 3567 | 1.98 | 3.59 | 0.33 | 1.17 | N | Y |
| NSP3 | N618N | 1854 | 0.37 | 3.31 | 0.00 | 0.01 | Y | N |
| NSP3 | D1031D | 3093 | 0.37 | 3.31 | 0.00 | 0.00 | Y | N |
| NSP3 | T608T | 1824 | 1.09 | 3.27 | 0.00 | 0.00 | N | Y |
| NSP3 | N931N | 2793 | 0.36 | 3.20 | 0.00 | 0.00 | N | Y |
| NSP4 | D144D | 432 | 7.66 | 68.84 | 0.00 | 0.01 | N | Y |
| NSP4 | S76S | 228 | 1.89 | 16.98 | 0.00 | 0.00 | N | Y |
| NSP4 | F337F | 1011 | 0.70 | 6.34 | 0.00 | 0.00 | N | Y |
| NSP4 | N131N | 393 | 0.40 | 3.53 | 0.00 | 0.01 | Y | N |
| NSP5 | C300C | 900 | 1.46 | 11.66 | 0.00 | 0.00 | N | N |
| NSP5 | E47E | 141 | 0.69 | 4.70 | 0.03 | 0.12 | N | Y |
| NSP5 | N231N | 693 | 0.47 | 4.24 | 0.00 | 0.00 | Y | N |
| NSP5 | N151N | 453 | 0.46 | 4.13 | 0.00 | 0.00 | Y | N |
| NSP6 | V120V | 360 | 7.63 | 22.82 | 0.00 | 0.04 | N | Y |
| NSP6 | Y175Y | 525 | 0.55 | 3.82 | 0.00 | 0.00 | Y | N |
| NSP9 | Y31Y | 93 | 4.15 | 29.02 | 0.00 | 0.00 | Y | N |
| NSP9 | Y87Y | 261 | 1.57 | 11.02 | 0.00 | 0.00 | N | Y |
| NSP9 | N95N | 285 | 0.66 | 5.90 | 0.00 | 0.01 | Y | N |
| NSP9 | L112L | 336 | 2.20 | 4.97 | 0.00 | 0.00 | Y | N |
| NSP9 | I65I | 195 | 1.11 | 3.14 | 0.17 | 0.54 | Y | N |
| NSP11 | H613H | 1839 | 17.01 | 151.26 | 0.00 | 0.23 | N | Y |
| NSP11 | M380M | 1140 | 0.03 | 72.12 | 0.00 | 0.03 | Y | N |
| NSP11 | T912T | 2736 | 16.87 | 50.62 | 0.00 | 0.00 | N | Y |
| NSP11 | P412P | 1236 | 16.85 | 50.47 | 0.00 | 0.04 | Y | N |
| NSP11 | D140D | 420 | 4.24 | 38.03 | 0.00 | 0.02 | Y | N |
| NSP11 | N552N | 1656 | 4.23 | 38.03 | 0.00 | 0.00 | N | Y |
| NSP11 | Y32Y | 96 | 2.69 | 18.86 | 0.00 | 0.00 | N | Y |
| NSP11 | N628N | 1884 | 1.41 | 12.73 | 0.00 | 0.00 | N | Y |
| NSP11 | Y455Y | 1365 | 1.71 | 11.92 | 0.00 | 0.01 | N | Y |
| NSP11 | D760D | 2280 | 0.70 | 6.28 | 0.00 | 0.00 | N | Y |
| NSP11 | N600N | 1800 | 0.52 | 4.69 | 0.00 | 0.00 | Y | N |
| NSP12 | Y217Y | 651 | 0.90 | 6.30 | 0.00 | 0.00 | N | Y |
| NSP12 | N503N | 1509 | 0.53 | 3.97 | 0.02 | 0.10 | Y | N |
| NSP12 | D113D | 339 | 0.43 | 3.86 | 0.00 | 0.01 | Y | N |
| NSP12 | D207D | 621 | 0.41 | 3.69 | 0.00 | 0.00 | N | Y |
| NSP12 | D542D | 1626 | 0.43 | 3.64 | 0.01 | 0.03 | Y | N |
| NSP12 | D578D | 1734 | 0.36 | 3.14 | 0.00 | 0.01 | N | Y |
| NSP13 | L280L | 840 | 4.45 | 10.09 | 0.00 | 0.00 | N | Y |
| NSP13 | D172D | 516 | 0.73 | 6.61 | 0.00 | 0.00 | N | Y |
| NSP13 | D126D | 378 | 0.43 | 3.91 | 0.00 | 0.00 | Y | N |
| NSP13 | D30D | 90 | 0.41 | 3.69 | 0.00 | 0.00 | N | Y |
| NSP13 | D375D | 1485 | 0.37 | 3.20 | 0.00 | 0.01 | N | Y |
| NSP13 | L495L | 1125 | 1.07 | 3.20 | 0.00 | 0.01 | N | Y |
| NSP14 | N73N | 219 | 2.28 | 20.45 | 0.00 | 0.01 | N | Y |
| NSP14 | L214L | 642 | 2.33 | 8.16 | 0.00 | 0.00 | Y | N |
| NSP14 | L216L | 648 | 1.97 | 6.87 | 0.00 | 0.01 | N | Y |
| NSP14 | F176F | 528 | 0.72 | 6.50 | 0.00 | 0.00 | N | Y |
| NSP15 | R216R | 648 | 3.46 | 4.48 | 0.66 | 2.96 | N | Y |
| NSP15 | A199A | 597 | 1.19 | 3.56 | 0.00 | 0.00 | Y | N |

*Top Mut (AA/NT) location is segment-specific.

**H-bonding/w/in loop (Y=yes/ N=no): If synonymous mutation occurs within hydrogen-bond base pairing interaction or in an RNA loop structure based on secondary structure prediction.

**Table S3.** Identified top non-synonymous mutations under strong positive selection, weak positive selection, weak negative selection and conserved sites in Envelope (E), Membrane (M), Orf3a, Orf7a, Orf8, NSP2, NSP3, NSP4, NSP6, NSP12 and NSP13, their position-based c/µ, Ka/Ks, Ka/µ and Ks/µ values and reported mutation effects from the literature. See **Figures S9a-S9x** for the mutations mapped to their protein structures.

| **Seg** | **Top mut** | **c/µ** | **Ka/Ks** | **Ka/µ** | **Ks/µ** | **Mutation effect** | **Ref** |
| --- | --- | --- | --- | --- | --- | --- | --- |
| **E** | **P71L** | **3.20** | **130.24** | **4.79** | **0.04** | Increased lethality; may stabilize protein structure | Mou et al. 2021 |
| **M** | **I82T** | **14.08** | **99.32** | **18.30** | **0.18** | N/A | N/A |
| **Orf3a** | **Q57H** | **13.68** | **Ks=0!** | **15.61** | **0.00** | Promotes evasion of cytokine, chemokine and IFN-stimulated gene expression in primary human respiratory cells | Wu et al. 2021b |
| **Orf3a** | **S26L** | **12.40** | **480.48** | **21.21** | **0.04** | Not functionally significant due to its presence amongst several sarbecoronaviruses | Zhang et al. 2022 |
| **Orf3a** | **S253P** | **4.23** | **48.86** | **6.28** | **0.13** | Associated with severe patient outcome | Cruz et al. 2022 |
| **Orf3a** | **S171L** | **3.53** | **141.47** | **6.11** | **0.04** | Reduced binding to Vps39 complex, reducing Orf3a lysosomal localization (negative fitness effect) | Cruz et al. 2022 |
| **Orf7a** | **V82A** | **11.23** | **457.98** | **16.83** | **0.04** | N/A | N/A |
| **Orf7a** | **T120I** | **10.77** | **Ks=0!** | **15.96** | **0.00** | May facilitate viral pathogenesis | Cruz et al. 2022 |
| **Orf8** | **R52I** | **16.92** | **902.47** | **22.44** | **0.02** | May facilitate viral pathogenesis | Cruz et al. 2022 |
| **Orf8** | **Y73C** | **16.72** | **Ks=0!** | **19.45** | **0.00** | N/A | N/A |
| **Orf8** | **E92K** | **4.54** | **105.72** | **5.18** | **0.05** | Induced weight loss and macrophage-induced lung inflammation in infected mice | McGrath et al. 2024 |
| **NSP2** | **T85I** | **8.95** | **31.74** | **13.10** | **0.41** | Predicted to enhance infectivity profile | Wang et al. 2021 |
| **NSP3** | **T183I** | **16.95** | **1303.79** | **24.93** | **0.02** | Predicted to decrease NSP3 stability | Saha et al. 2021 |
| **Nsp3** | **A890D** | **16.79** | **405.42** | **24.22** | **0.06** | N/A | N/A |
| **NSP3** | **I412T** | **16.29** | **Ks=0!** | **21.29** | **0.00** | N/A | N/A |
| **NSP3** | **P1469S** | **8.82** | **Ks=0!** | **13.23** | **0.00** | Predicted to decrease NSP3 stability | Saha et al. 2021 |
| **NSP3** | **P1228L** | **8.05** | **1350.24** | **12.18** | **0.01** | Predicted to decrease NSP3 stability | Saha et al. 2021 |
| **NSP3** | **A488S** | **7.69** | **313.24** | **11.51** | **0.04** | N/A | N/A |
| **NSP3** | **P822L** | **5.13** | **Ks=0!** | **7.70** | **0.00** | Predicted to stabilize NSP3 | Azzeri et al. 2024 |
| **NSP3** | **K977Q** | **4.17** | **97.16** | **4.76** | **0.05** | N/A | N/A |
| **NSP3** | **S370L** | **4.11** | **246.74** | **7.12** | **0.03** | N/A | N/A |
| **NSP4** | **T492I** | **11.49** | **74.64** | **16.90** | **0.23** | Predicted to modulate NSP cleavage and increase viral transmission and adaptability | Lin et al. 2023 |
| **NSP4** | **V167L** | **7.59** | **Ks=0!** | **11.35** | **0.00** | May increase transmissibility in vaccinated human patients | Brinkac et al. 2022 |
| **NSP4** | **A446V** | **5.14** | **419.50** | **7.71** | **0.02** | N/A | N/A |
| **NSP6** | **T77A** | **8.18** | **Ks=0!** | **12.27** | **0.00** | Predicted to destabilize NSP6 structure | Ghosh et al. 2022 |
| **NSP6** | **V149A** | **4.67** | **379.07** | **6.99** | **0.02** | Predicted to destabilize NSP6 structure | Ghosh et al. 2022 |
| **NSP6** | **L37F** | **3.27** | **Ks=0!** | **4.34** | **0.00** | Predicted to reduce protein stability and reduce interactions with host ER, reducing NSP6 function | Aiewsakun et al. 2021 |
| **NSP12** | **P77L** | **12.26** | **148.62** | **18.59** | **0.13** | Predicted to increase NSP12-TBK1 binding affinity and evade the immune response | Rashid et al. 2021 |
| **NSP12** | **E341D** | **4.25** | **Ks=0!** | **4.86** | **0.00** | Predicted to increase NSP12-TBK1 binding affinity and evade the immune response | Rashid et al. 2021 |
| **NSP13** | **A394V** | **7.61** | **620.98** | **11.41** | **0.02** | N/A | N/A |

***Ks=0! Indicates a technical error when calculating Ka/Ks for the AA site, but is still recorded under strong positive selection.**

**N/A: Not available.**

**References**

Aiewsakun, P., et al. (2021), 'SARS- CoV-2 genetic variations associated with COVID-19 pathogenicity', Microbial Genomics, 17 (12), 13.

Azzeri, A., et al. (2024), 'Unravelling the link between SARS-CoV-2 mutation frequencies, patient comorbidities, and structural dynamics', Plos One, 19 (3), 15.

Brinkac, L., et al. (2022), 'SARS-CoV-2 Delta variant isolates from vaccinated individuals', Bmc Genomics, 23 (1), 11.

Cruz, C. A. and Medina, P. M. (2022), 'Temporal changes in the accessory protein mutations of SARS-CoV-2 variants and their predicted structural and functional effects', Journal of Medical Virology, 94 (11), 5189-200.

Ghosh, N., Nandi, S., and Saha, I. (2022), 'Phylogenetic analysis of 17271 Indian SARS-CoV-2 genomes to identify temporal and spatial hotspot mutations', Plos One, 17 (3), 27.

Lin, X. Y., et al. (2023), 'The NSP4 T492I mutation increases SARS-CoV-2 infectivity by altering non-structural protein cleavage', Cell Host & Microbe, 31 (7), 1170-+.

McGrath, M. E., et al. (2024), 'SARS-CoV-2 ORF8 modulates lung inflammation and clinical disease progression', Plos Pathogens, 20 (5), 21.

Rashid, F., et al. (2021), 'Structural Analysis on the Severe Acute Respiratory Syndrome Coronavirus 2 Non-structural Protein 13 Mutants Revealed Altered Bonding Network With TANK Binding Kinase 1 to Evade Host Immune System', Frontiers in Microbiology, 12, 10.

Saha, I., et al. (2021), 'Hotspot Mutations in SARS-CoV-2', Frontiers in Genetics, 12, 16.

Wang, R., et al. (2021b), 'Analysis of SARS-CoV-2 mutations in the United States suggests presence of four substrains and novel variants', Communications Biology, 4 (1), 14.

Wu, S. Q., et al. (2021b), 'Effects of SARS-CoV-2 mutations on protein structures and intraviral protein-protein interactions', Journal of Medical Virology, 93 (4), 2132-40.

Zhang, J. T., et al. (2022), 'Understanding the Role of SARS-CoV-2 ORF3a in Viral Pathogenesis and COVID-19', Frontiers in Microbiology, 13, 19.

**Table S4.** ΔΔG_bind_ values approximated from docking analyses of ligands against the wild-type WT and mutant homology models of S, NSP11 and NSP5.

| **S** | | **Ligand** | **WT**  **ΔΔG_bind_** | **N501Y**  **ΔΔG_bind_** | **L452R**  **ΔΔG_bind_** | | **T478K**  **ΔΔG_bind_** | | **E484K**  **ΔΔG_bind_** | | **K417N**  **ΔΔG_bind_** | | **S477N**  **ΔΔG_bind_** | | **F490S**  **ΔΔG_bind_** | | **N439K**  **ΔΔG_bind_** | | **R346K**  **ΔΔG_bind_** | | **N440K**  **ΔΔG_bind_** | |
| --- | --- | --- | --- | --- | --- | --- | --- | --- | --- | --- | --- | --- | --- | --- | --- | --- | --- | --- | --- | --- | --- | --- |
|  |  | **hACE2** | **0.0** | -1.0 | -1.0 | | -0.1 | | -0.3 | | -1.1 | | -0.7 | | -1.3 | | -0.7 | | <-0.0 | | +0.2 | |
|  |  | **JMB2002 Fab** | **0.0** | -0.5 | +0.5 | | -0.3 | | -1.0 | | +0.2 | | -0.1 | | -1.4 | | -0.3 | | <-0.0 | | >+0.0 | |
| **NSP11** | | **Ligand** | **WT**  **ΔΔG_bind_** | **All muts**  **ΔΔG_bind_** | | **P323L**  **ΔΔG_bind_** | | **G671S**  **ΔΔG_bind_** | | **P227L**  **ΔΔG_bind_** | | **A97V**  **ΔΔG_bind_** | | **V776L**  **ΔΔG_bind_** | | **A185V**  **ΔΔG_bind_** | | **M666I**  **ΔΔG_bind_** | | **V792I**  **ΔΔG_bind_** | | **E802D**  **ΔΔG_bind_** |
|  | | **Remdesivir TP** | 0.0 | -0.7 | | -0.7 | | -1.4 | | +0.3 | | +0.4 | | -1.0 | | -0.1 | | +0.2 | | -0.8 | | -1.3 |
|  | | **Favipiravir TP** | 0.0 | +1.2 | | -1.0 | | +0.7 | | +0.5 | | +0.6 | | +1.2 | | +0.1 | | +0.8 | | +0.4 | | +1.2 |
|  | | **Ribavirin TP** | 0.0 | +1.2 | | +0.5 | | +0.5 | | +0.2 | | -0.2 | | +0.5 | | -0.4 | | +1.3 | | +0.3 | | +0.5 |
|  | | **Galidesivir TP** | 0.0 | +2.2 | | +0.7 | | +1.3 | | -0.5 | | +0.2 | | +1.3 | | +1.0 | | -0.6 | | +1.5 | | +0.8 |
|  | | **Sofosbuvir TP** | 0.0 | +1.3 | | +1.0 | | +2.2 | | +0.7 | | +0.1 | | +0.6 | | +1.4 | | +0.0 | | +2.2 | | +1.5 |
|  | | **Dasabuvir** | 0.0 | +1.8 | | +0.8 | | -0.2 | | +0.3 | | +0.7 | | 0.0 | | +0.7 | | -0.1 | | -0.1 | | +1.0 |
| **NSP5** | | **Ligand** | **WT**  **ΔΔG_bind_** | **All muts**  **ΔΔG_bind_** | | **G15S**  **ΔΔG_bind_** | | **K90R**  **ΔΔG_bind_** | | **L89F**  **ΔΔG_bind_** | | **T21I**  **ΔΔG_bind_** | |  |  |  |  |  |  |  |  |  |
|  |  | **Nirmatrelvir** | 0.0 | +0.2 | | +0.5 | | +0.1 | | +0.6 | | 0.0 | |  |  |  |  |  |  |  |  |  |
|  |  | **GC376** | 0.0 | -0.5 | | -0.5 | | -0.9 | | +0.1 | | -0.5 | |  |  |  |  |  |  |  |  |  |
|  |  | **Lufotrelvir** | 0.0 | +0.1 | | -0.2 | | -0.5 | | -0.9 | | -0.2 | |  |  |  |  |  |  |  |  |  |
|  |  | **Ensitrelvir** | 0.0 | -0.5 | | 0.0 | | 0.0 | | +0.9 | | +0.6 | |  |  |  |  |  |  |  |  |  |

**ΔΔG_bind_ = ΔG_bind, MUT_ – ΔG_bind, WT_. See Table S3 for the raw ΔG_bind_ scores of NSP11 and NSP5.**

**ΔΔG_bind_ = kcal/mol**

**Table S5.** ΔG_bind_ values approximated from docking analyses of ligands against the wild-type WT and mutant homology models of NSP11 and NSP5.

| **NSP11** | | **Ligand** | **WT**  **ΔG_bind_** | **All muts**  **ΔG_bind_** | **P323L**  **ΔG_bind_** | **G671S**  **ΔG_bind_** | **P227L**  **ΔG_bind_** | **A97V**  **ΔG_bind_** | **V776L**  **ΔG_bind_** | **A185V**  **ΔG_bind_** | **M666I**  **ΔG_bind_** | **V792I**  **ΔG_bind_** | **E802D**  **ΔG_bind_** |
| --- | --- | --- | --- | --- | --- | --- | --- | --- | --- | --- | --- | --- | --- |
|  | | **Remdesivir TP** | -10.2 | -10.9 | -11.0 | -11.7 | -9.9 | -9.8 | -11.3 | -10.4 | -10.0 | -11.0 | -11.6 |
|  | | **Favipiravir TP** | -9.9 | -8.7 | -11.0 | -9.3 | -9.4 | -9.4 | -8.8 | -9.9 | -9.2 | -9.6 | -8.8 |
|  | | **Ribavirin TP** | -9.7 | -8.5 | -9.3 | -9.3 | -9.5 | -9.9 | -9.2 | -10.2 | -8.5 | -9.4 | -9.3 |
|  | | **Galidesivir TP** | -10.4 | -8.2 | -9.7 | -9.1 | -11.0 | -10.3 | -9.1 | -9.4 | -11.1 | -9.0 | -9.6 |
|  | | **Sofosbuvir TP** | -9.6 | -8.3 | -8.6 | -7.4 | -8.9 | -9.4 | -9.0 | -8.2 | -9.6 | -7.3 | -8.1 |
|  | | **Dasabuvir** | -6.8 | -5.0 | -6.0 | -6.9 | -6.5 | -6.1 | -6.8 | -6.0 | -6.9 | -6.9 | -5.8 |
| **NSP5** | | **Ligand** | **WT**  **ΔG_bind_** | **All muts**  **ΔG_bind_** | **G15S**  **ΔG_bind_** | **K90R**  **ΔG_bind_** | **L89F**  **ΔG_bind_** | **T21I**  **ΔG_bind_** |  |  |  |  |  |
|  |  | **Nirmatrelvir** | -9.5 | -9.3 | -9.0 | -9.3 | -8.9 | -9.5 |  |  |  |  |  |
|  |  | **GC376** | -6.6 | -7.0 | -7.1 | -7.5 | -6.5 | -7.0 |  |  |  |  |  |
|  |  | **Lufotrelvir** | -6.1 | -6.0 | -6.2 | -6.5 | -7.0 | -6.2 |  |  |  |  |  |
|  |  | **Ensitrelvir** | -6.0 | -6.5 | -6.0 | -6.0 | -5.1 | -5.5 |  |  |  |  |  |

*** ΔG_bind_ = kcal/mol**

| 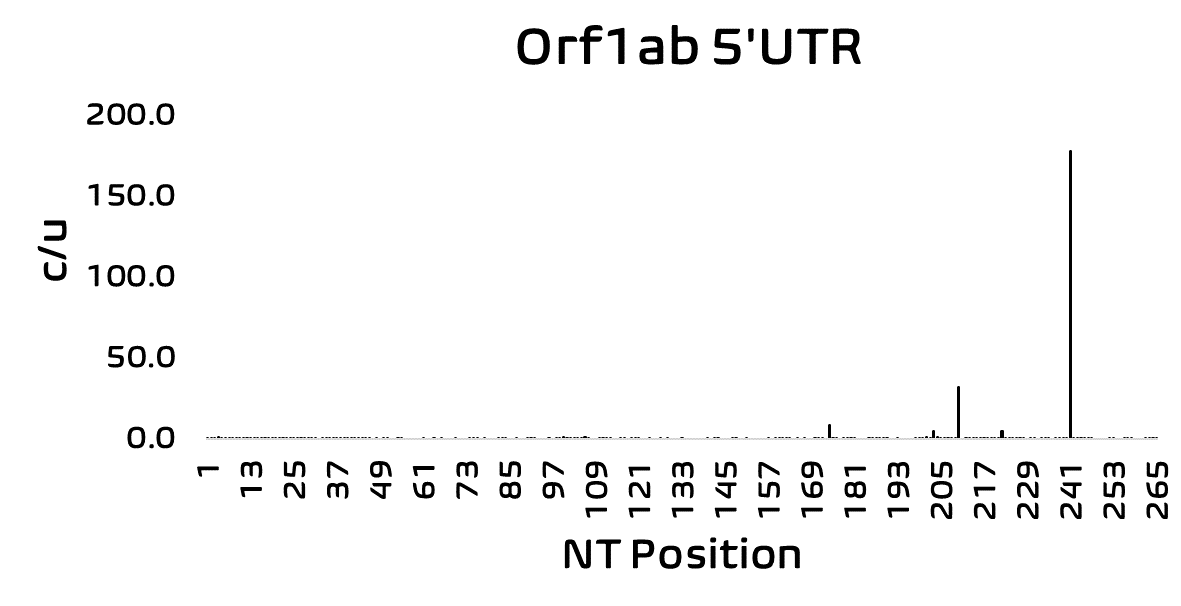 | 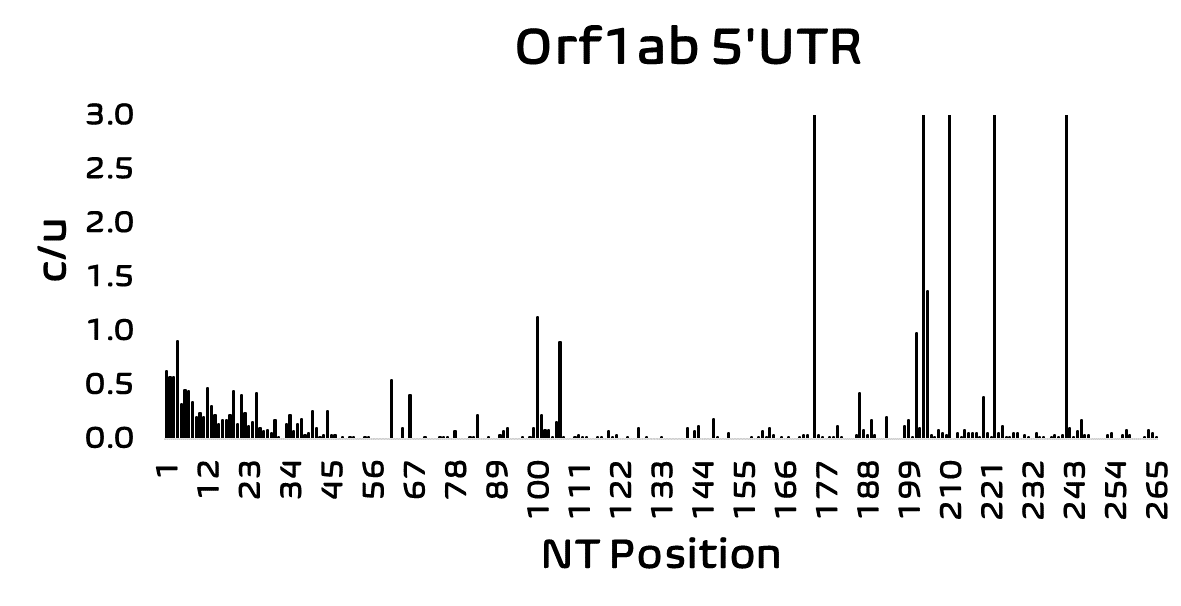 |
| --- | --- |
| 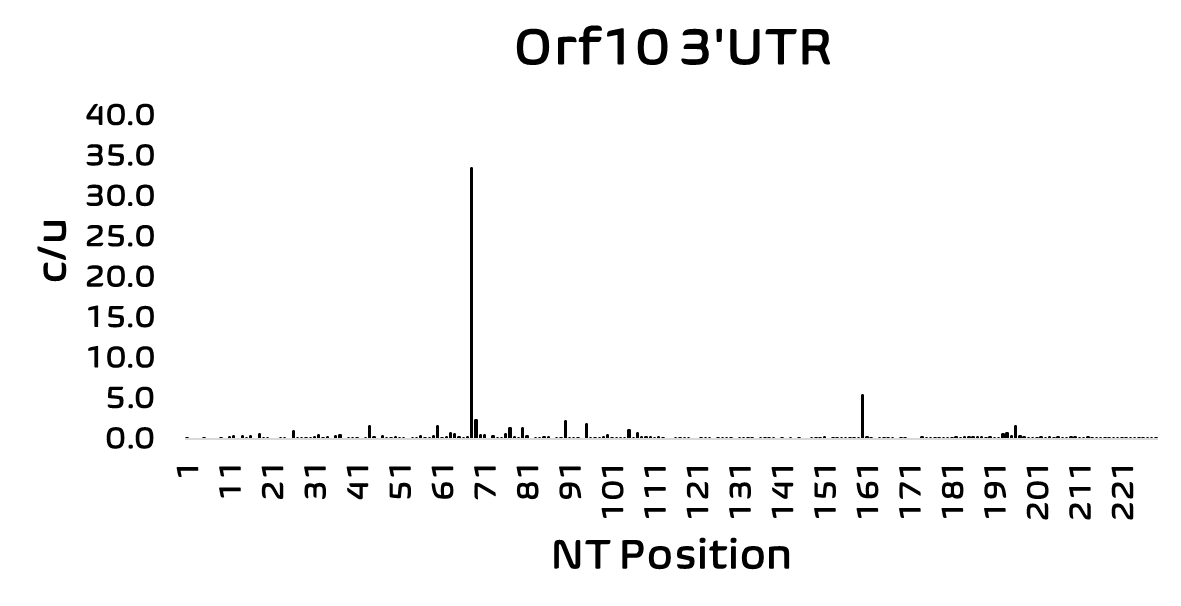 | 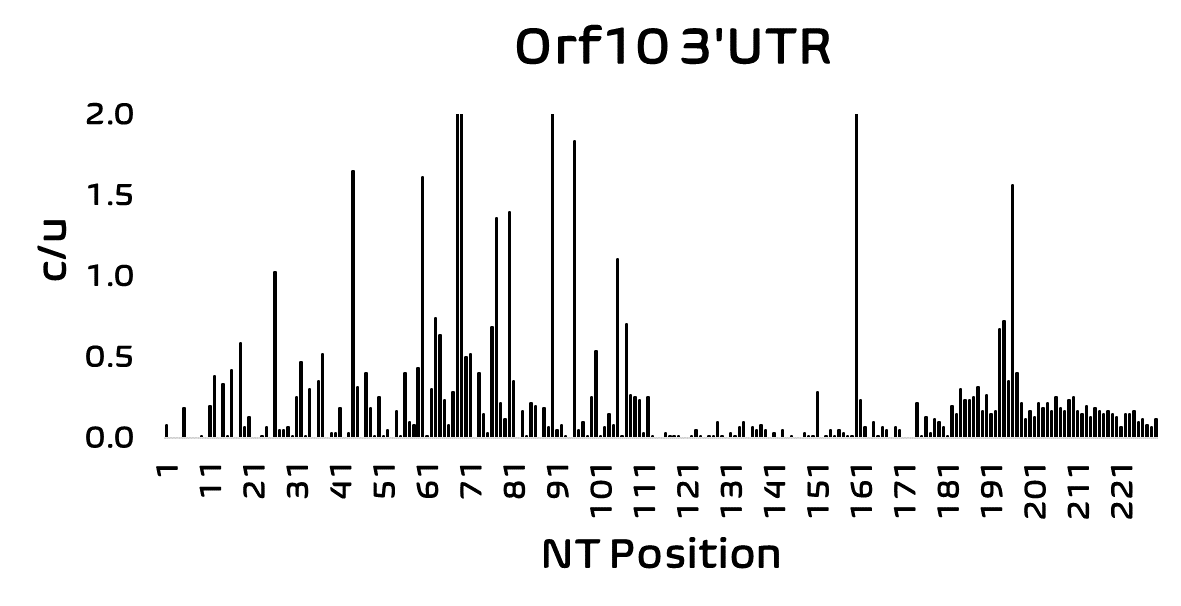 |

**Figure S1.** Position-based c/µ values for Orf1ab 5’UTR and Orf10 3’UTR.

| 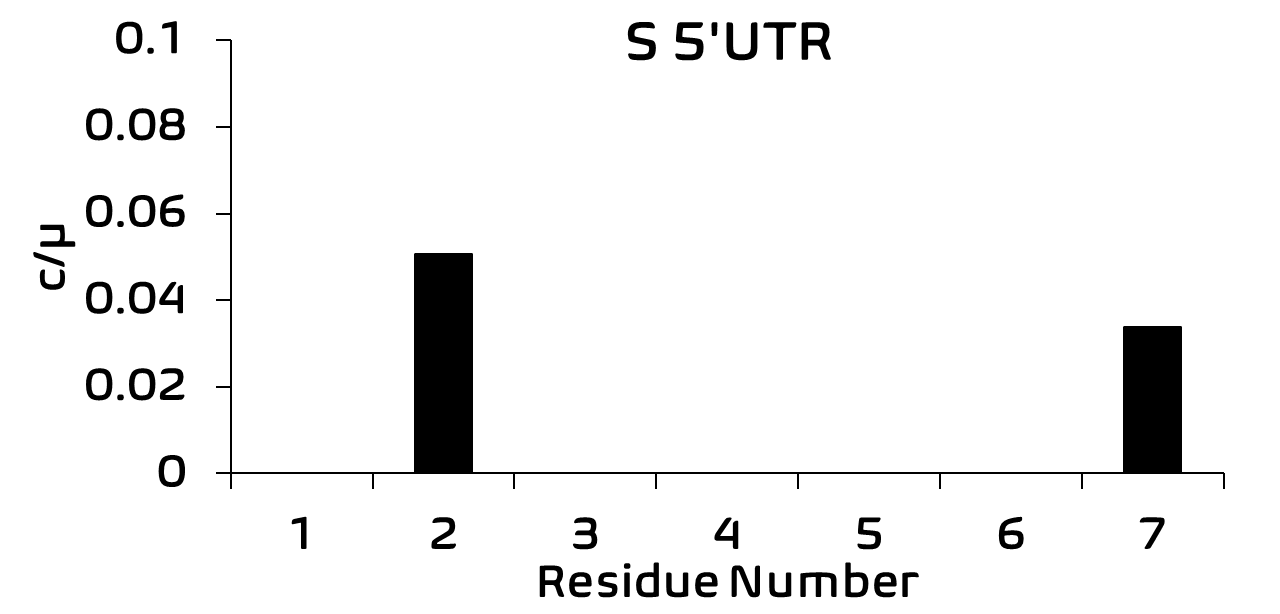 | 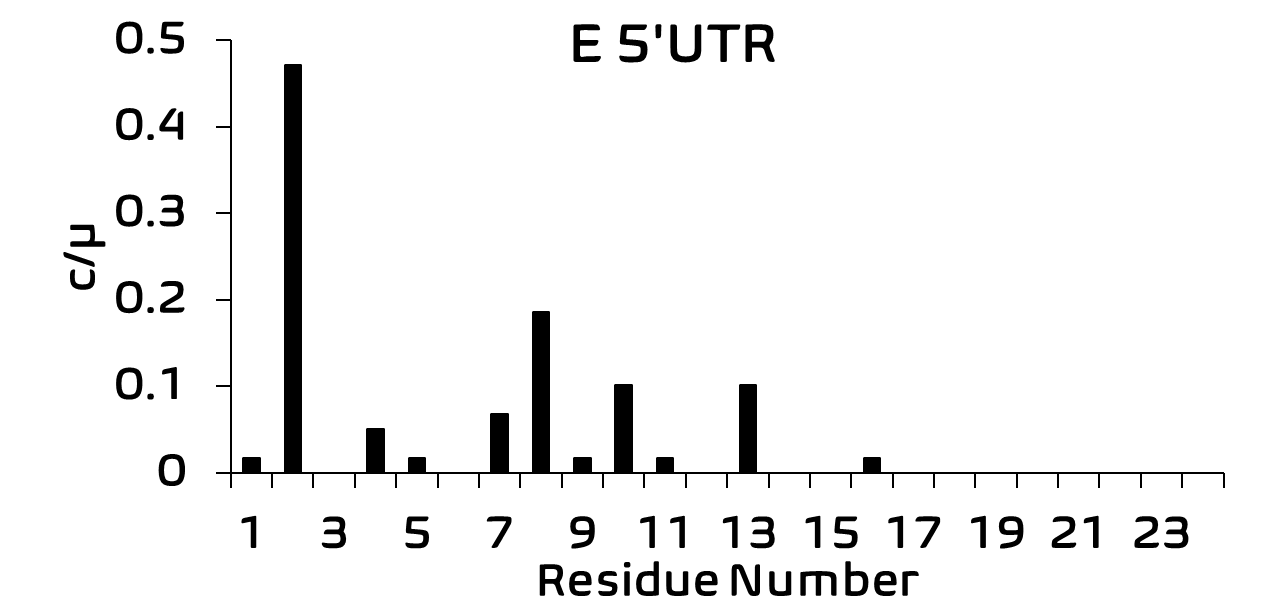 | 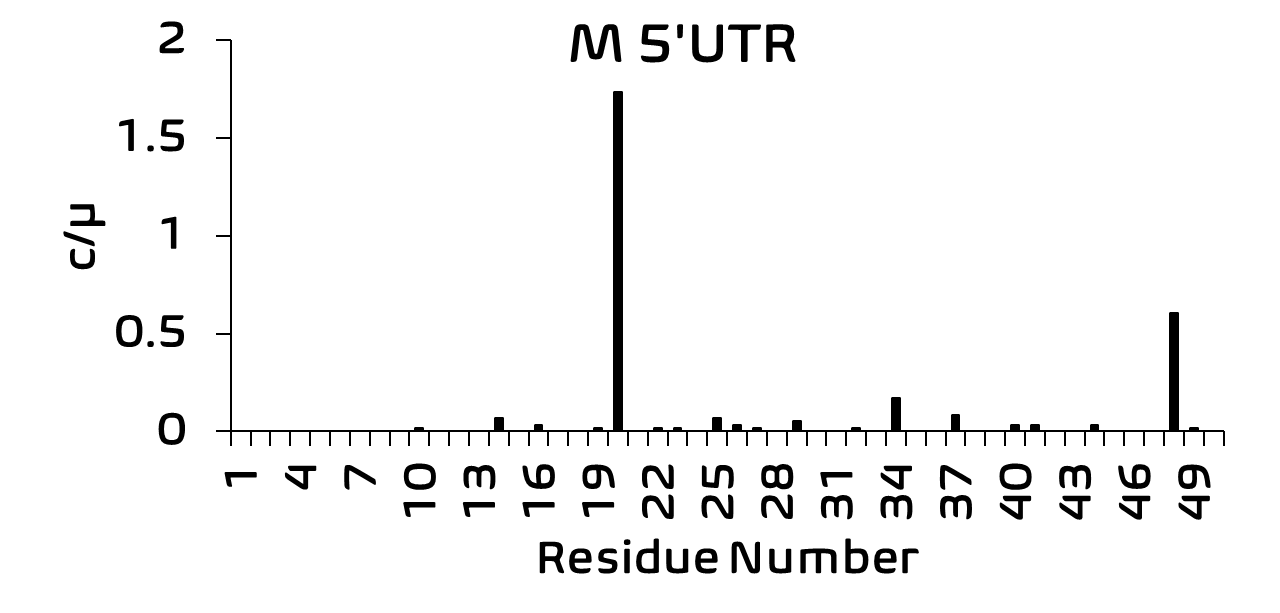 |
| --- | --- | --- |
| 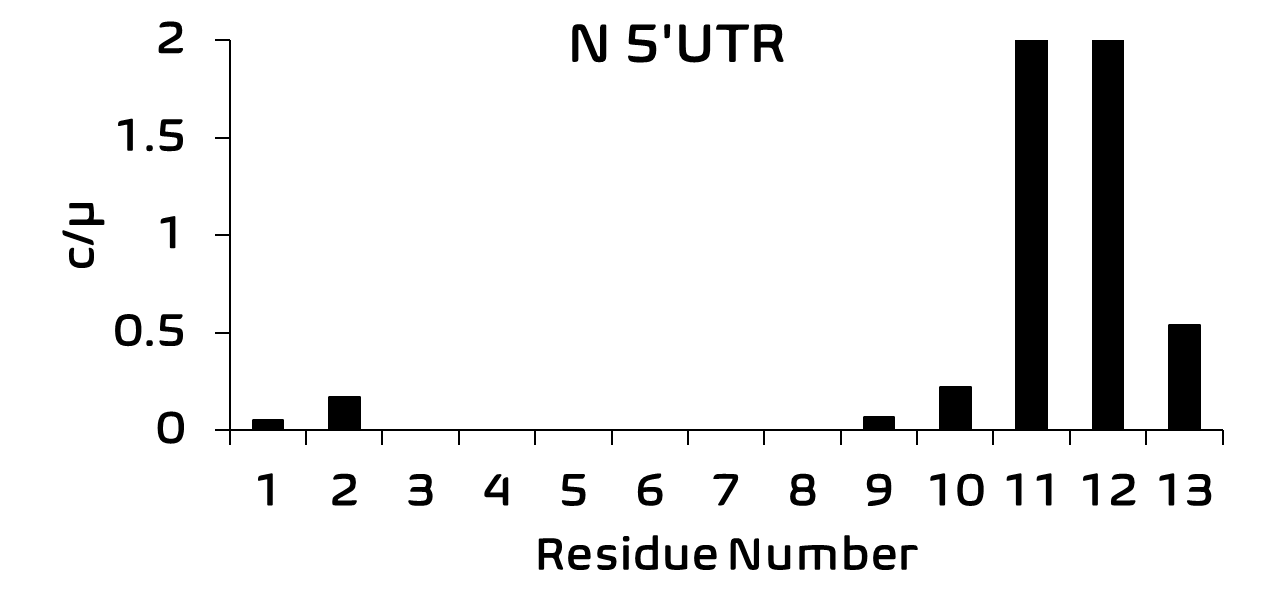 | 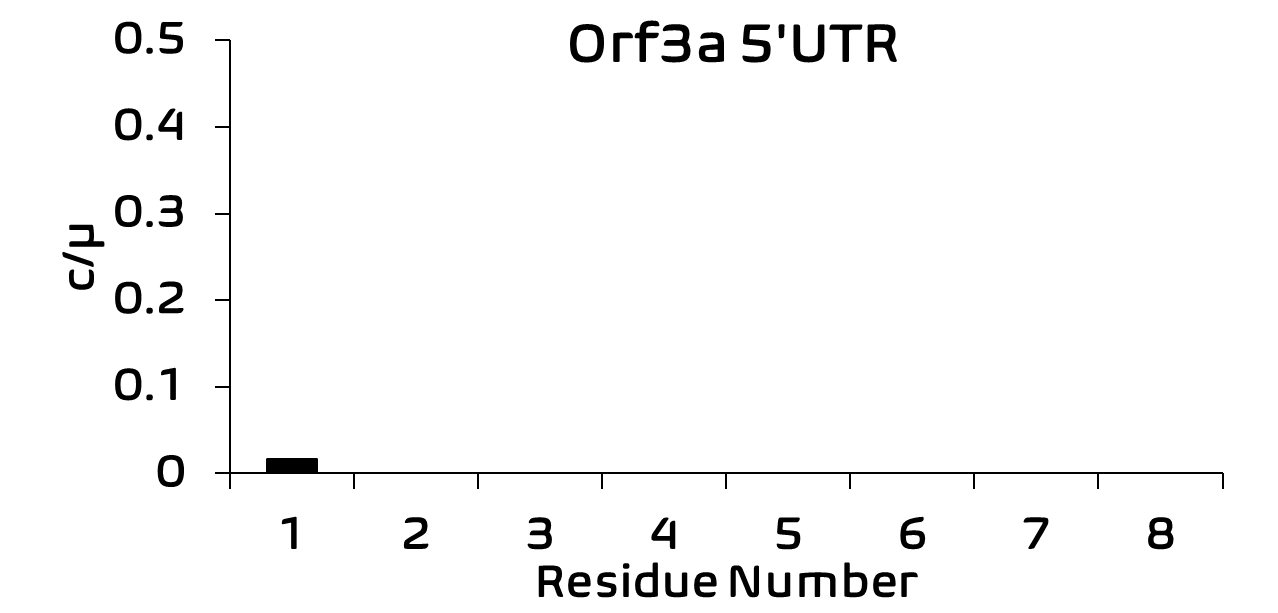 | 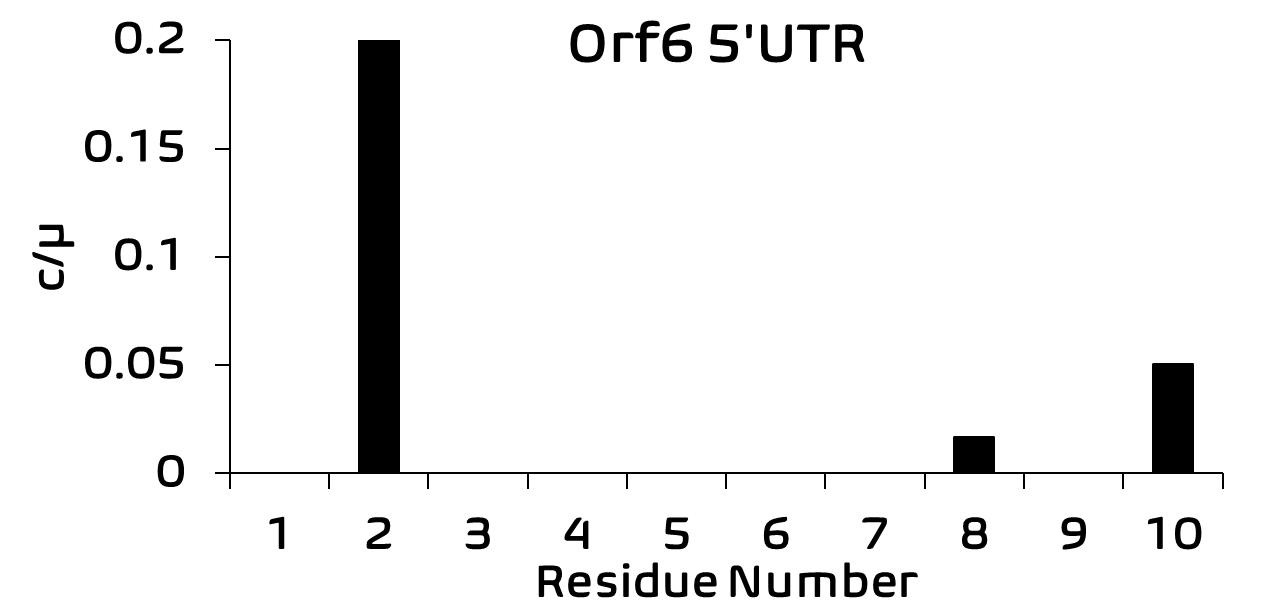 |
| 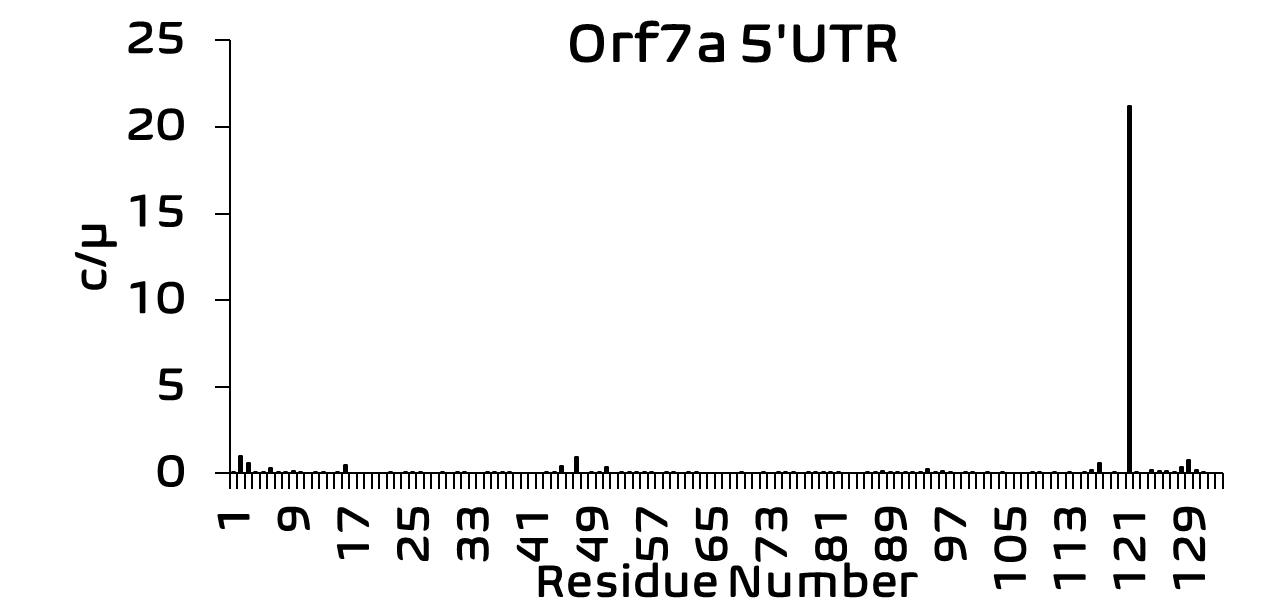 | 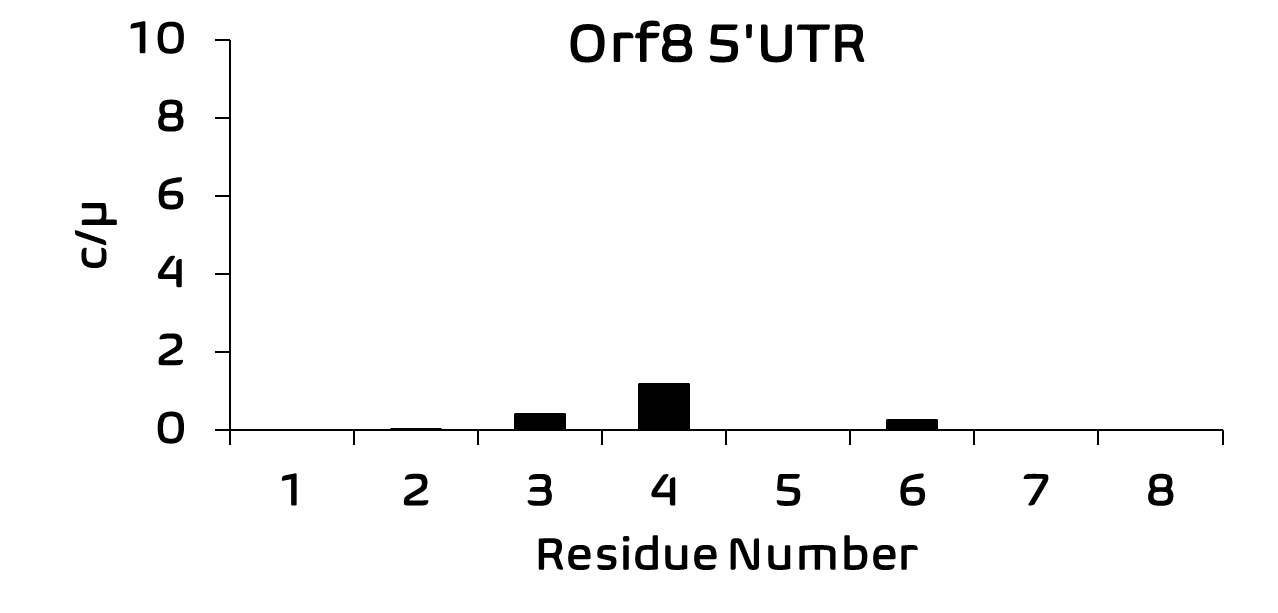 |  |

**Figure S2.** Position-based c/µ values for each UTR.

| 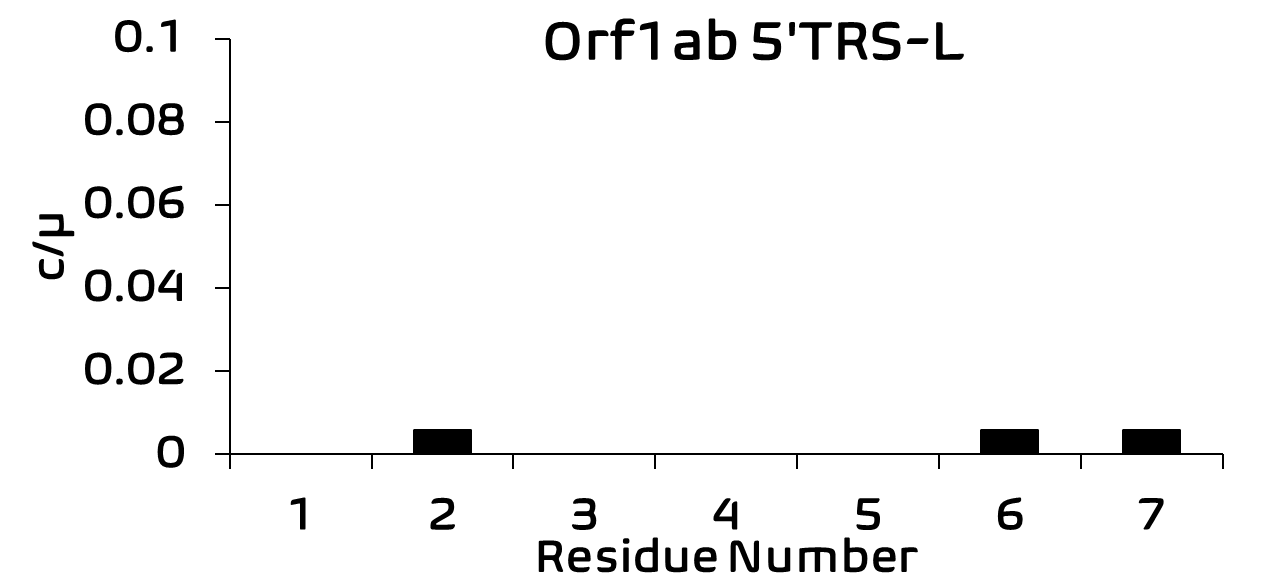 | 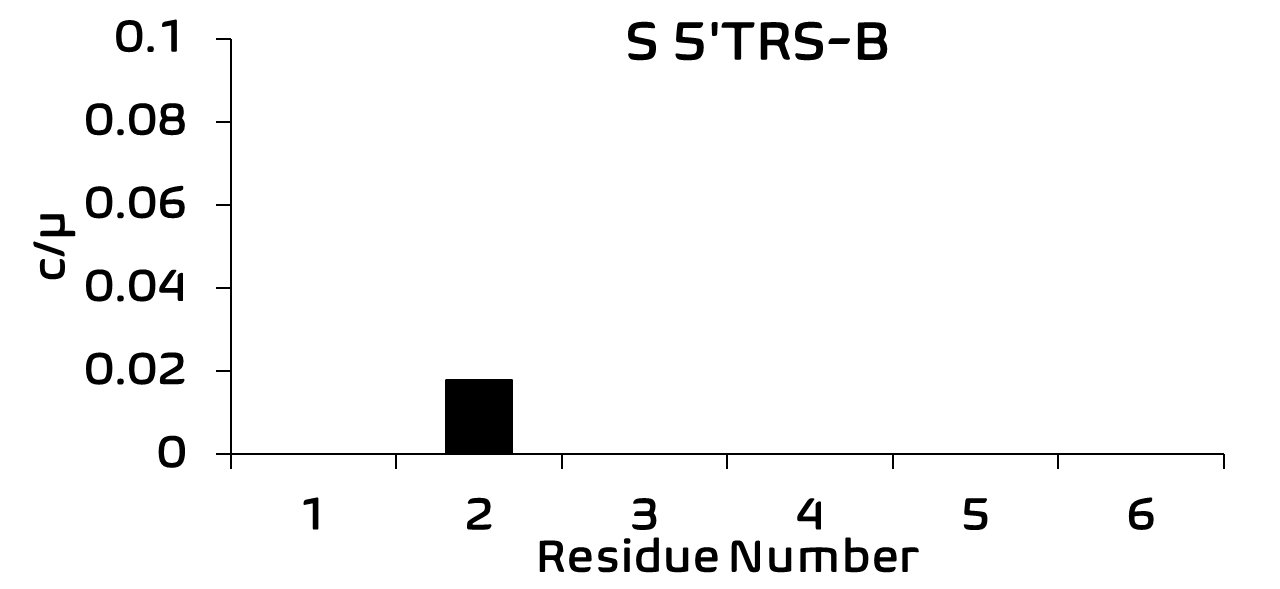 | 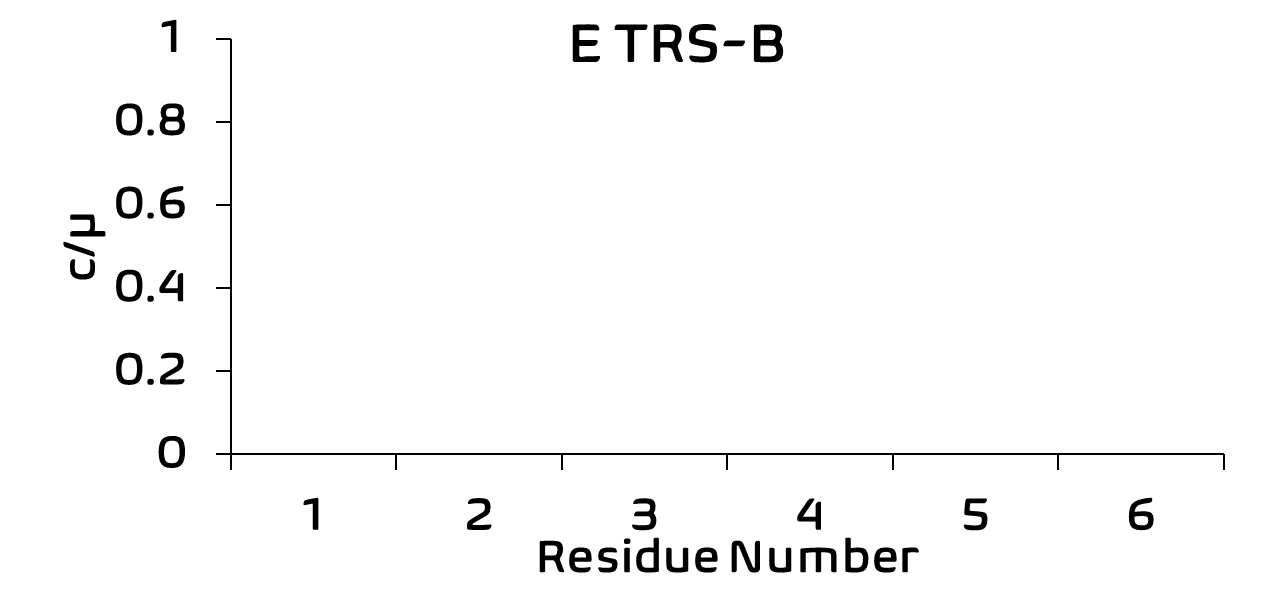 |
| --- | --- | --- |
| 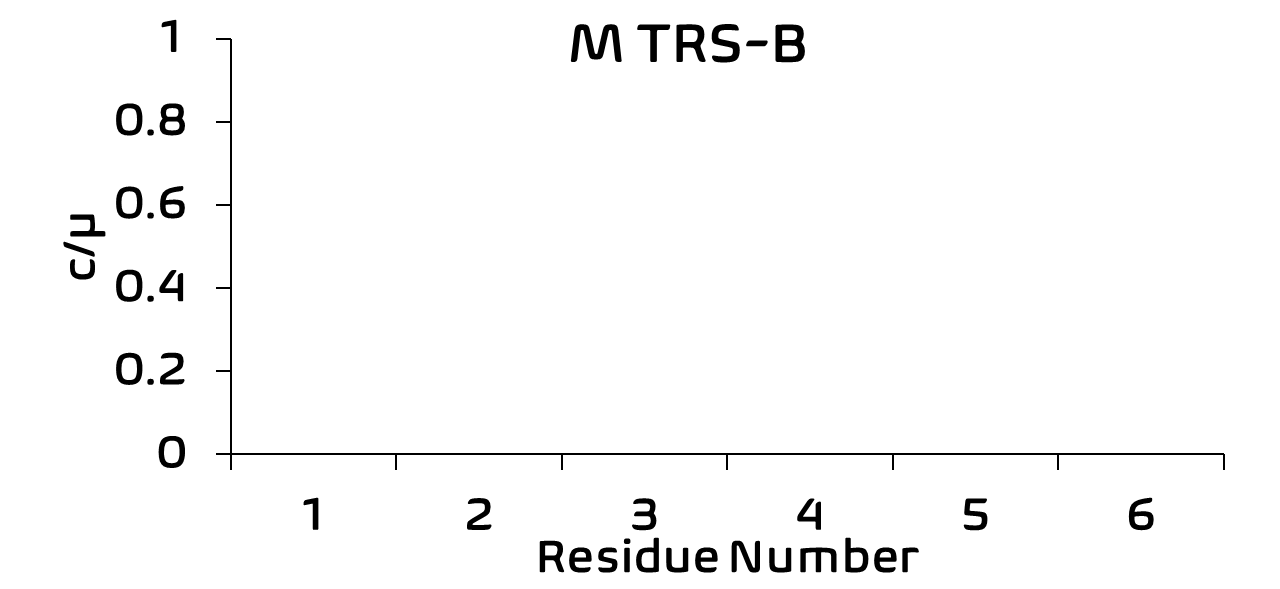 | 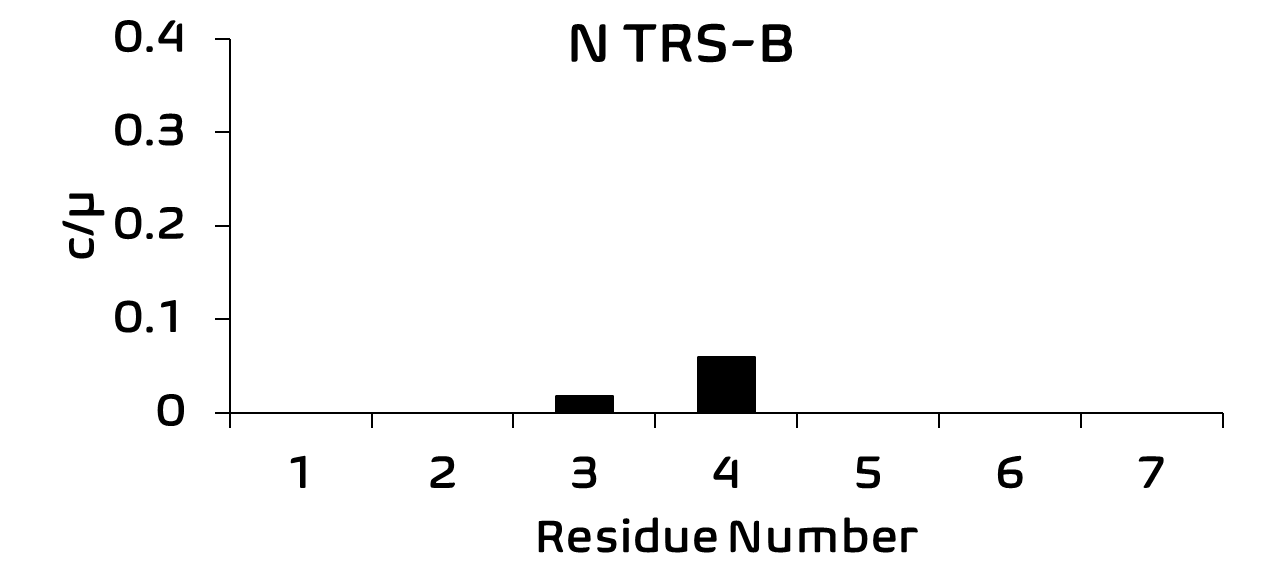 | 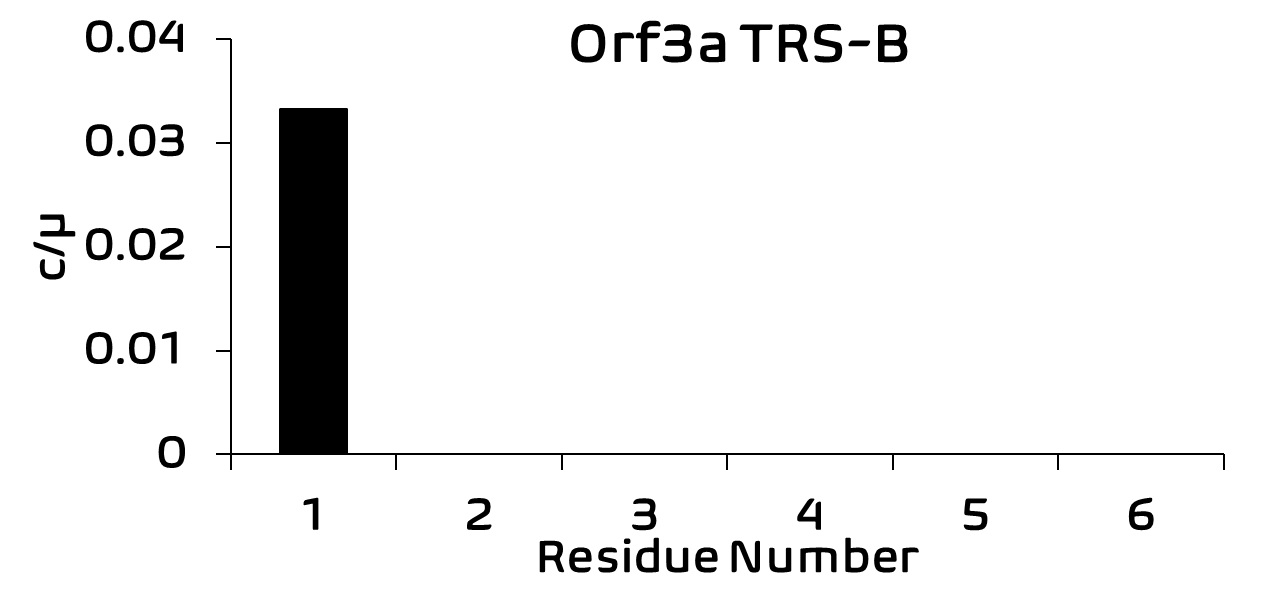 |
| 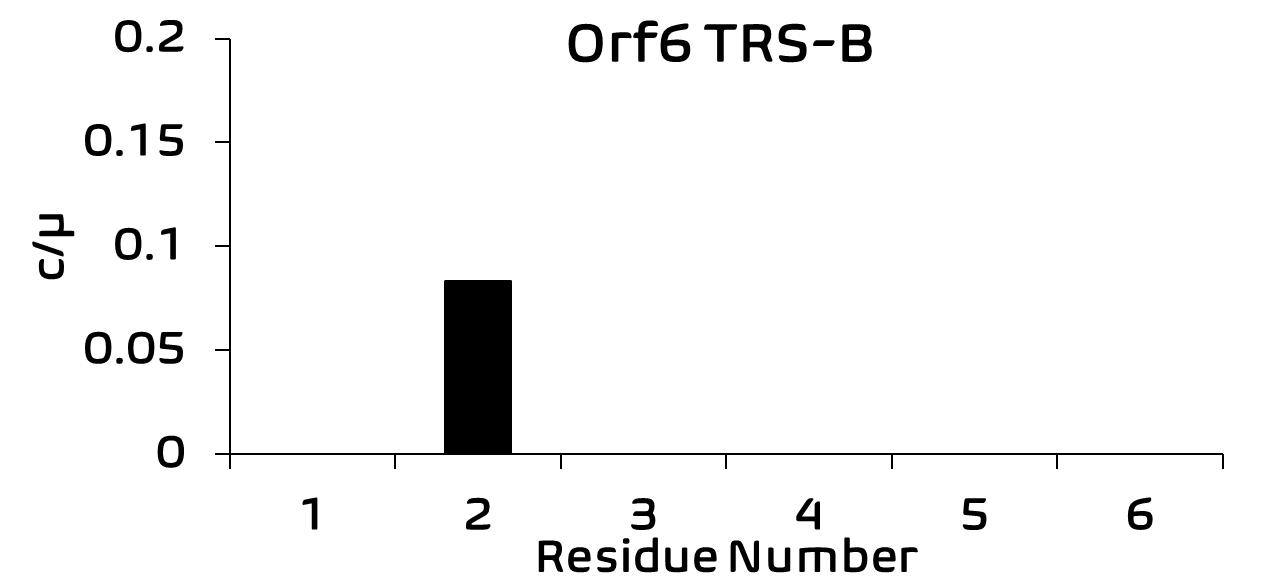 | 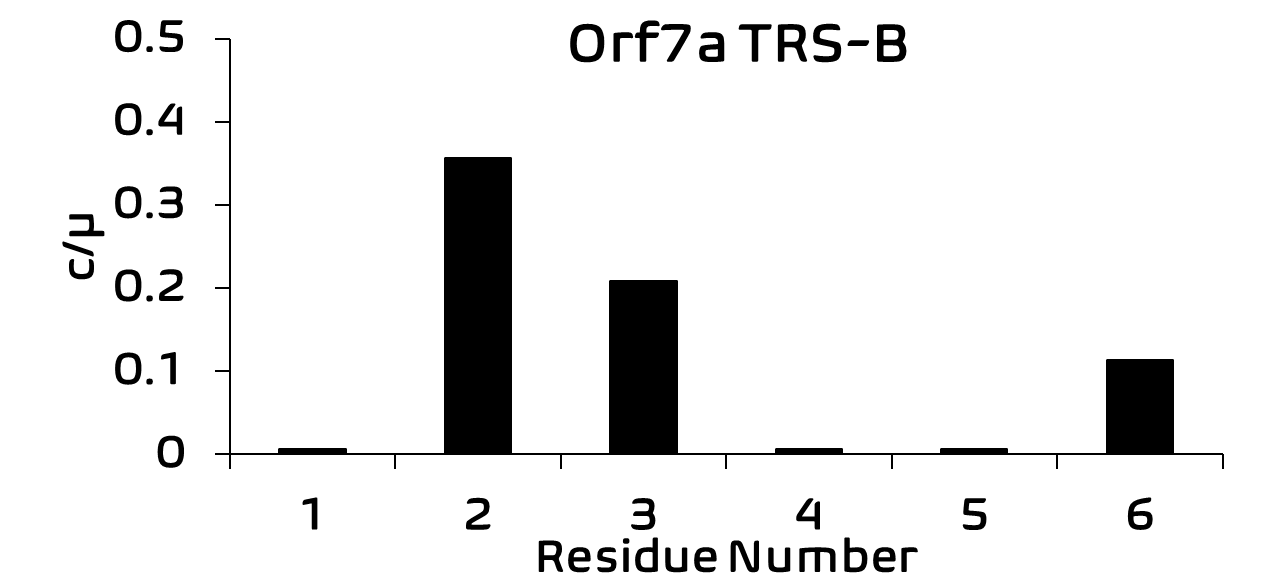 | 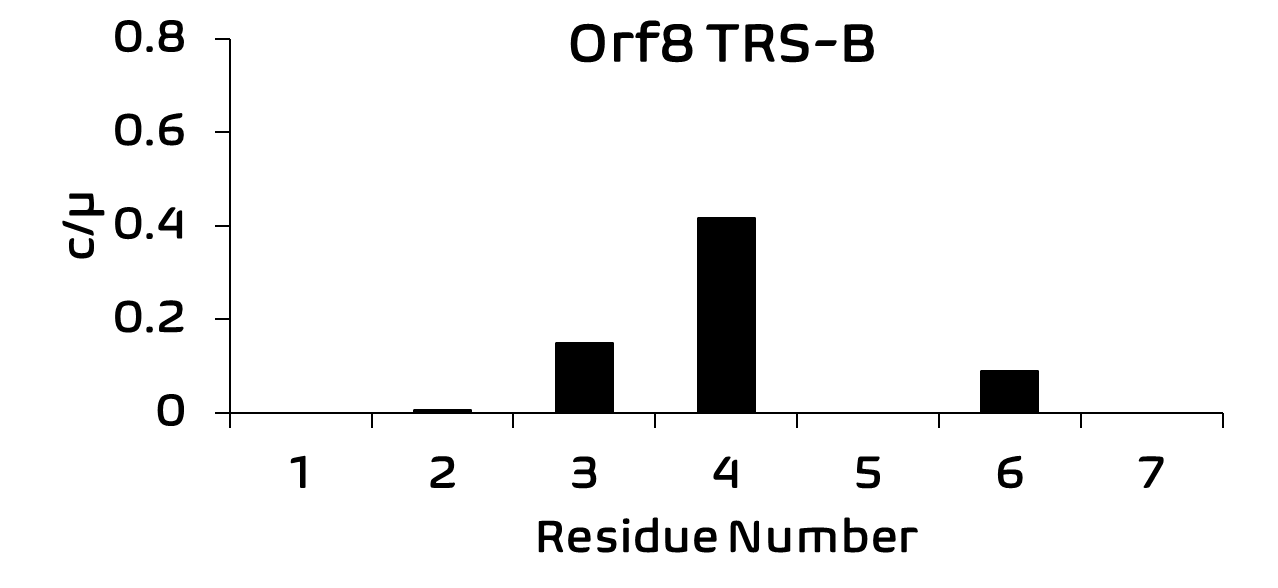 |

**Figure S3.** Position-based c/µ values for each TRS.

| 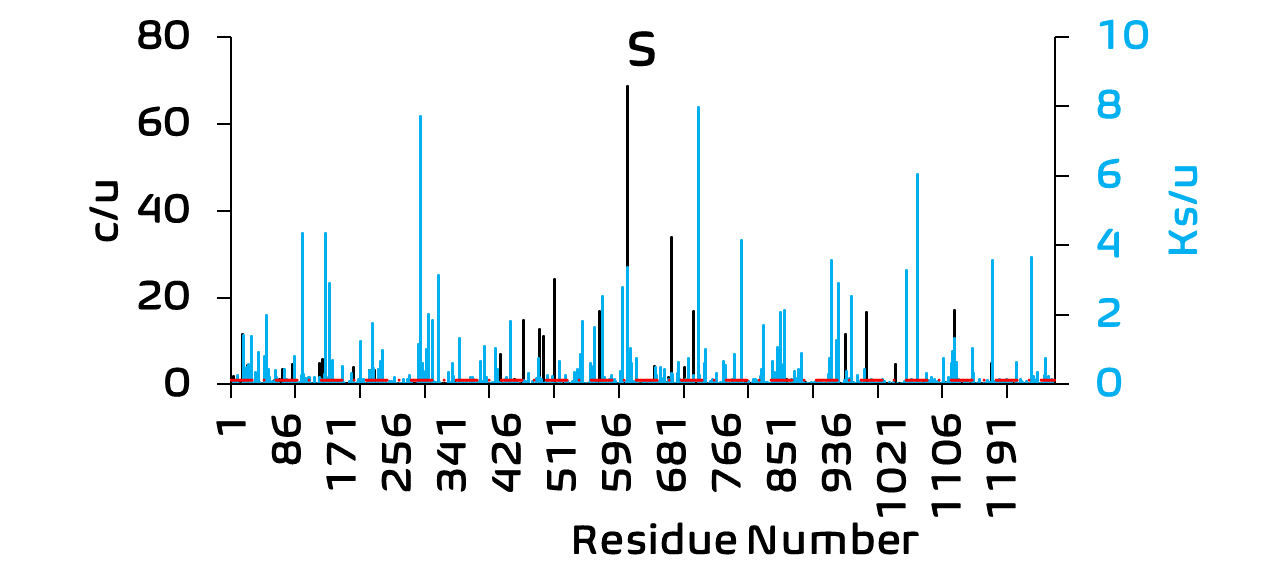 | 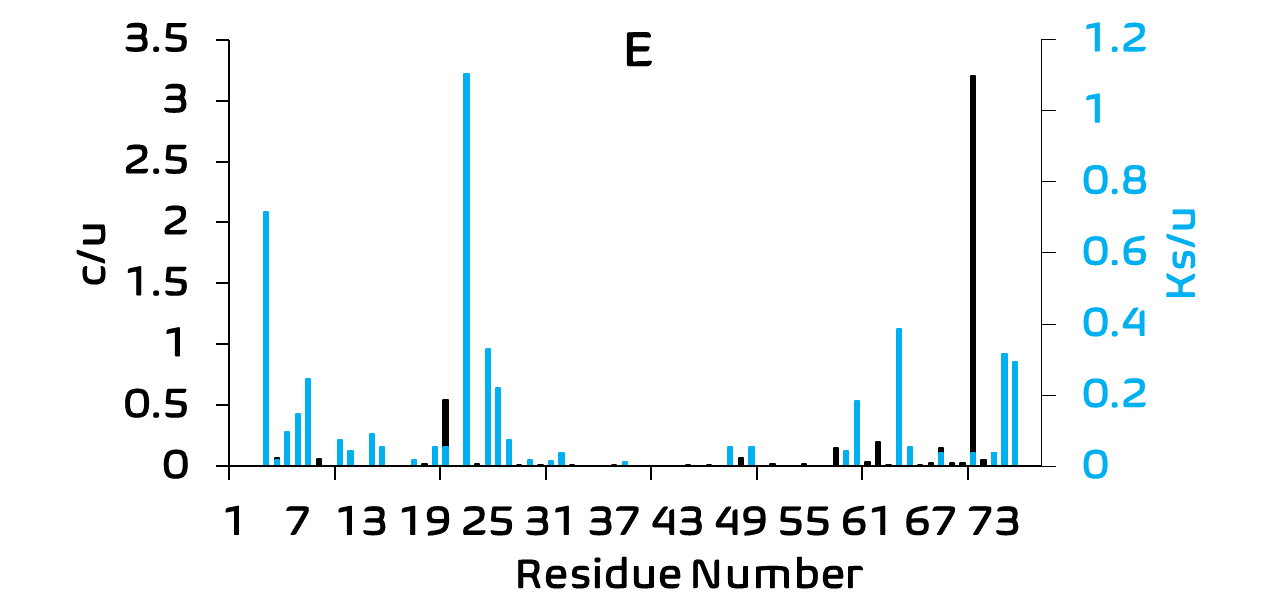 | 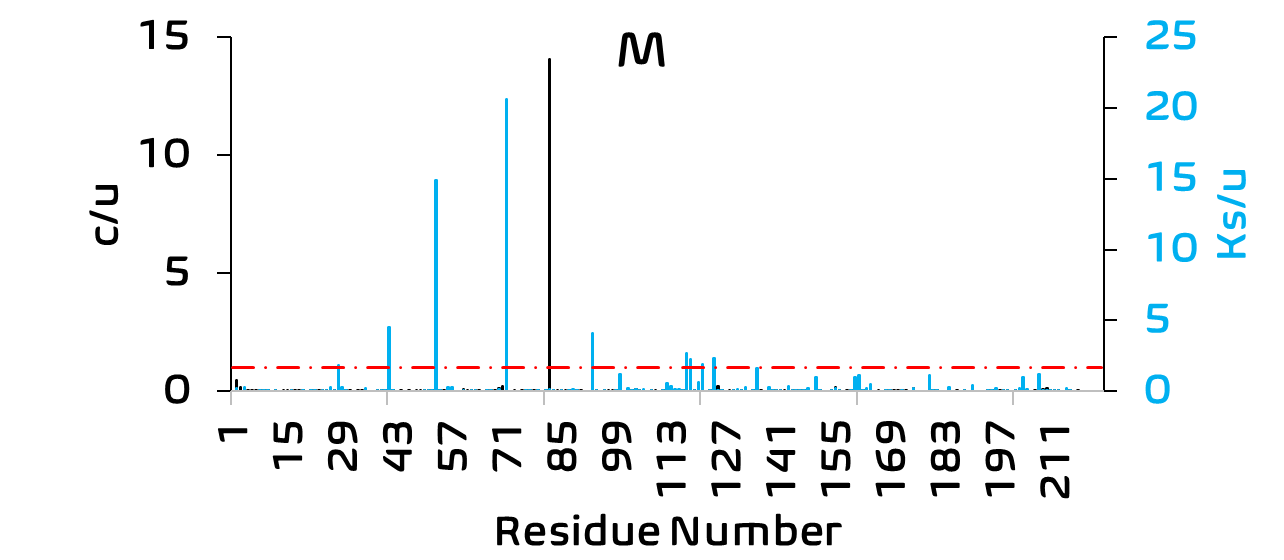 |
| --- | --- | --- |
| 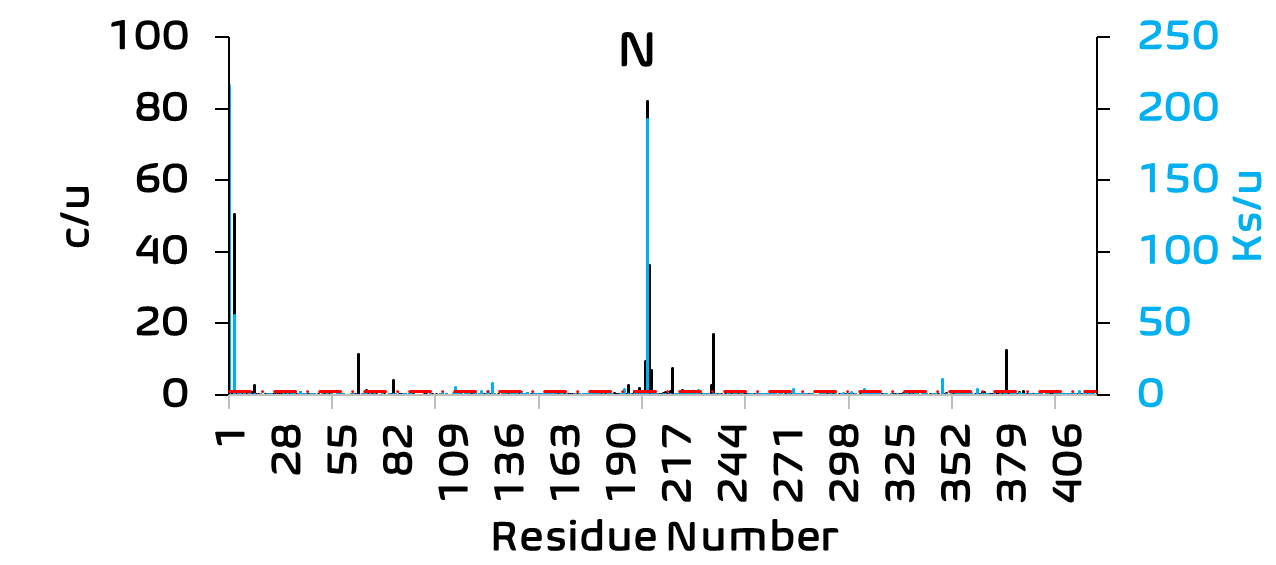 | 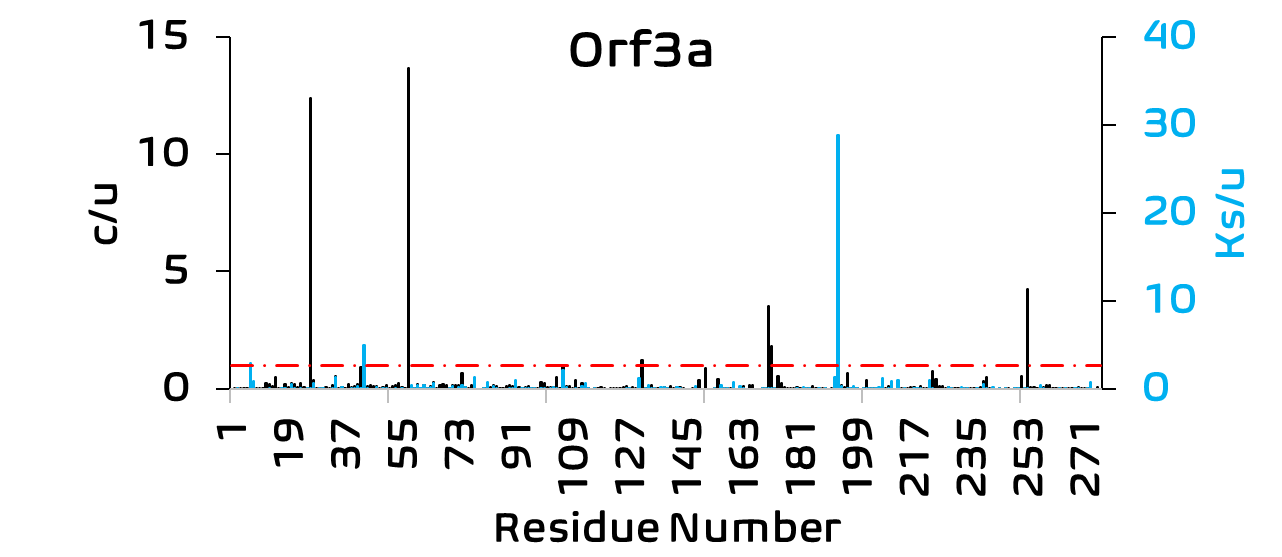 | 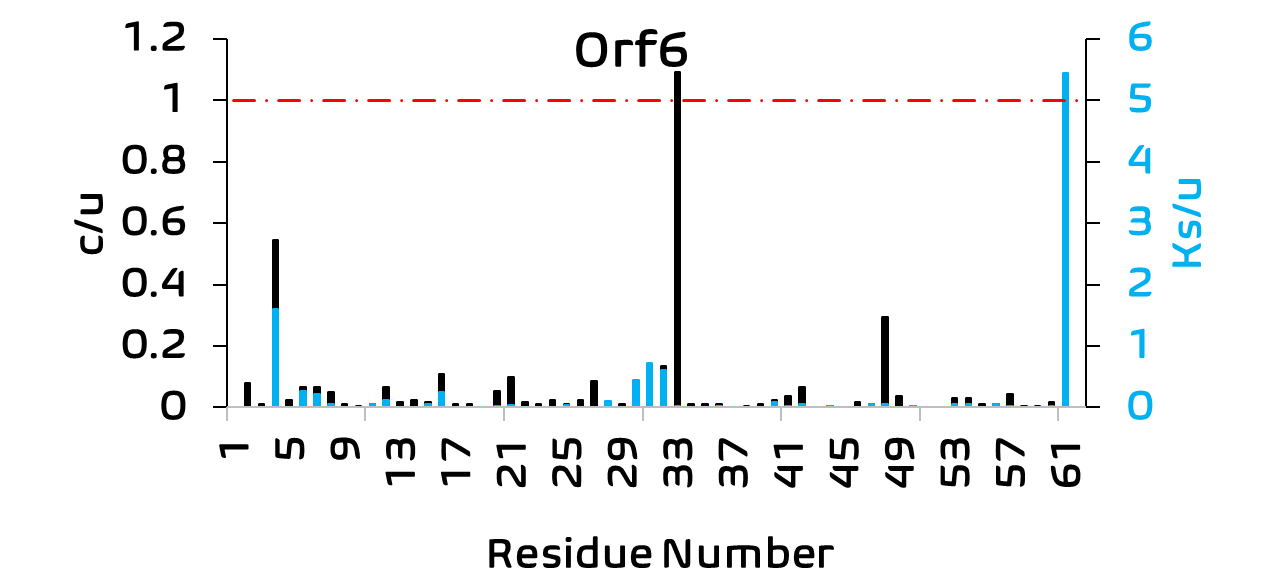 |
| 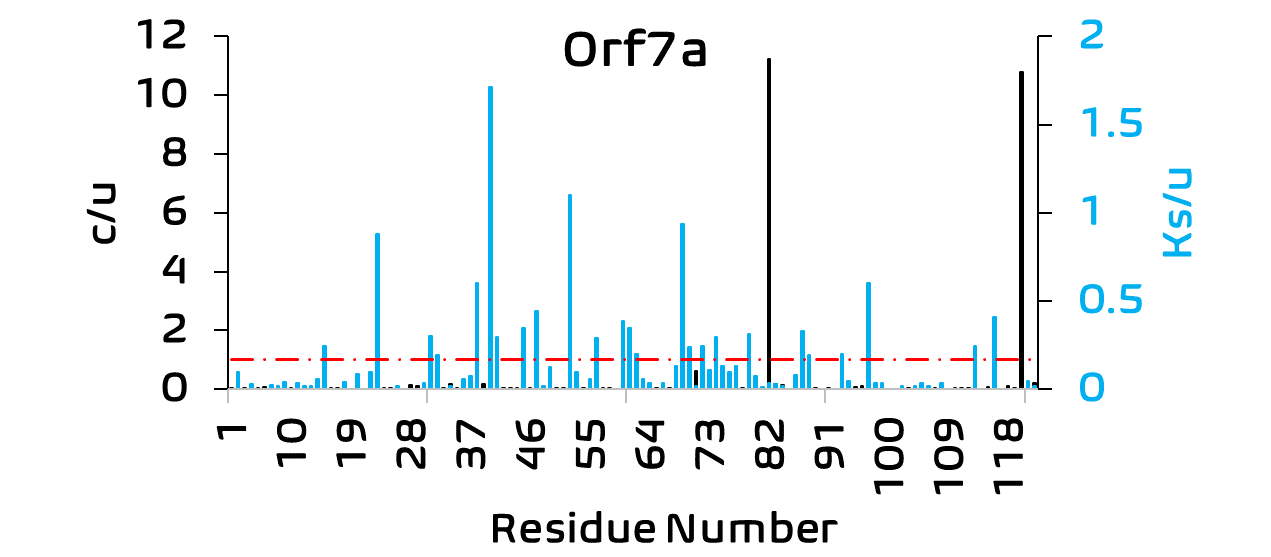 | 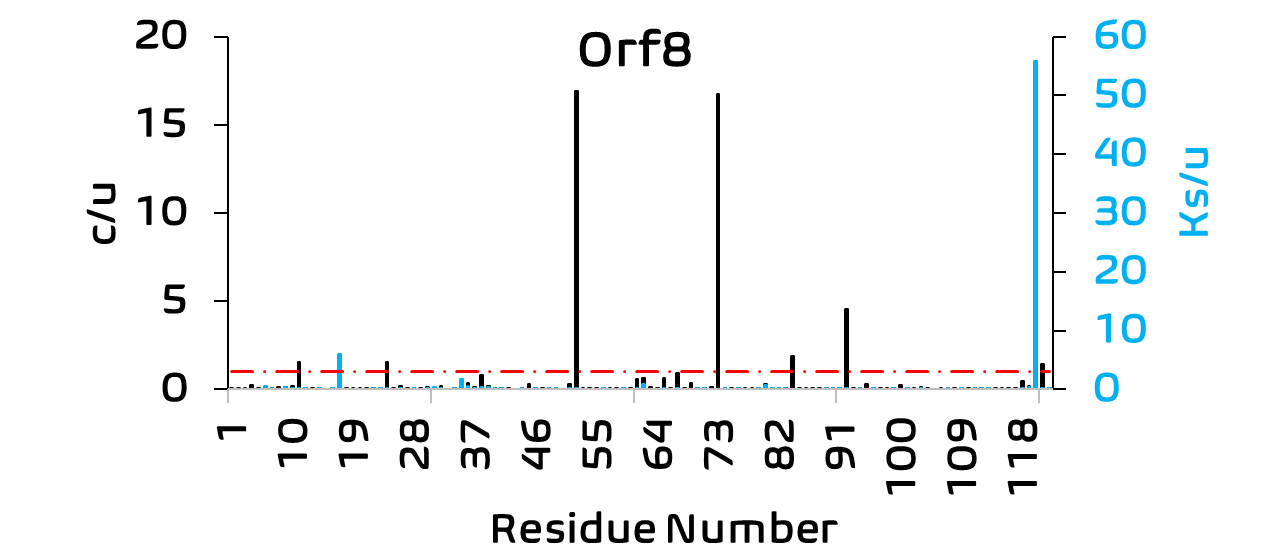 | 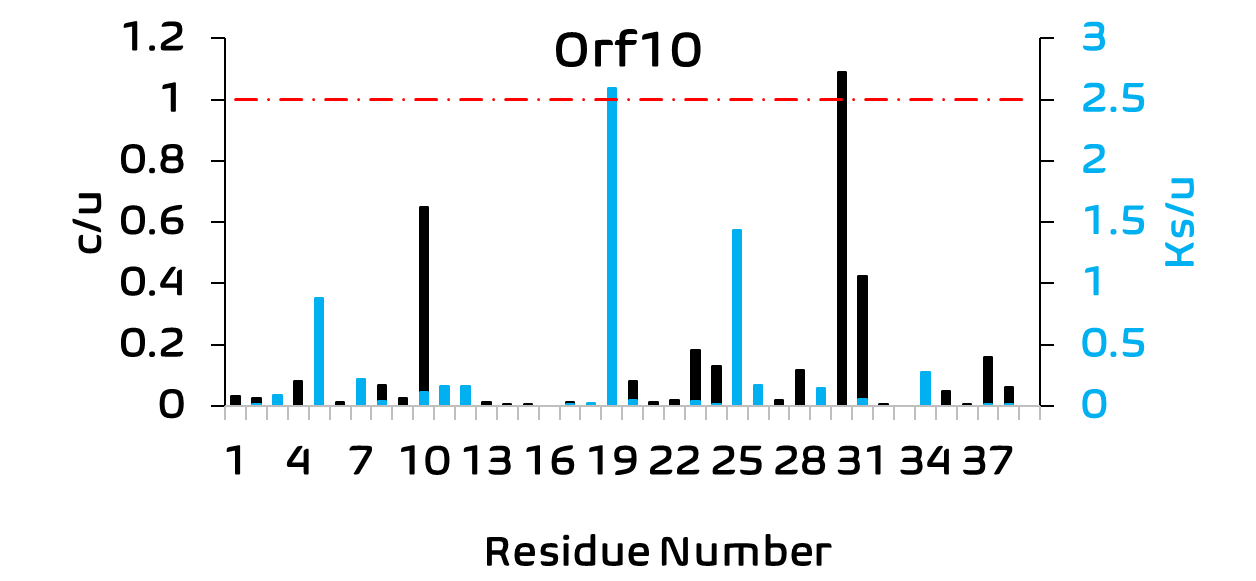 |

**Figure S4.** Position-based c/µ and Ks/µ values for each major and accessory coding gene. Red line denotes c/μ >1 and Ks/μ >1, indicating positive selection.

| 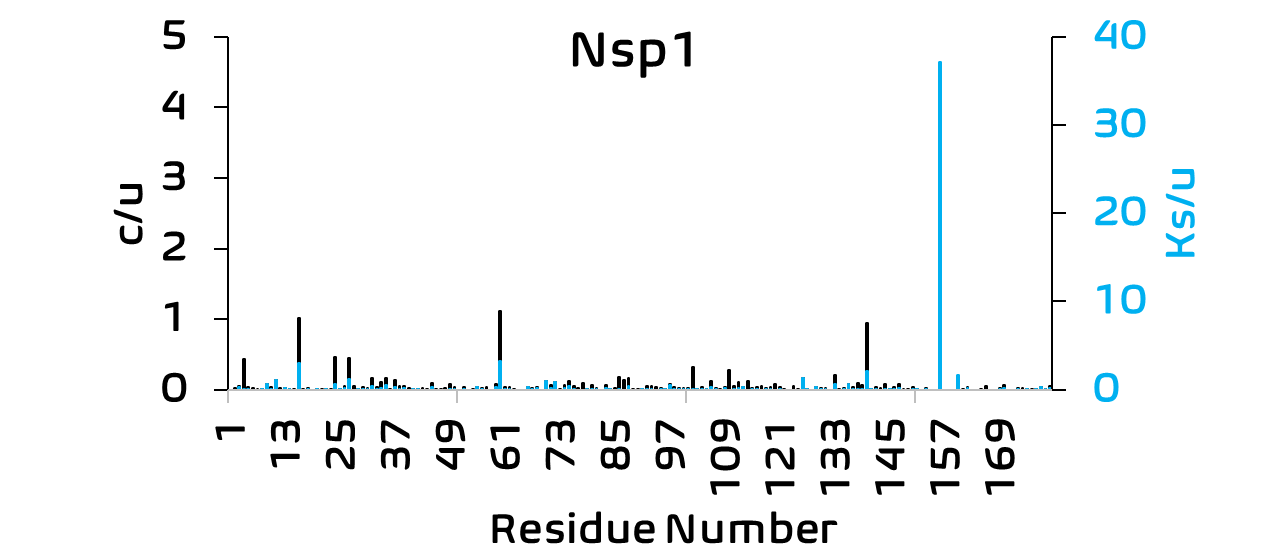 | 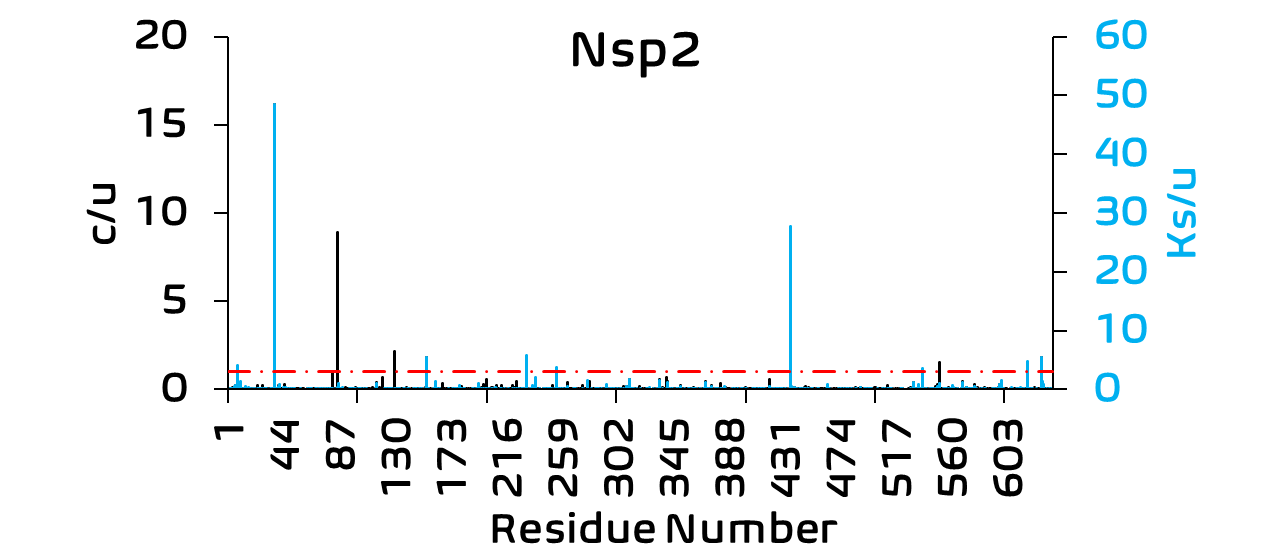 | 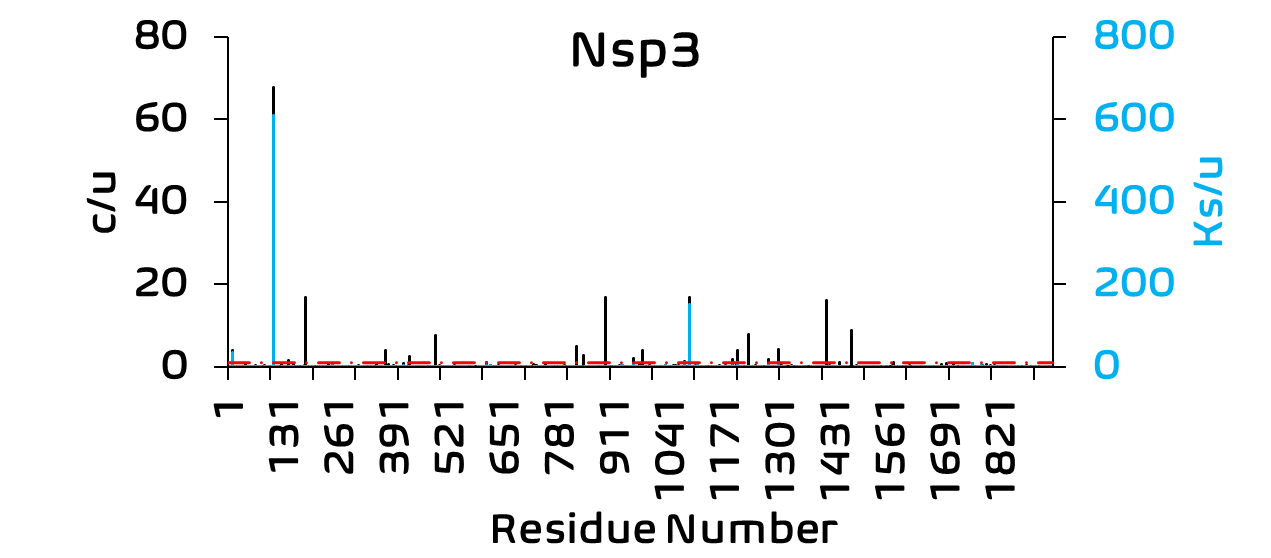 |
| --- | --- | --- |
| 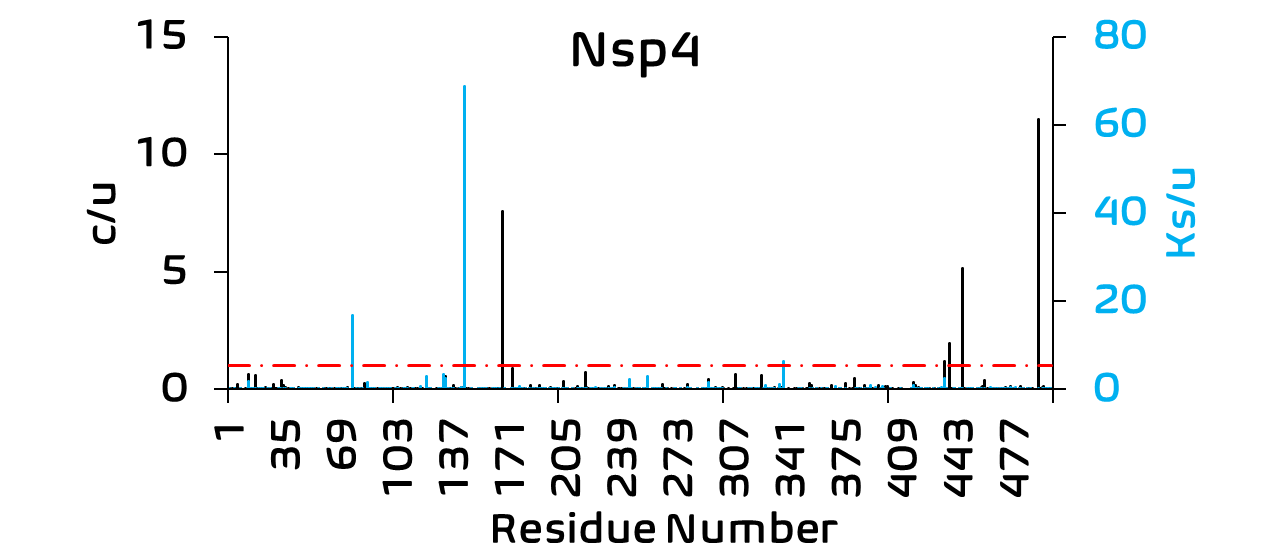 | 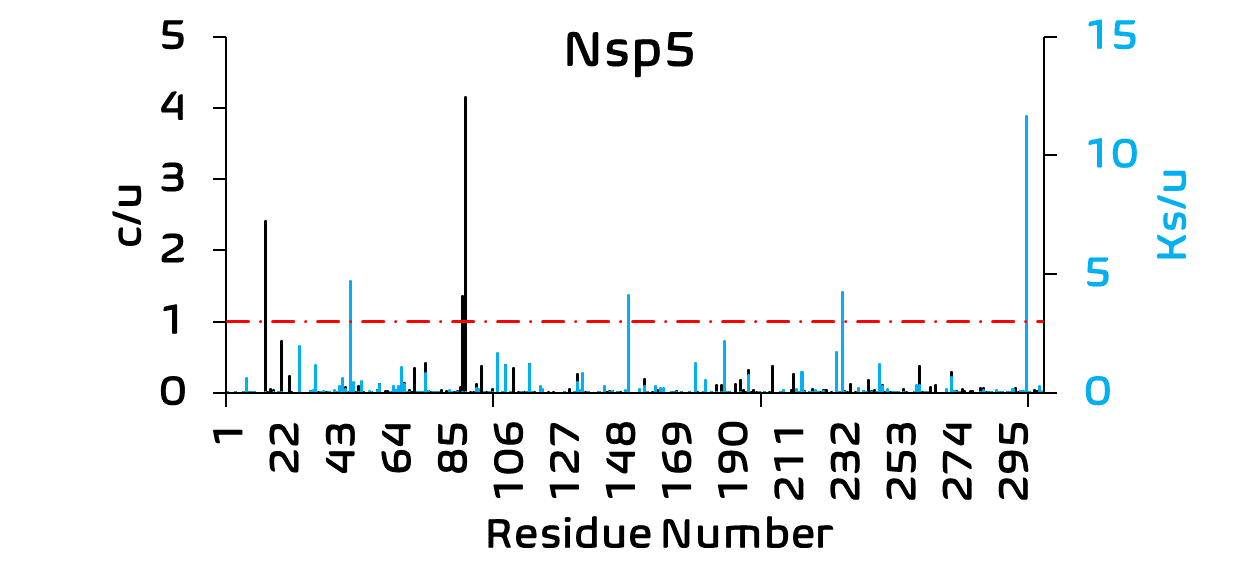 | 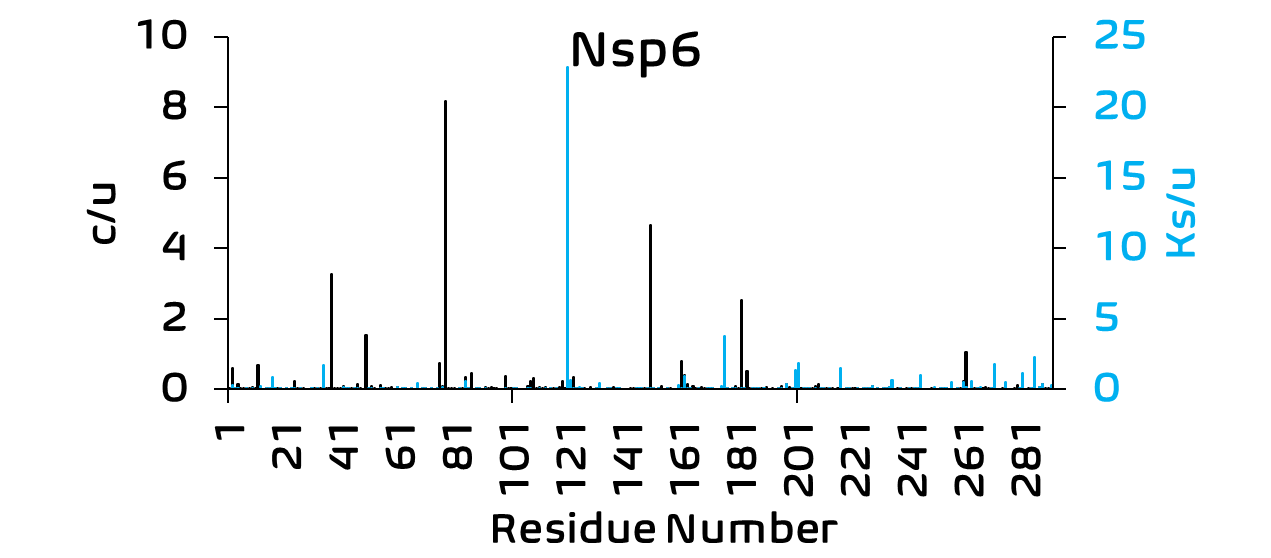 |
| 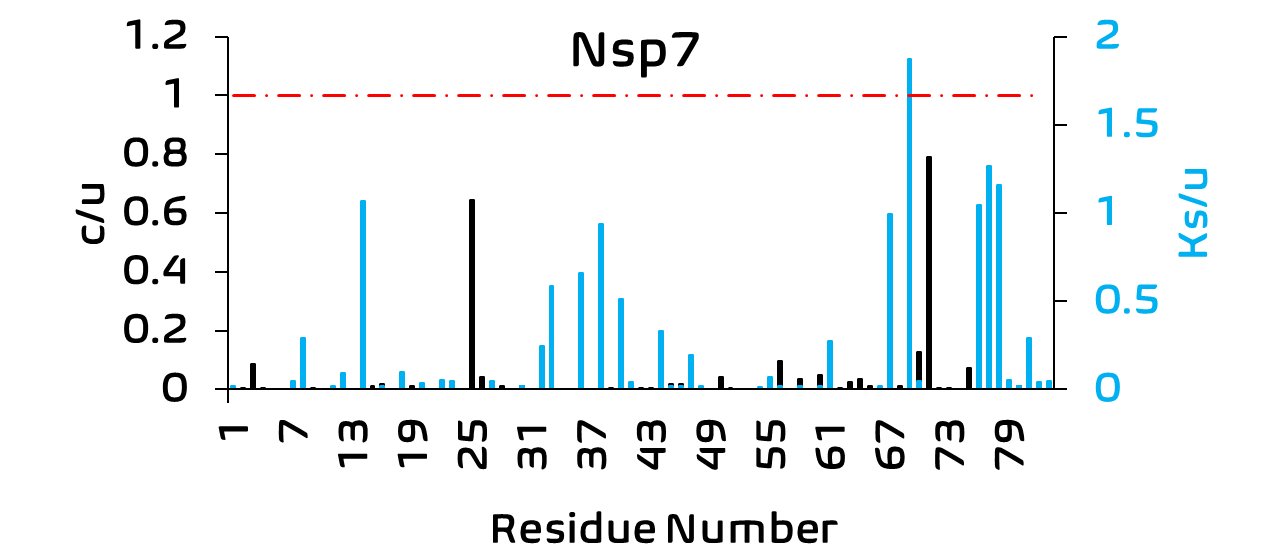 | 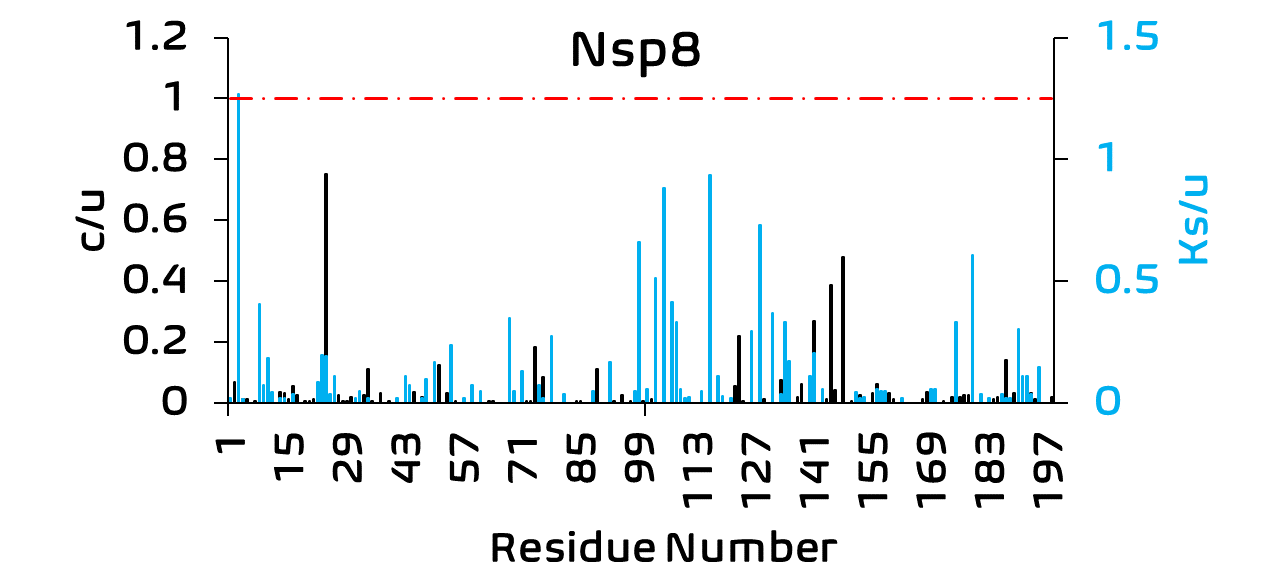 | 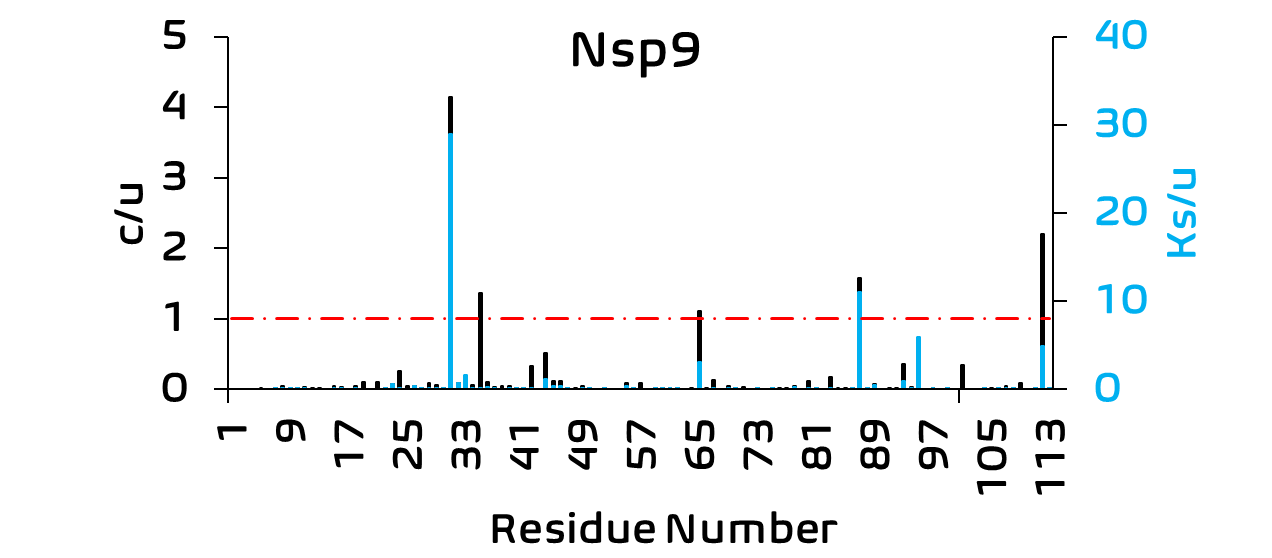 |
| 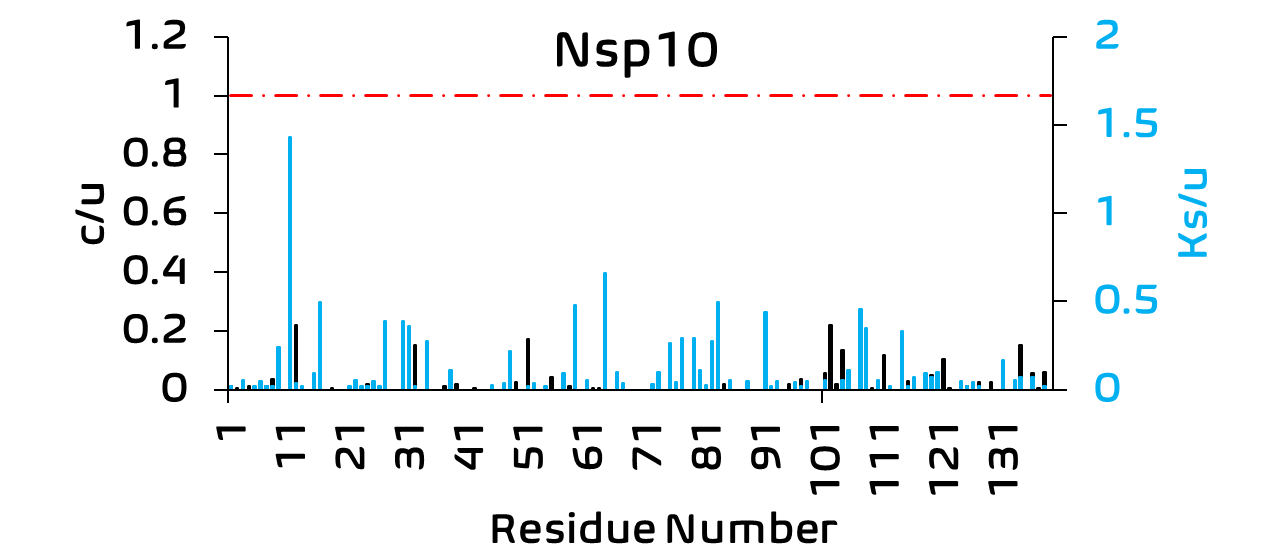 | 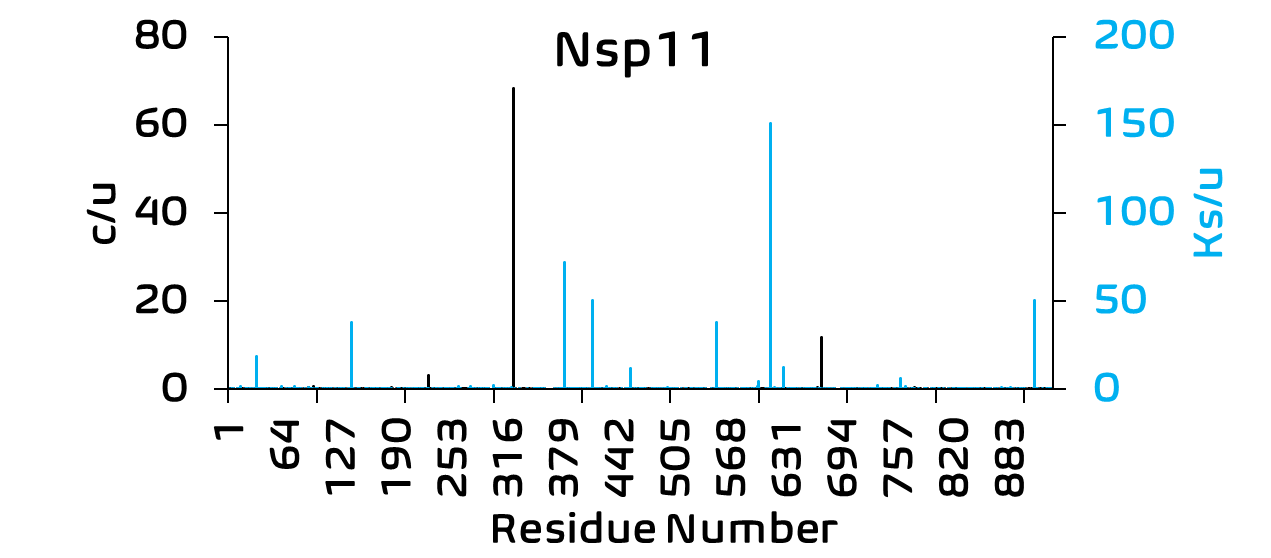 | 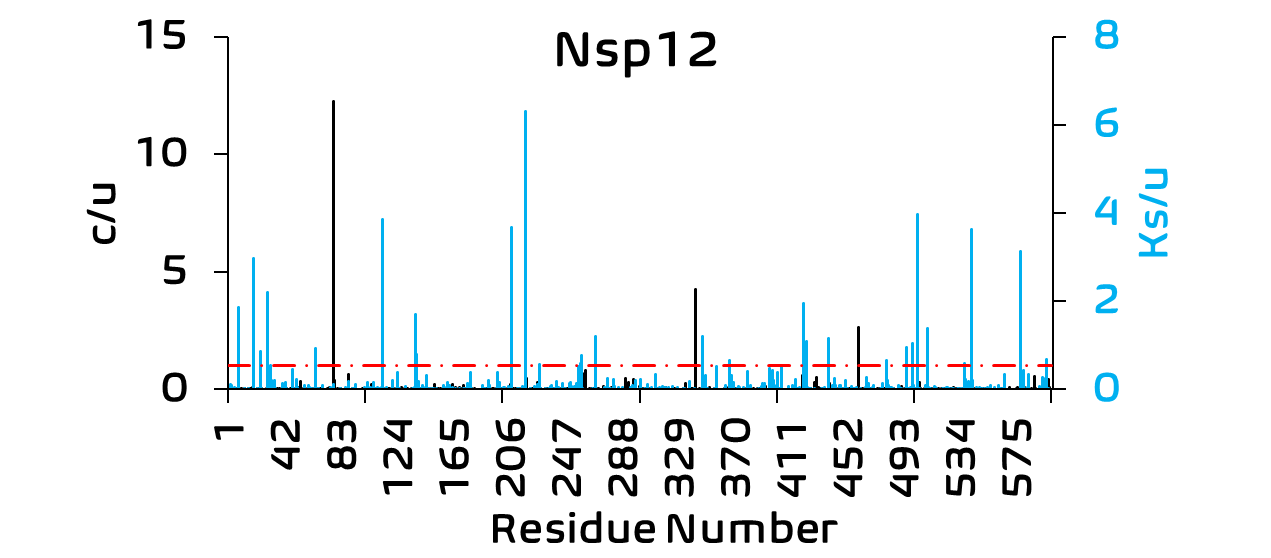 |
| 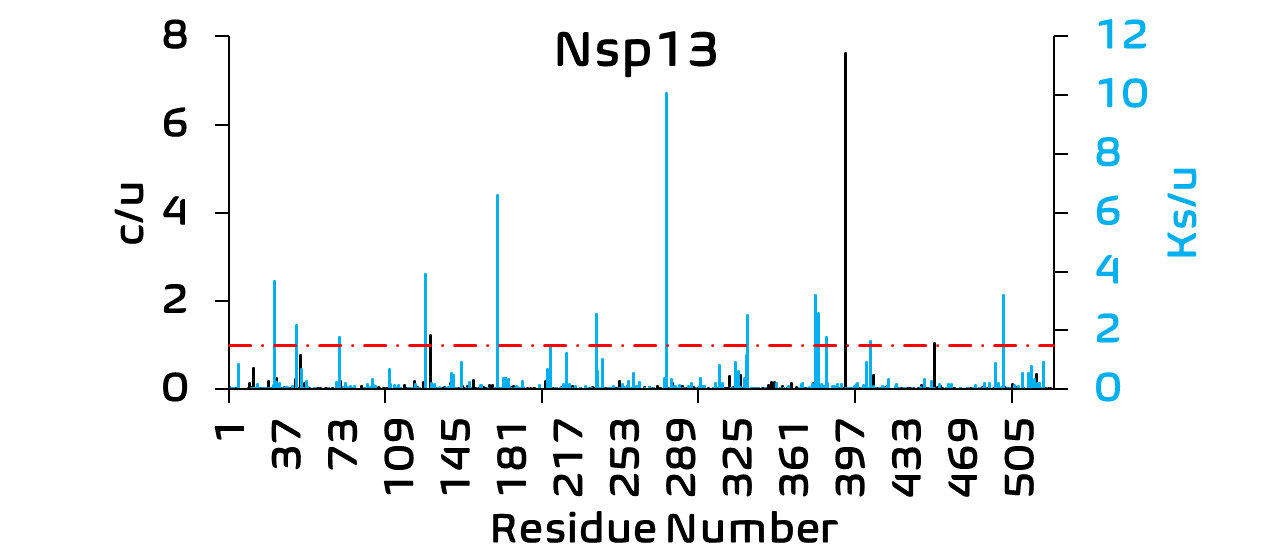 | 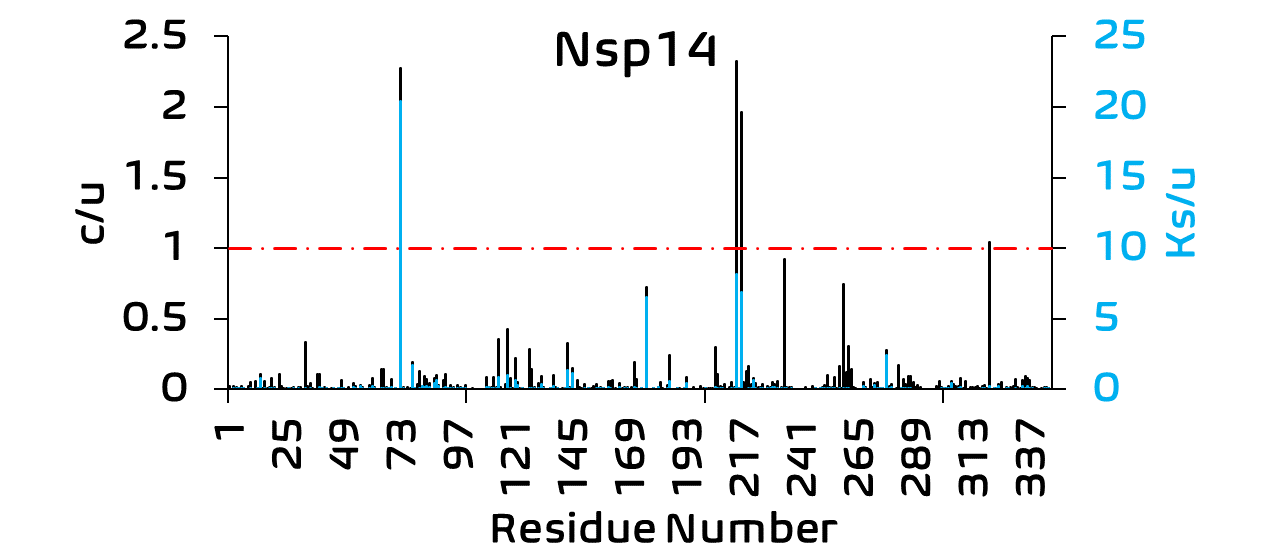 | 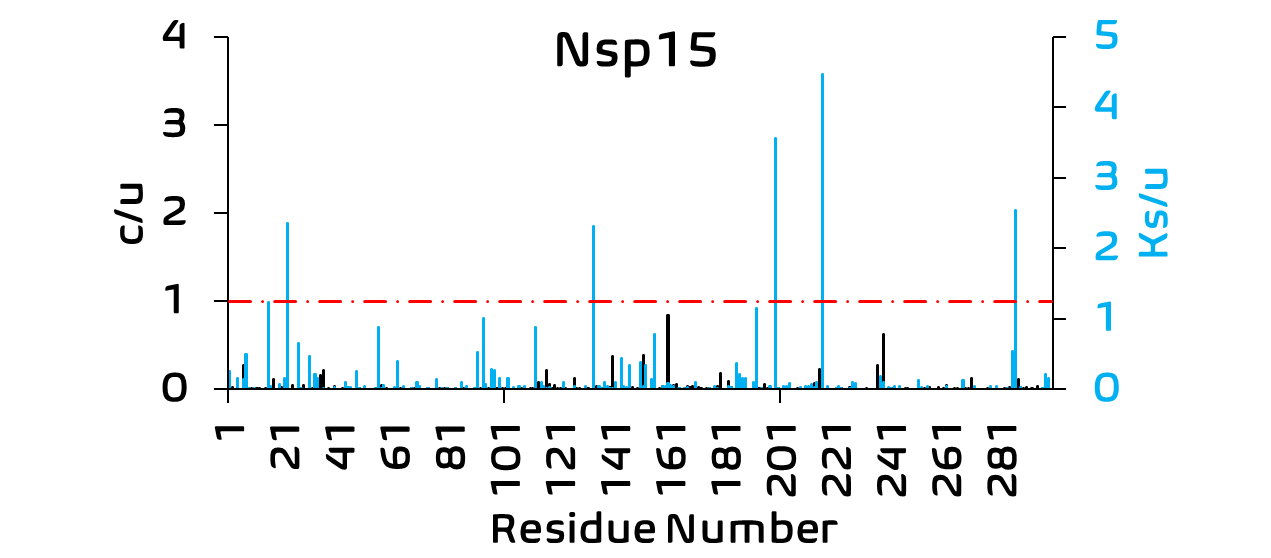 |

**Figure S5.** Position-based c/µ and Ks/µ values for NSP1-15. Red line denotes c/μ >1 and Ks/μ >1, indicating positive selection.

| 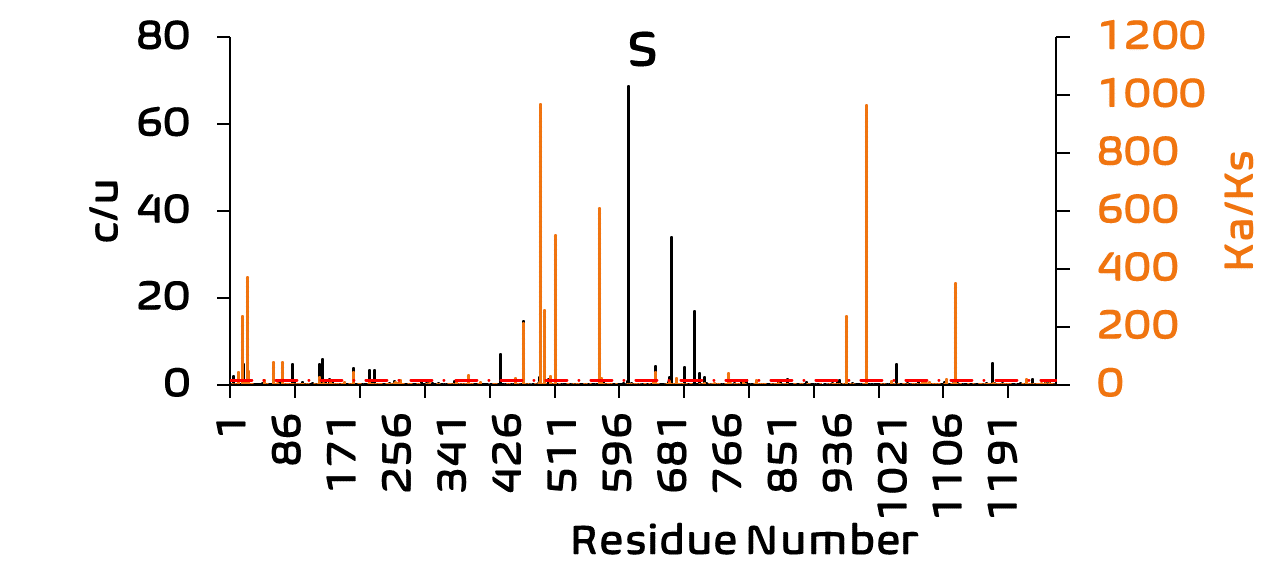 | 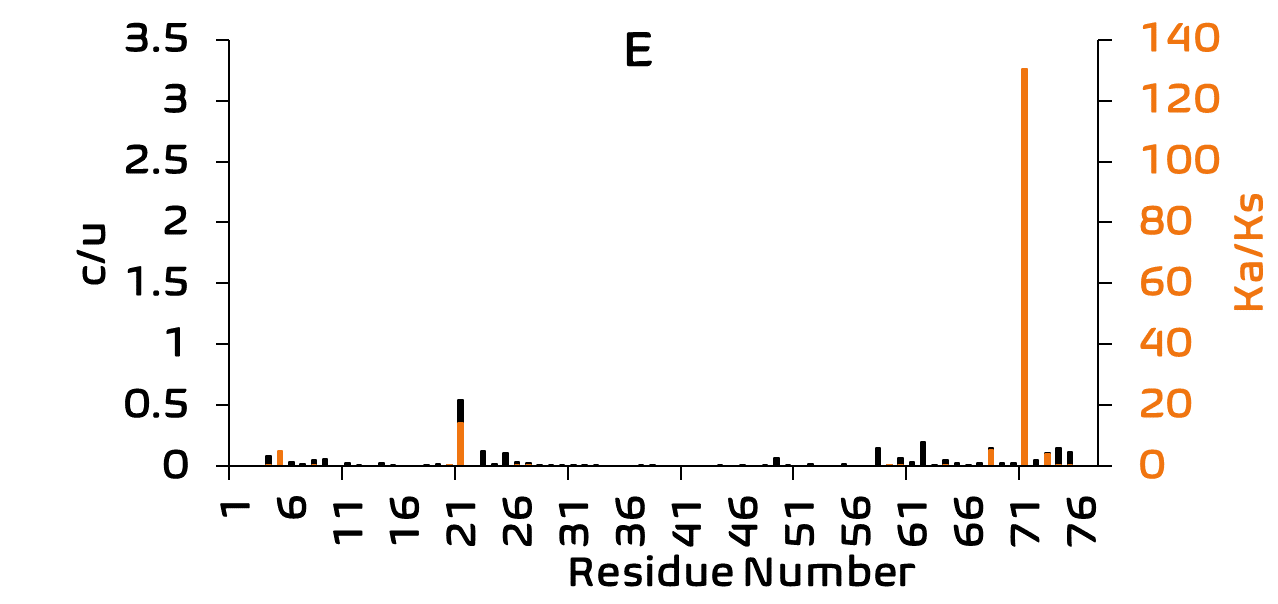 | 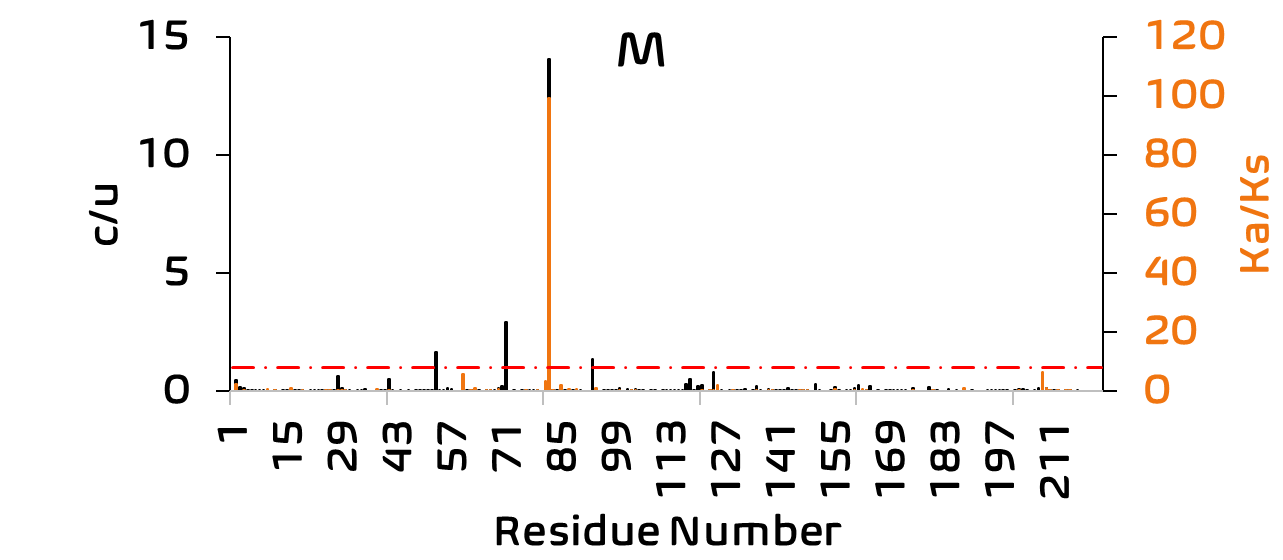 |
| --- | --- | --- |
| 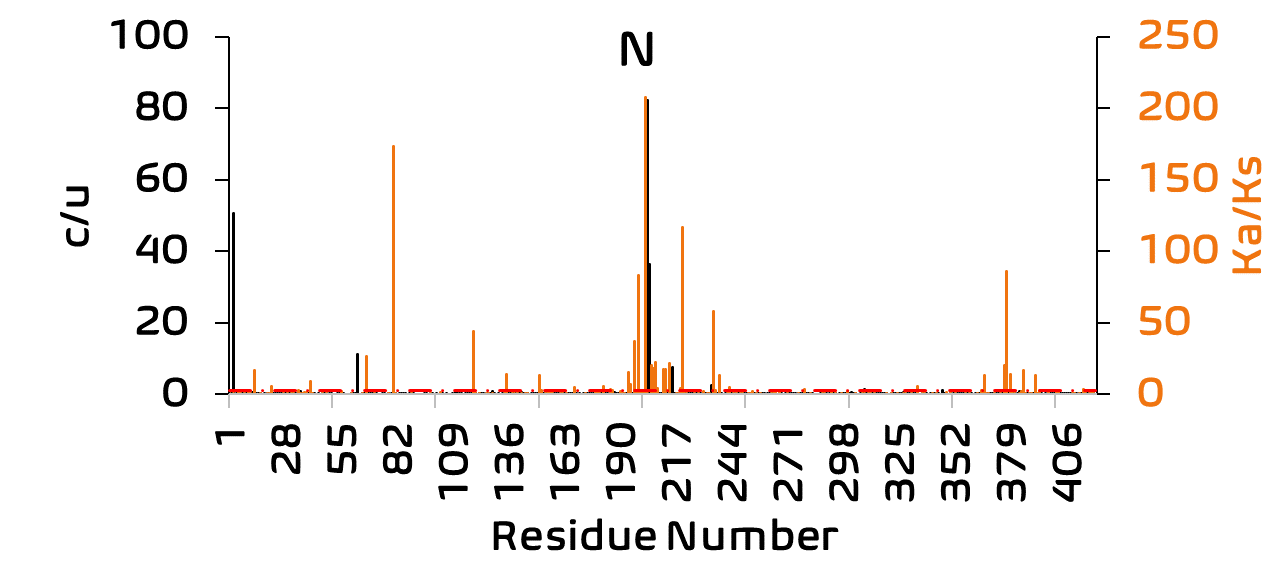 | 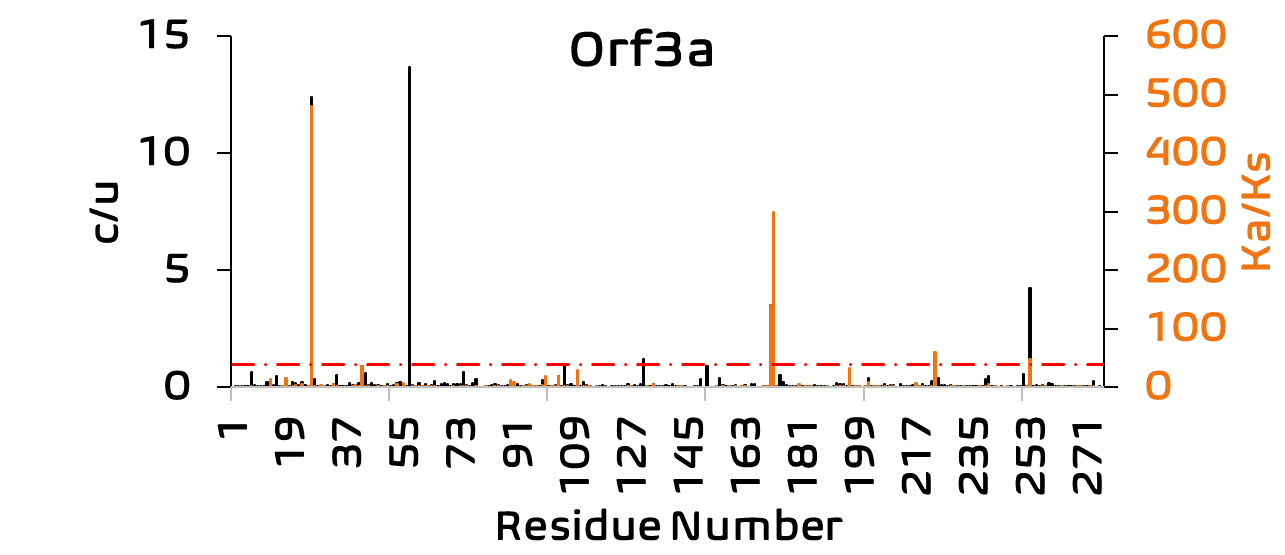 | 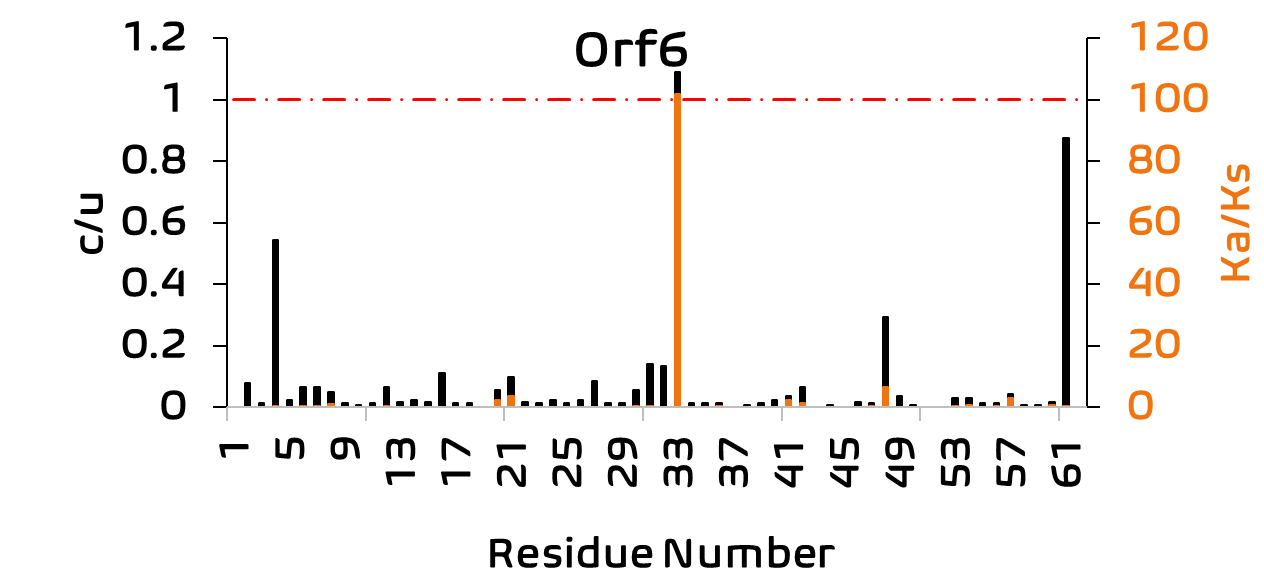 |
| 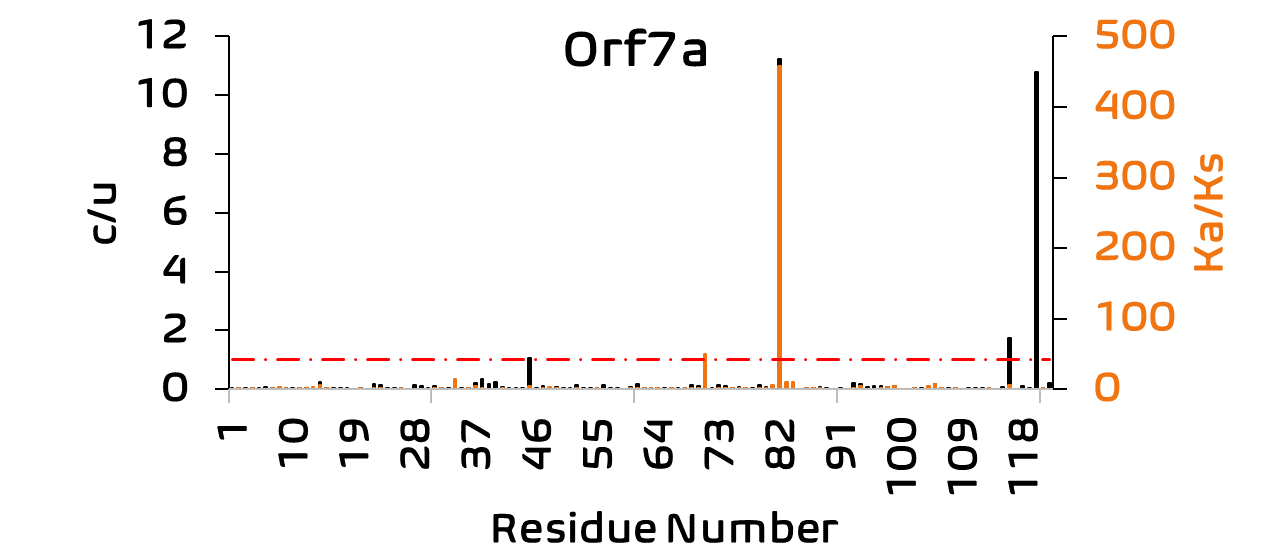 | 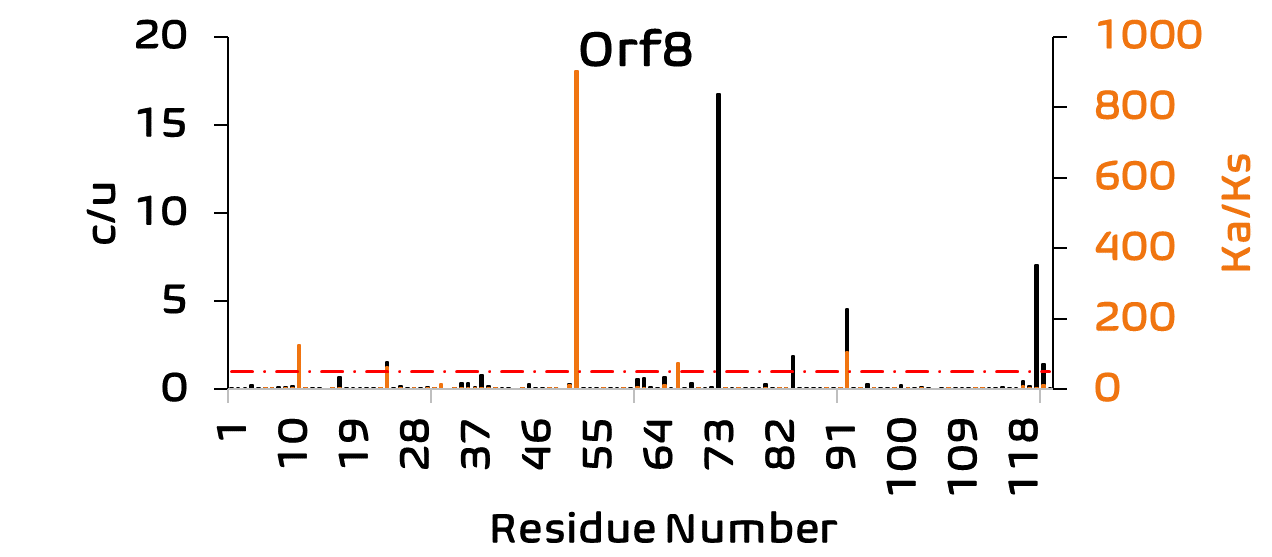 | 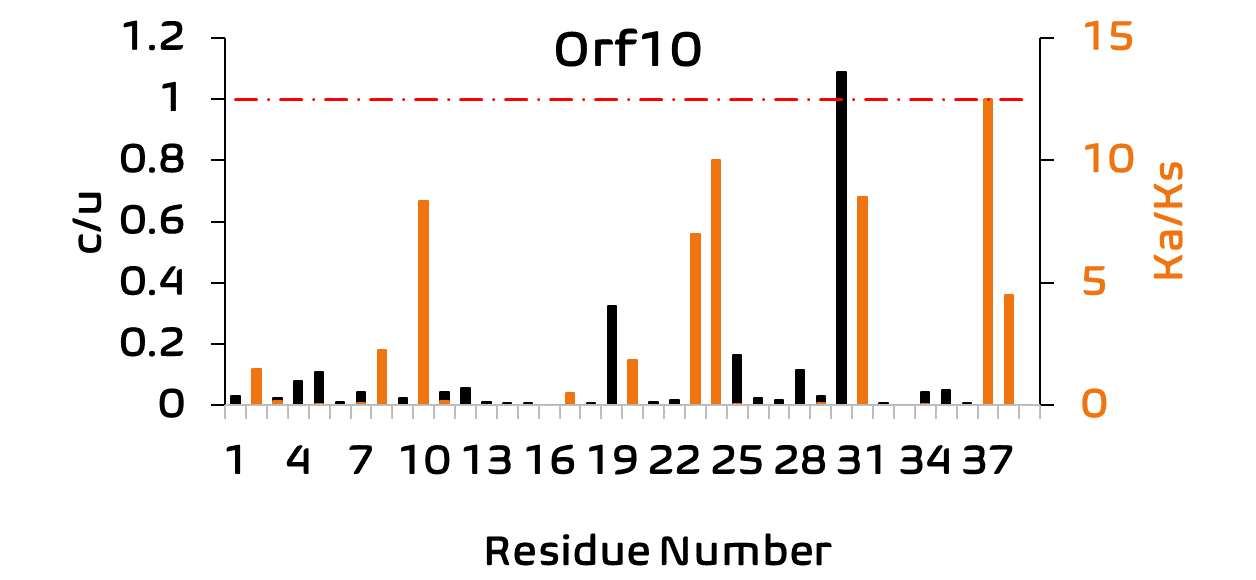 |

**Figure S6**. Position-based c/µ and Ka/Ks values for each major and accessory coding gene. Red line denotes c/μ >1 and Ka/Ks >1, indicating positive selection.

| 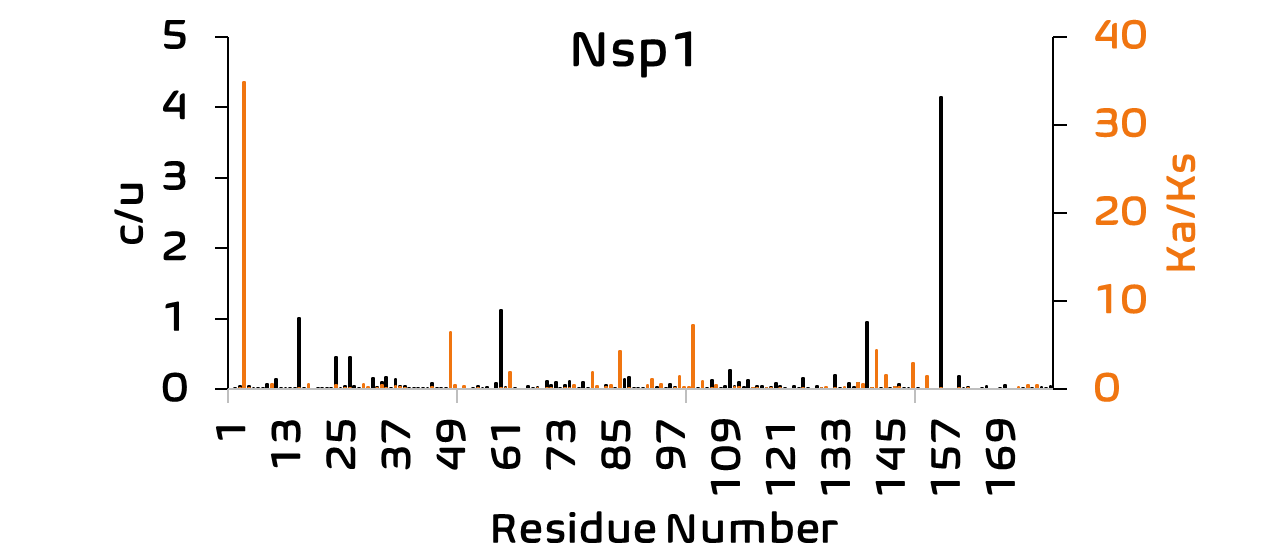 | 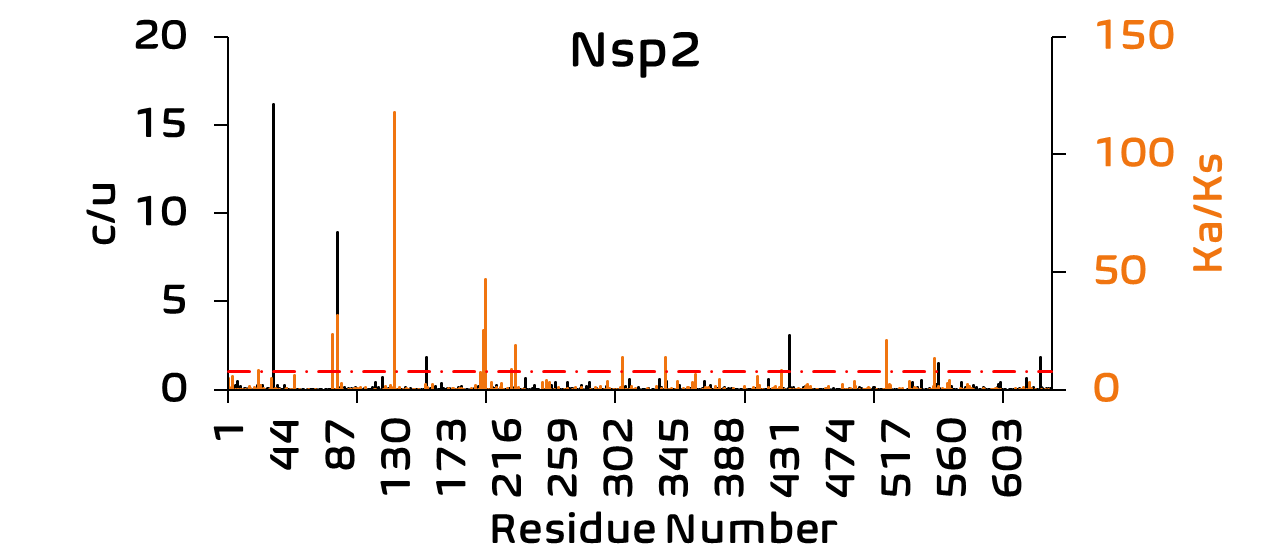 | 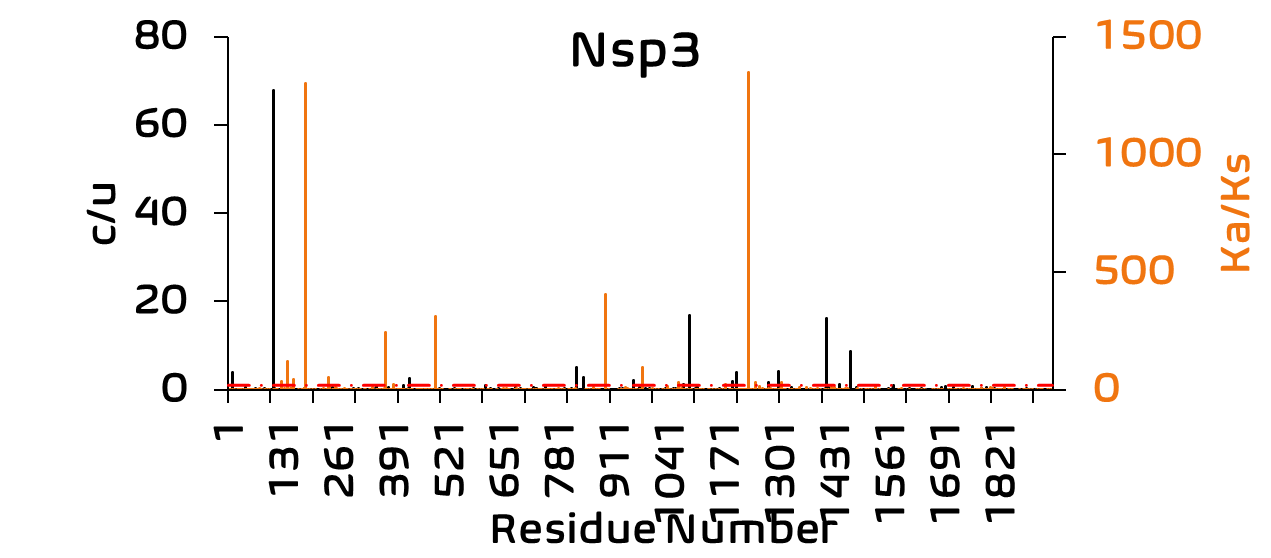 |
| --- | --- | --- |
| 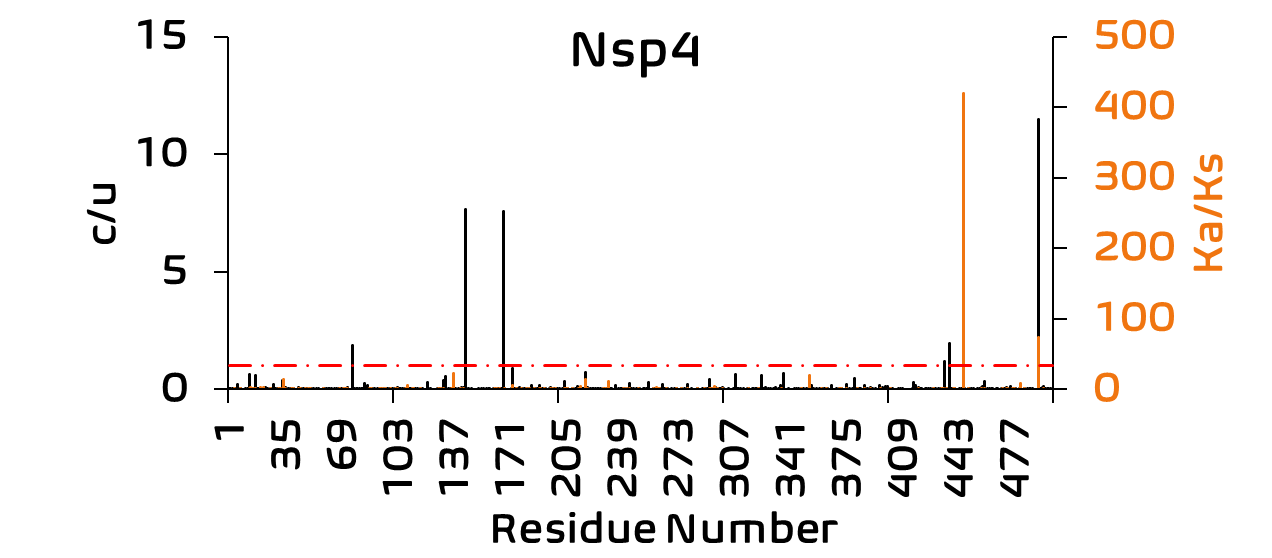 | 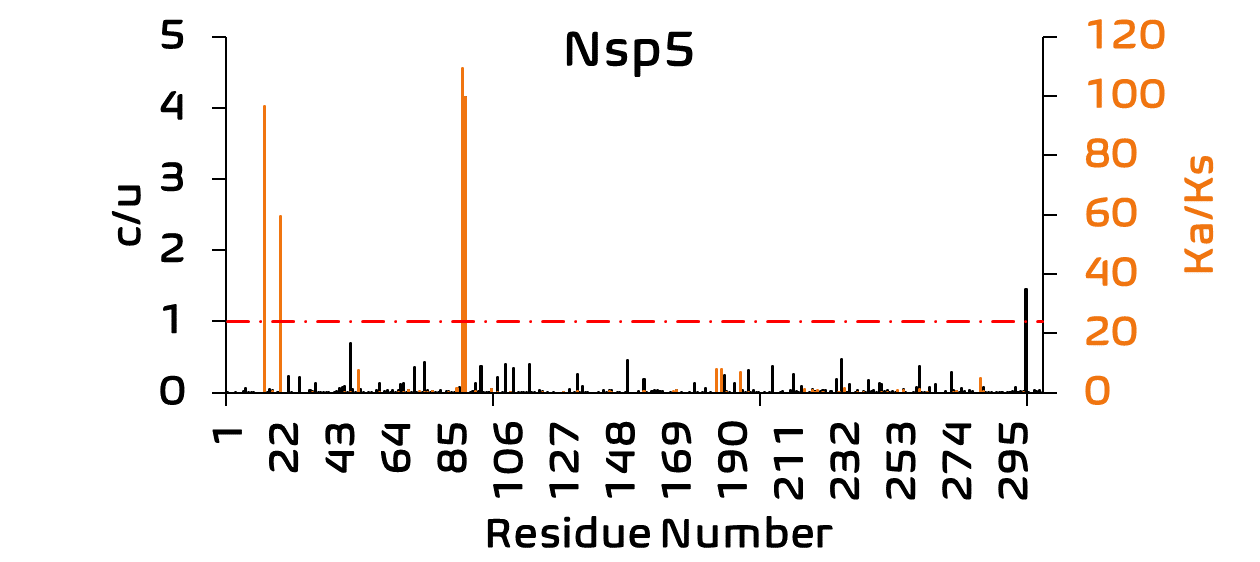 | 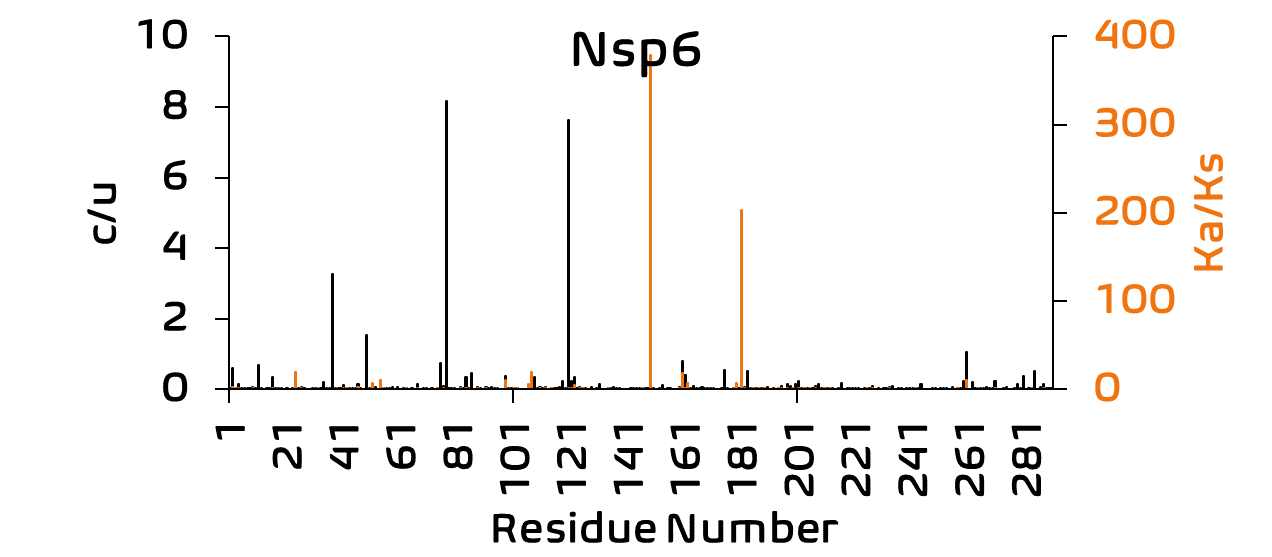 |
| 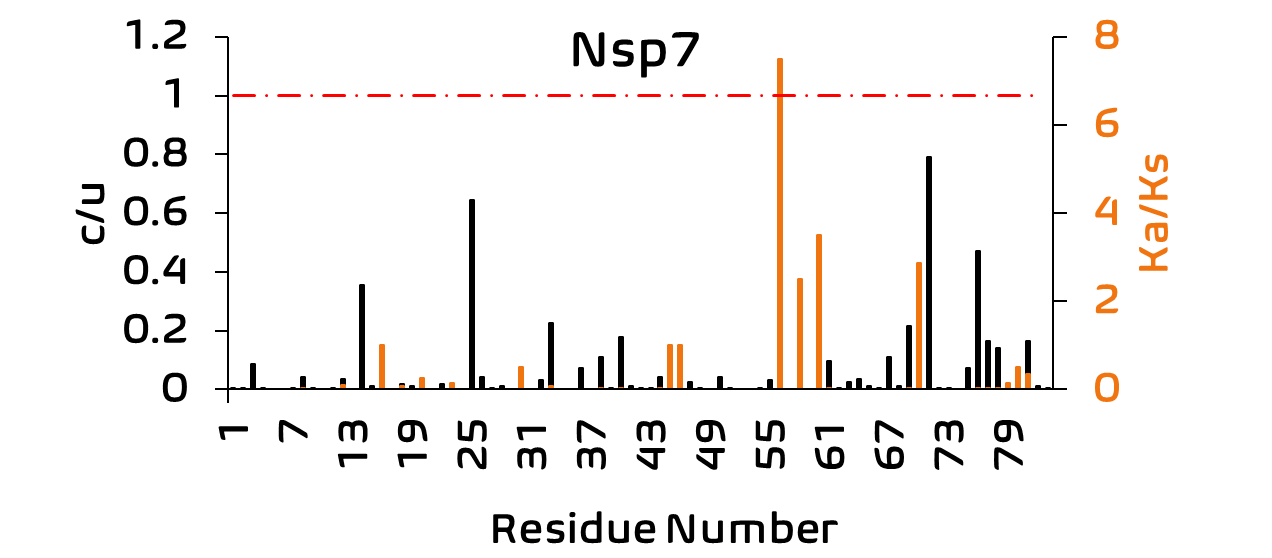 | 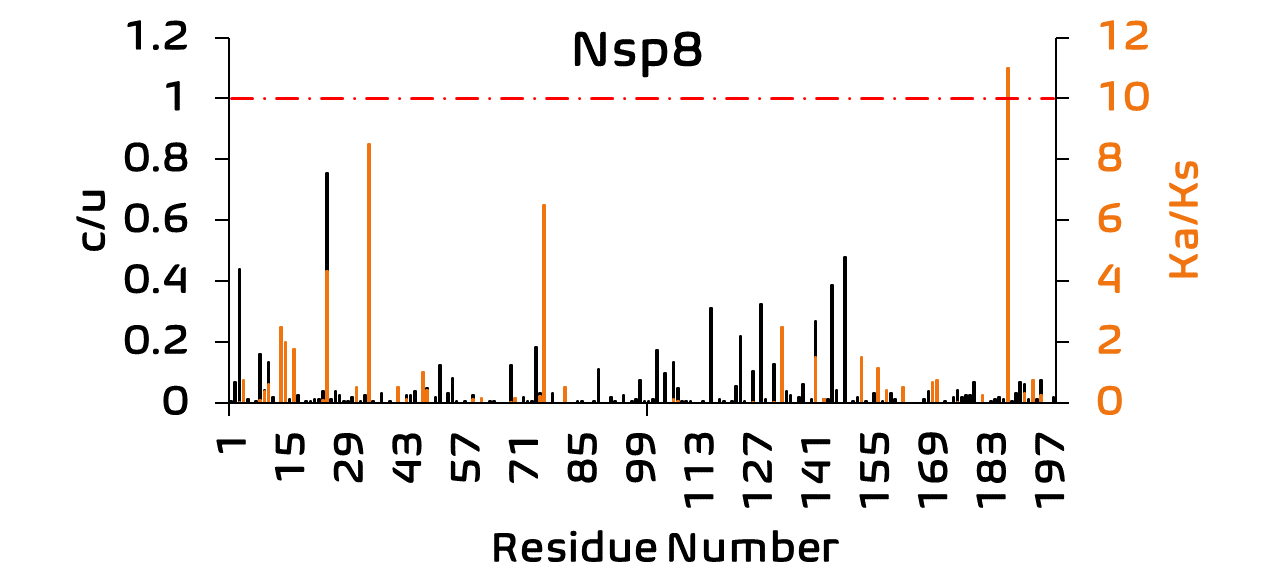 | 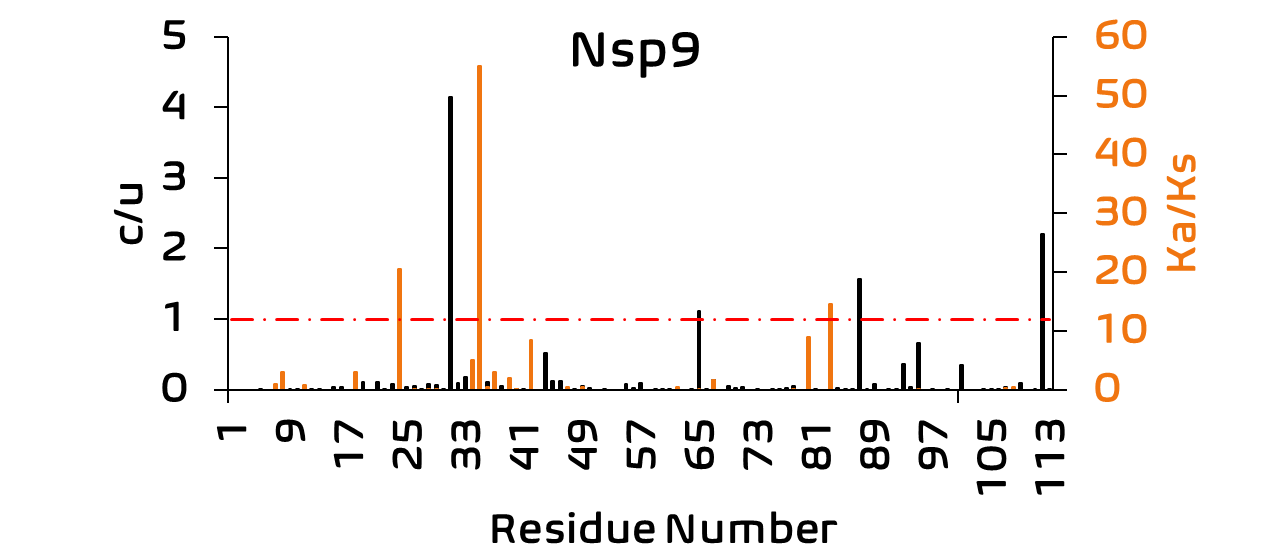 |
| 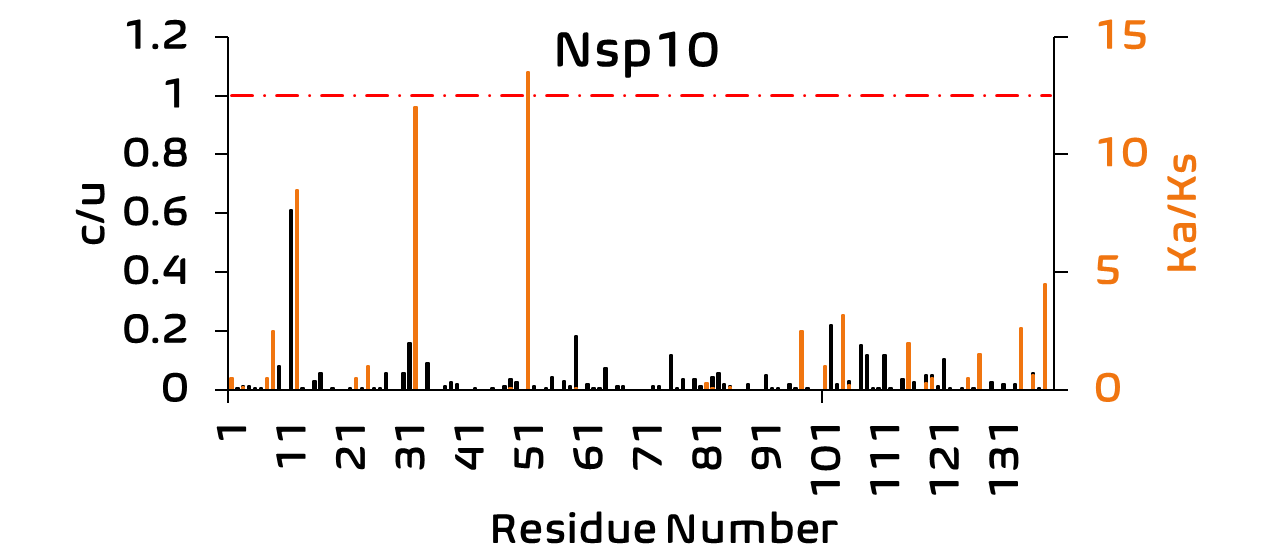 | 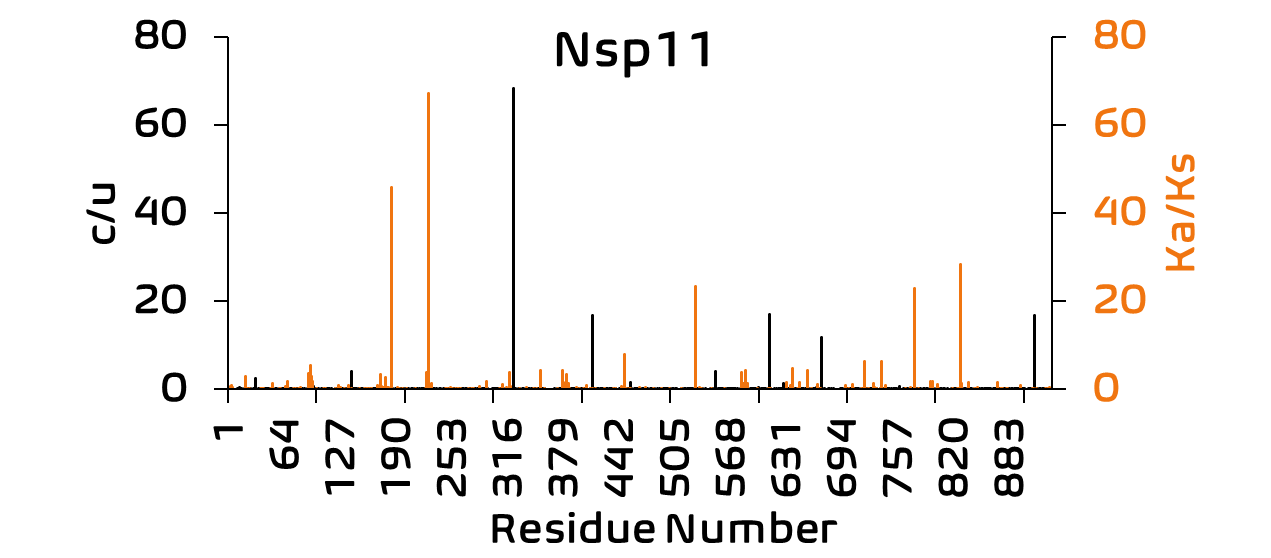 | 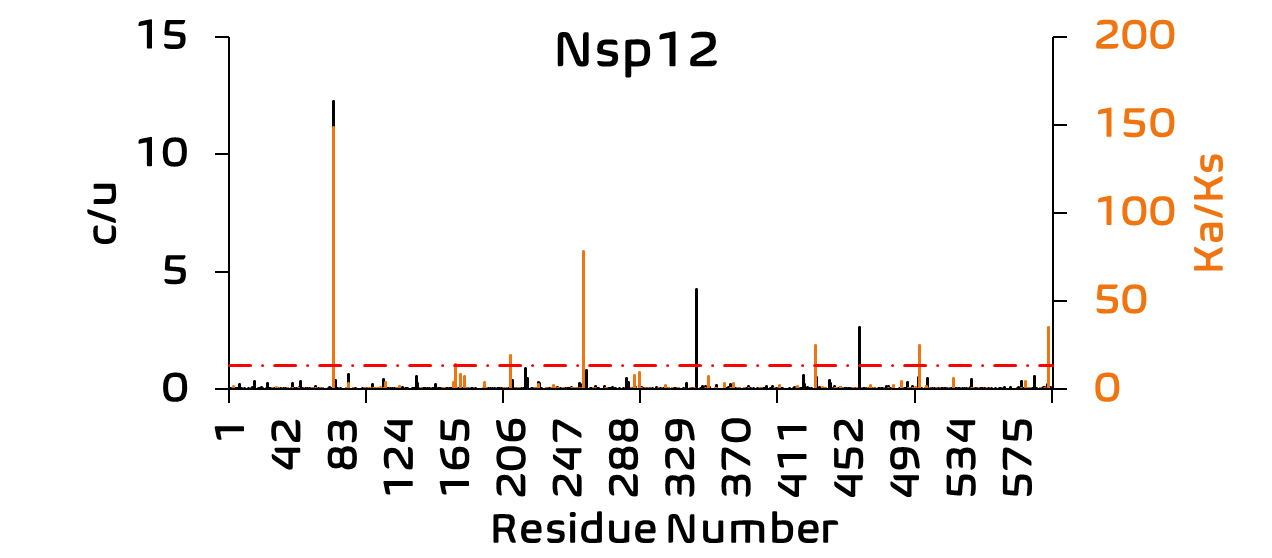 |
| 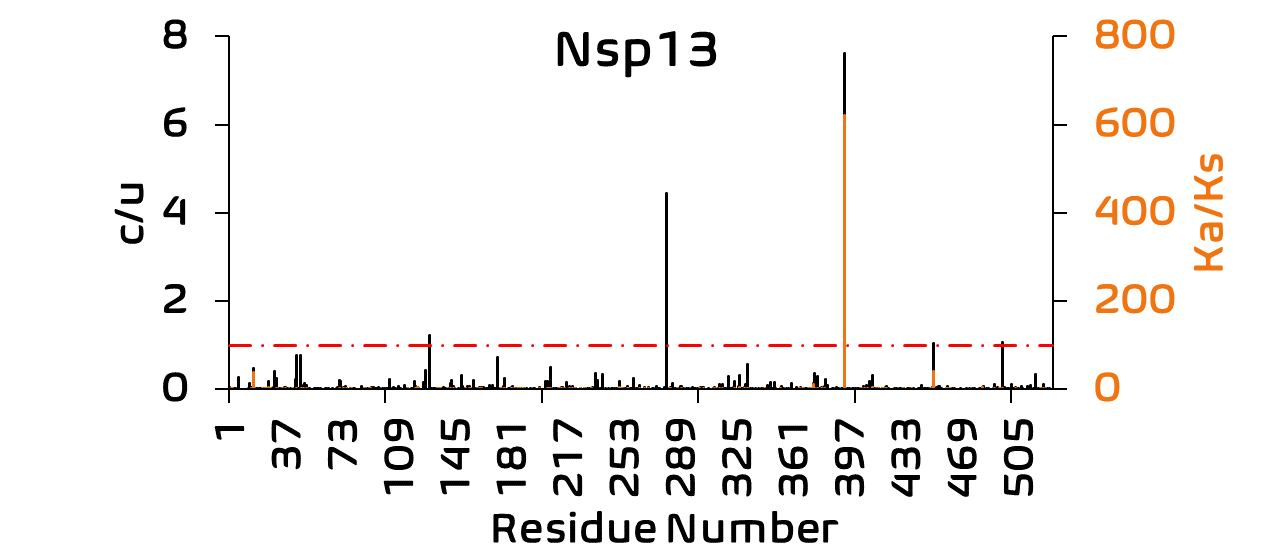 | 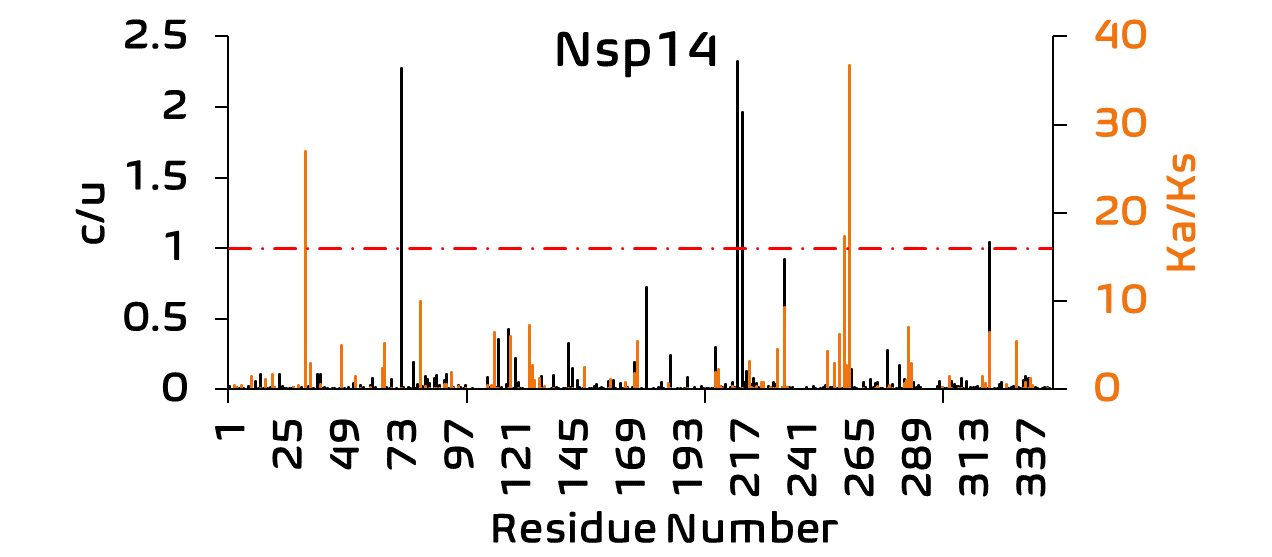 | 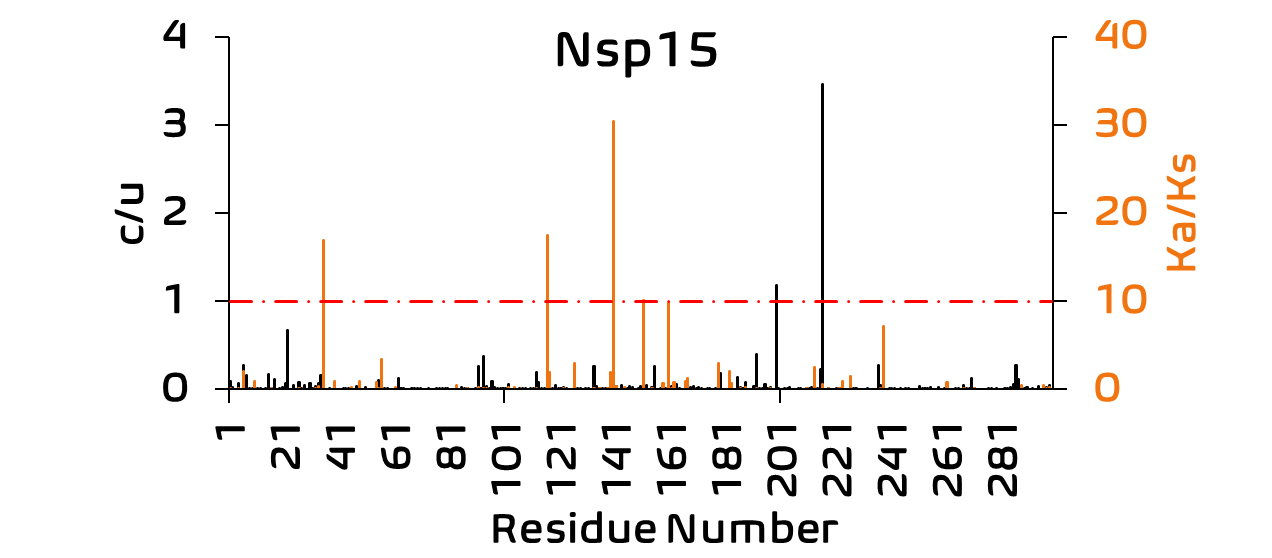 |

**Figure S7.** Position-based c/µ and Ka/Ks values for NSP1-15. Red line denotes c/μ >1 and Ka/Ks > 1, indicating positive selection.


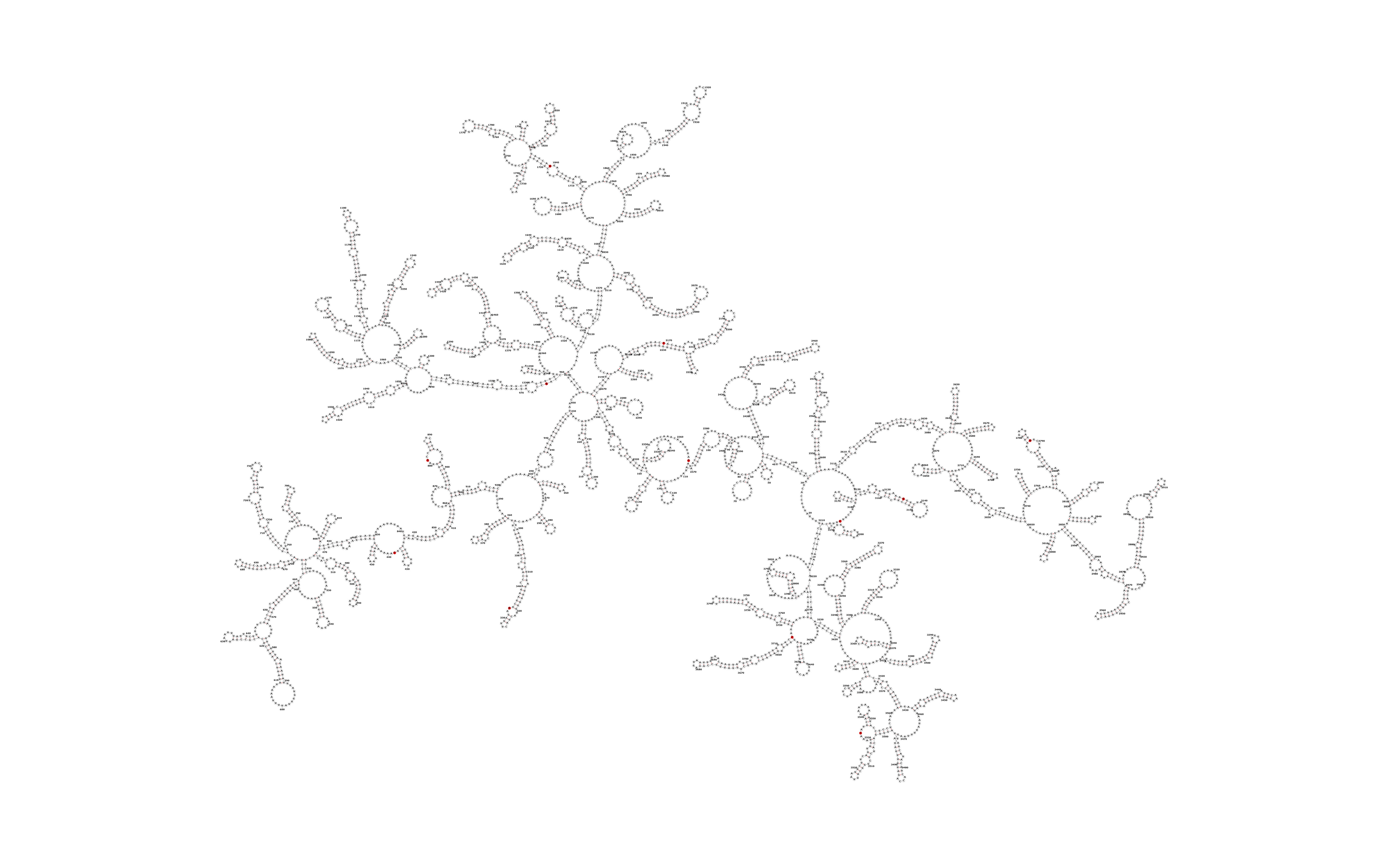


**Figure S8a.** S RNA secondary structure. Red circles represent top synonymous NT mutations.


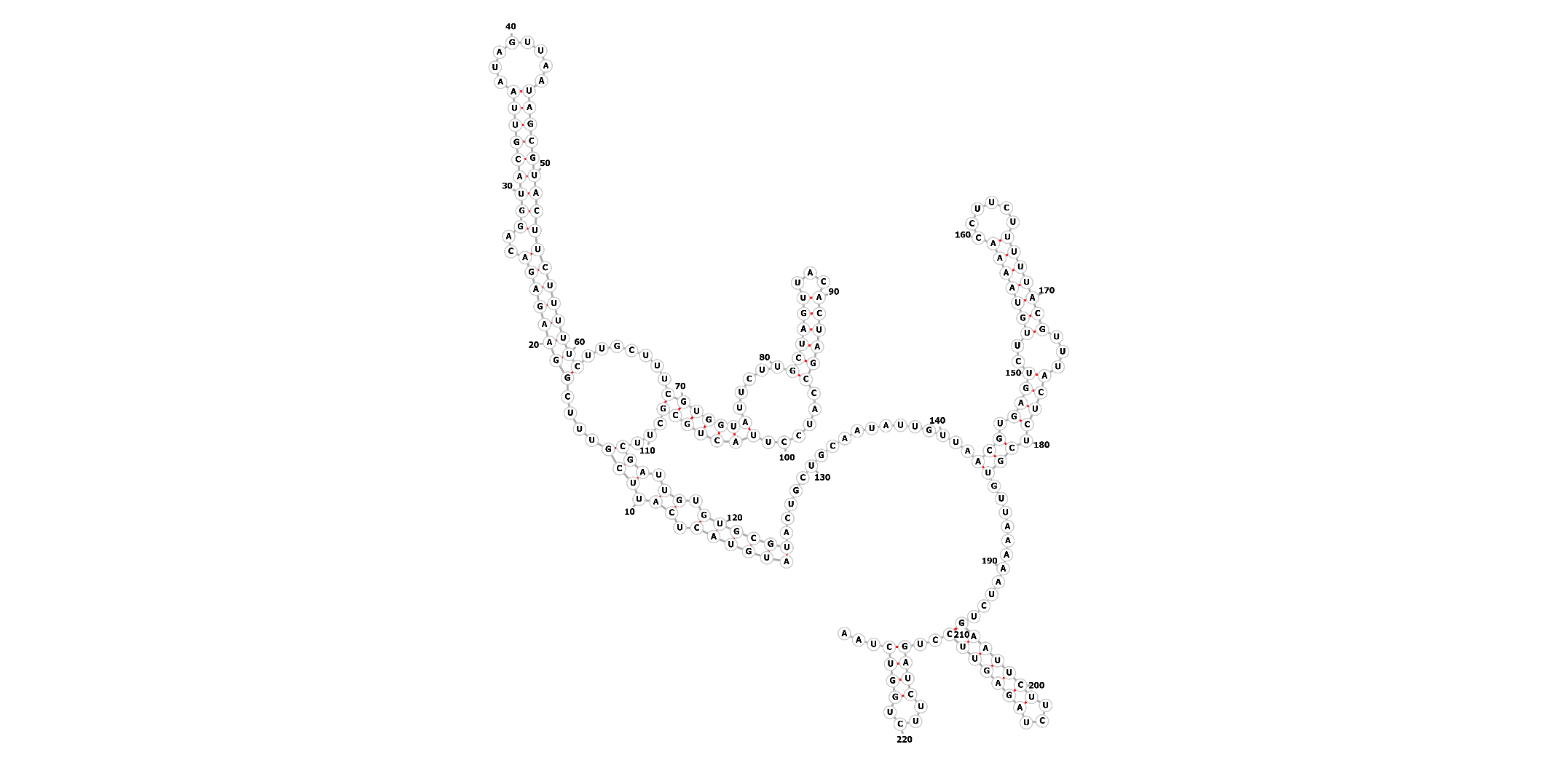


**Figure S8b.** E RNA secondary structure.


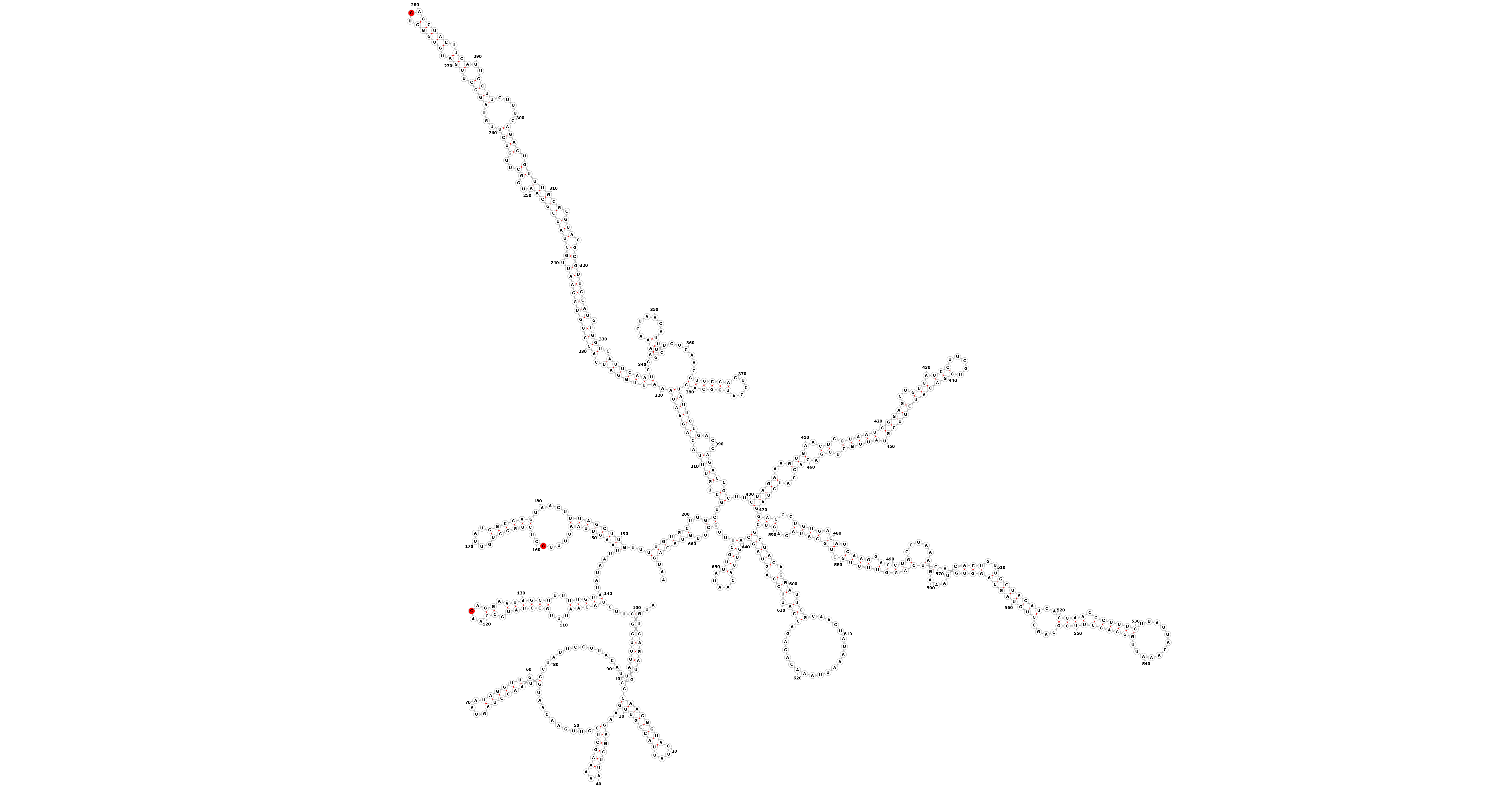


**Figure S8c.** M RNA secondary structure. Red circles represent top synonymous NT mutations.


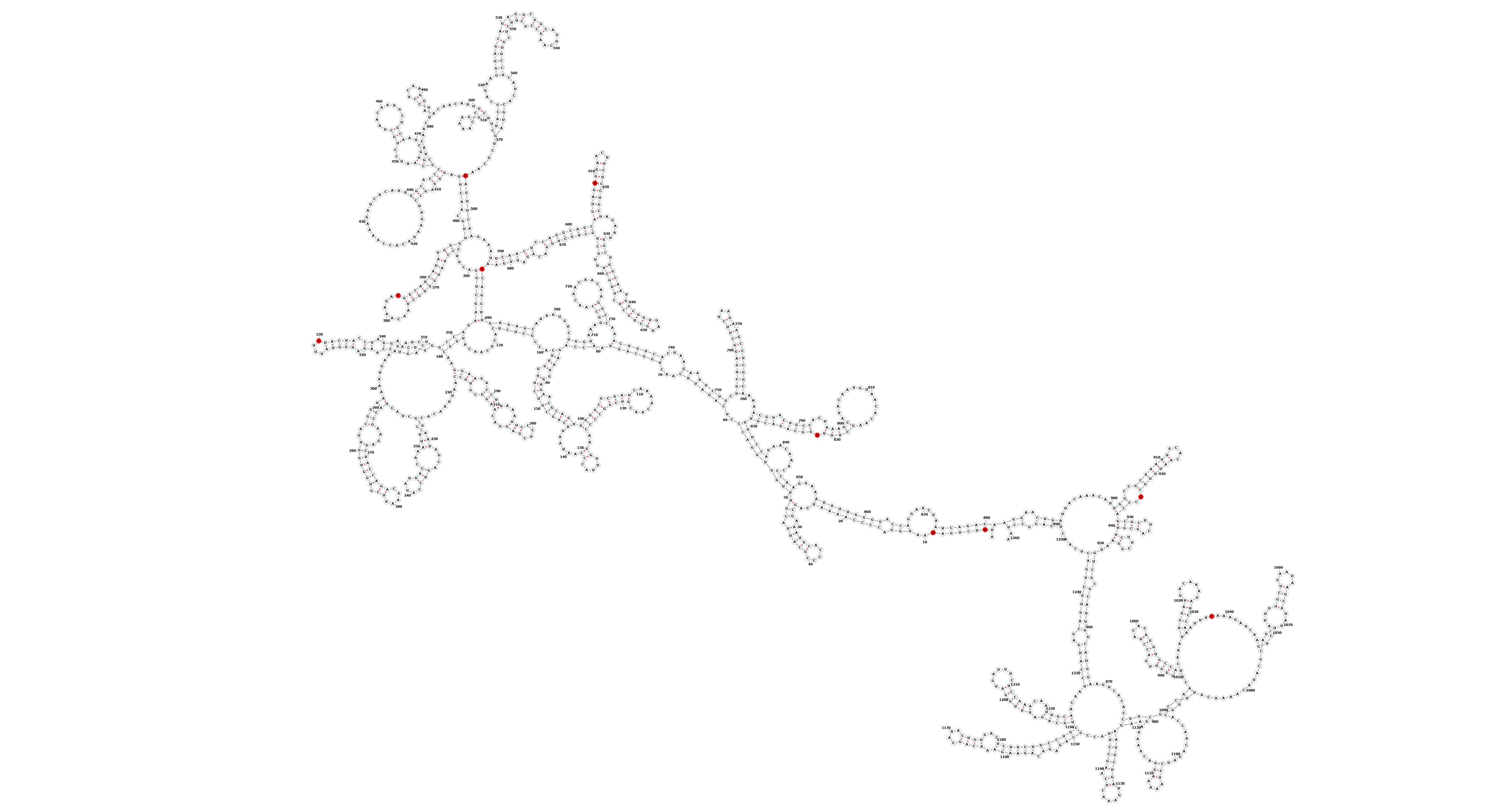


**Figure S8d.** N RNA secondary structure. Red circles represent top synonymous NT mutations.


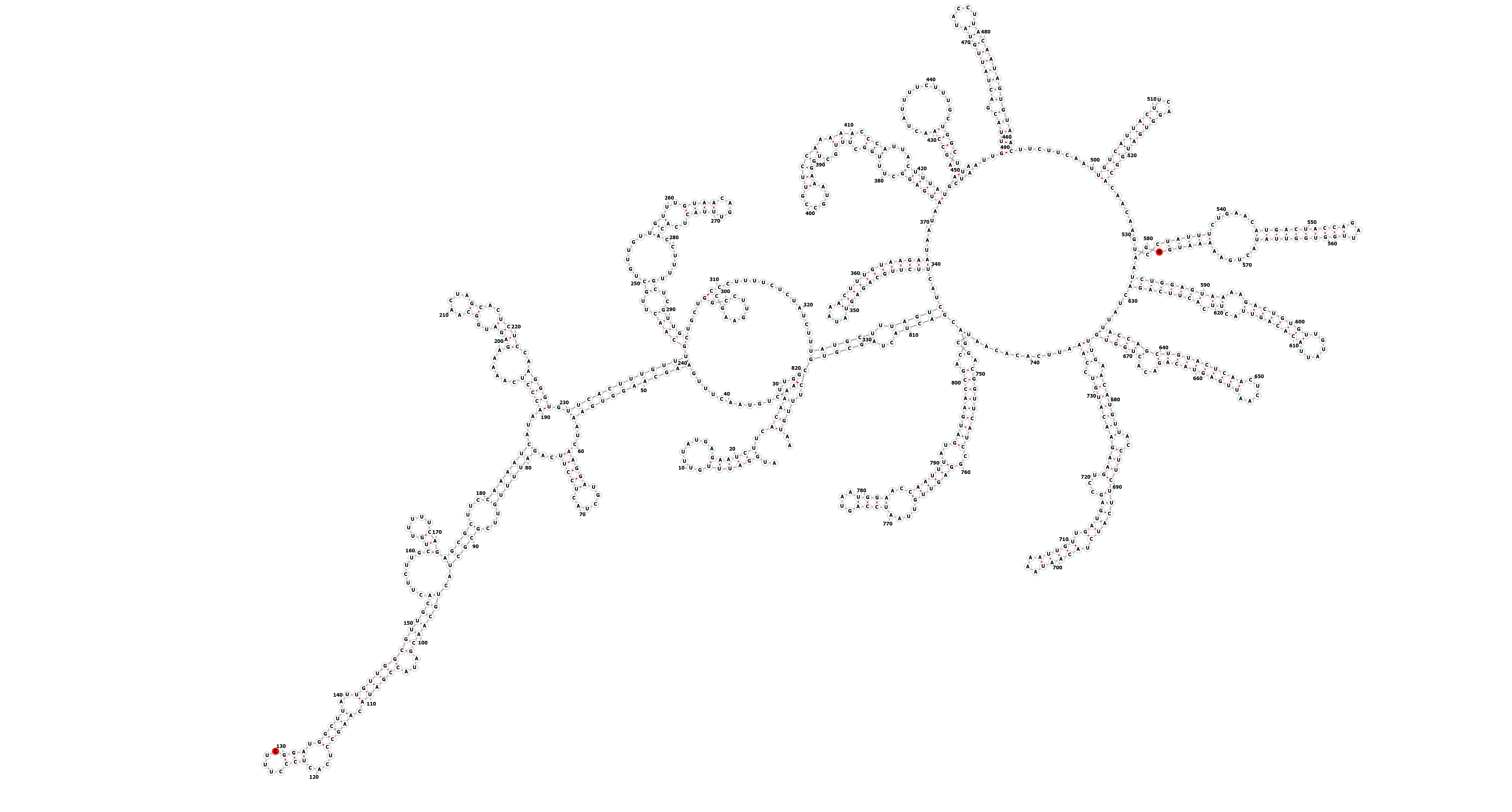


**Figure S8e.** Orf3a RNA secondary structure. Red circles represent top synonymous NT mutations.


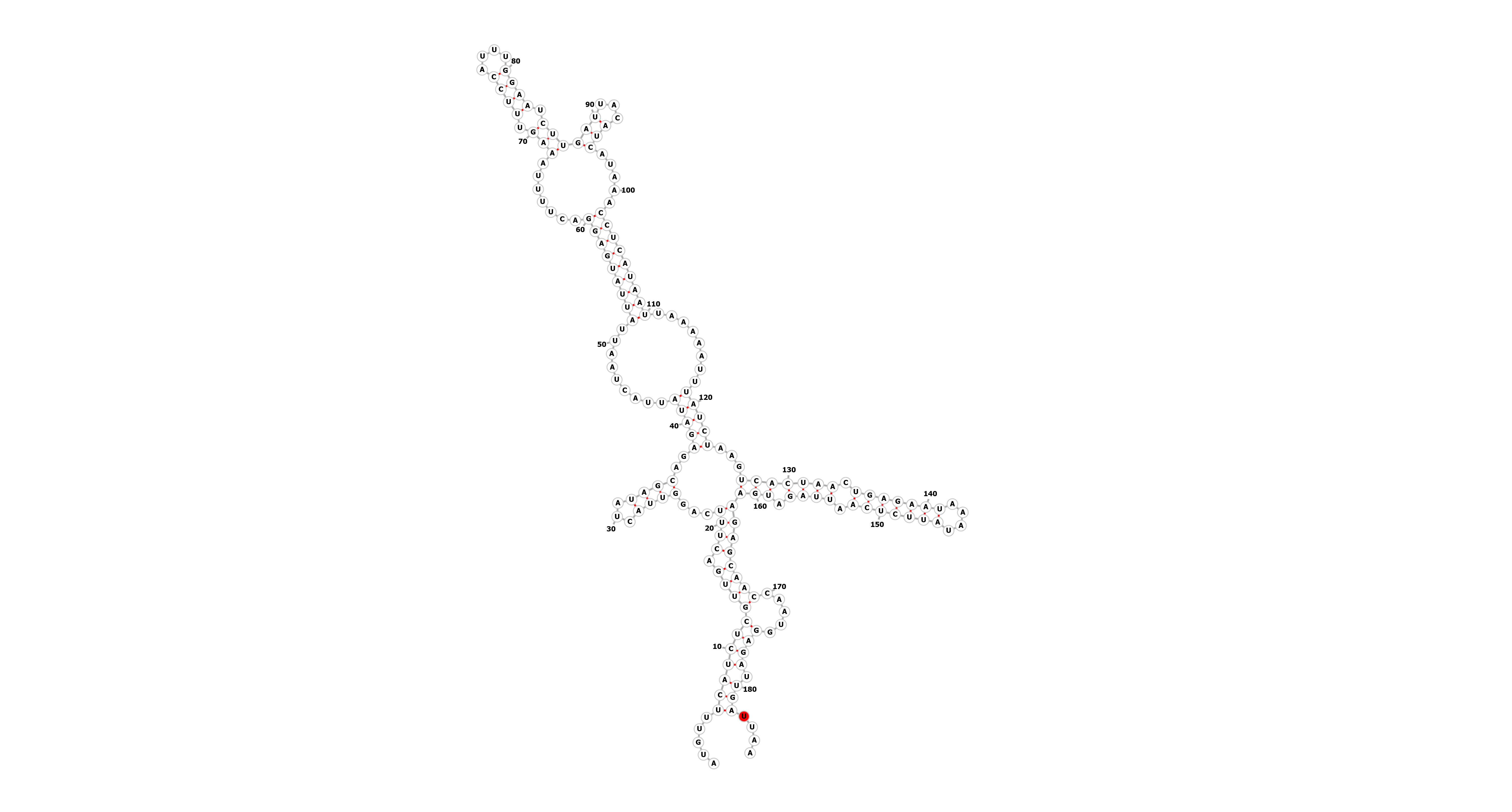


**Figure S8f.** Orf6 RNA secondary structure. Red circles represent top synonymous NT mutations.


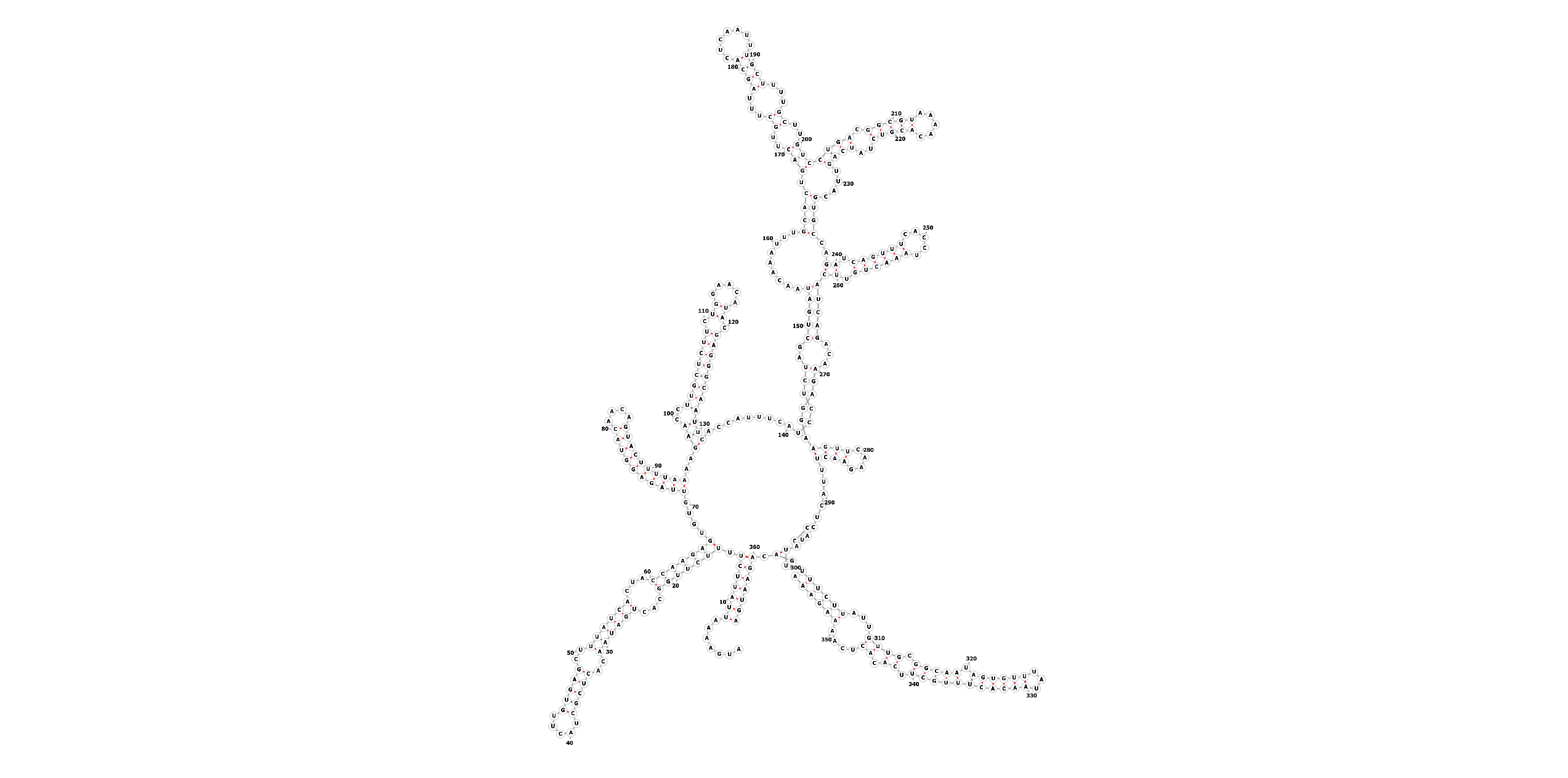


**Figure S8g.** Orf7a RNA secondary structure.


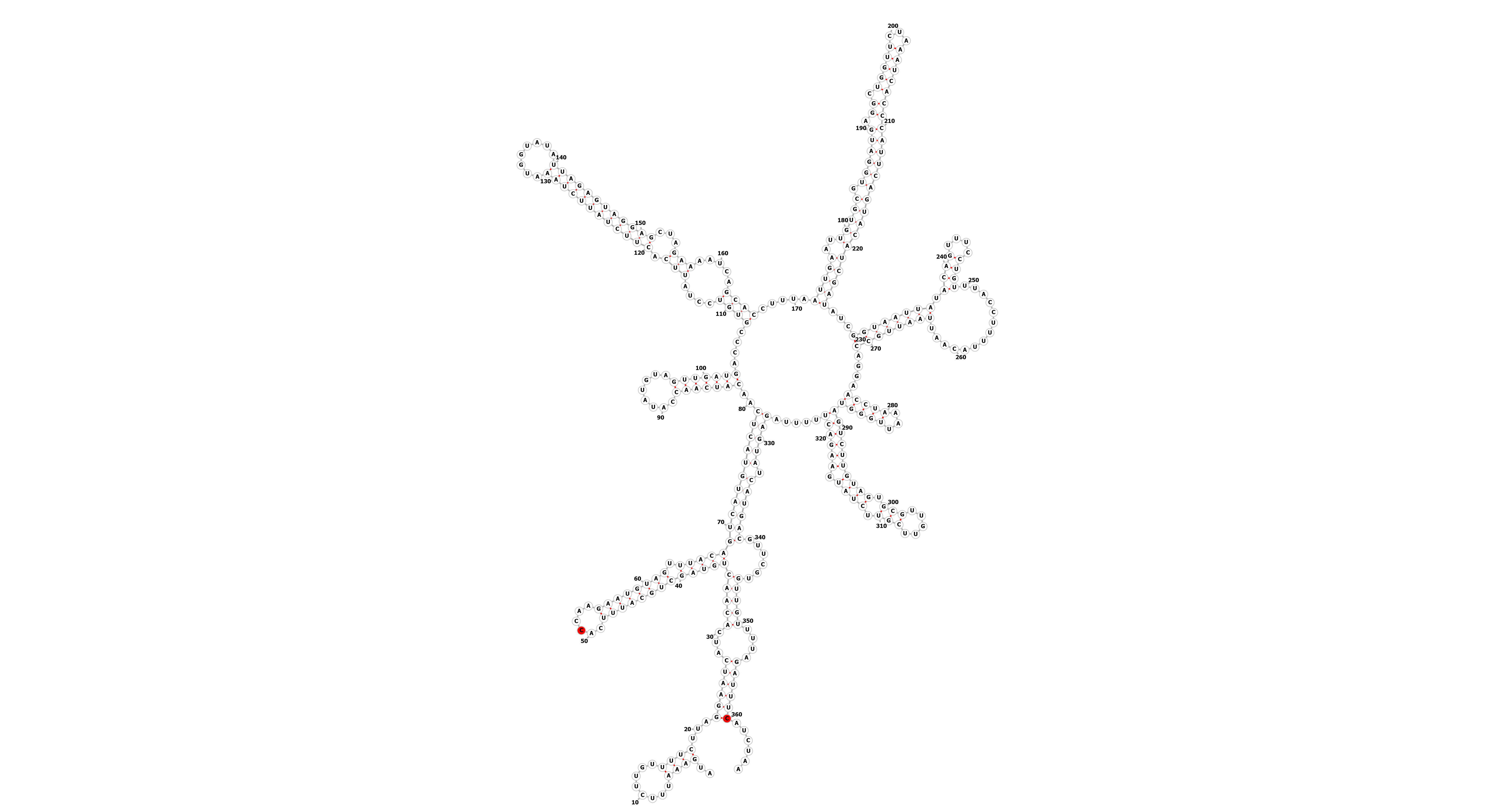


**Figure S8h.** Orf8 RNA secondary structure. Red circles represent top synonymous NT mutations.


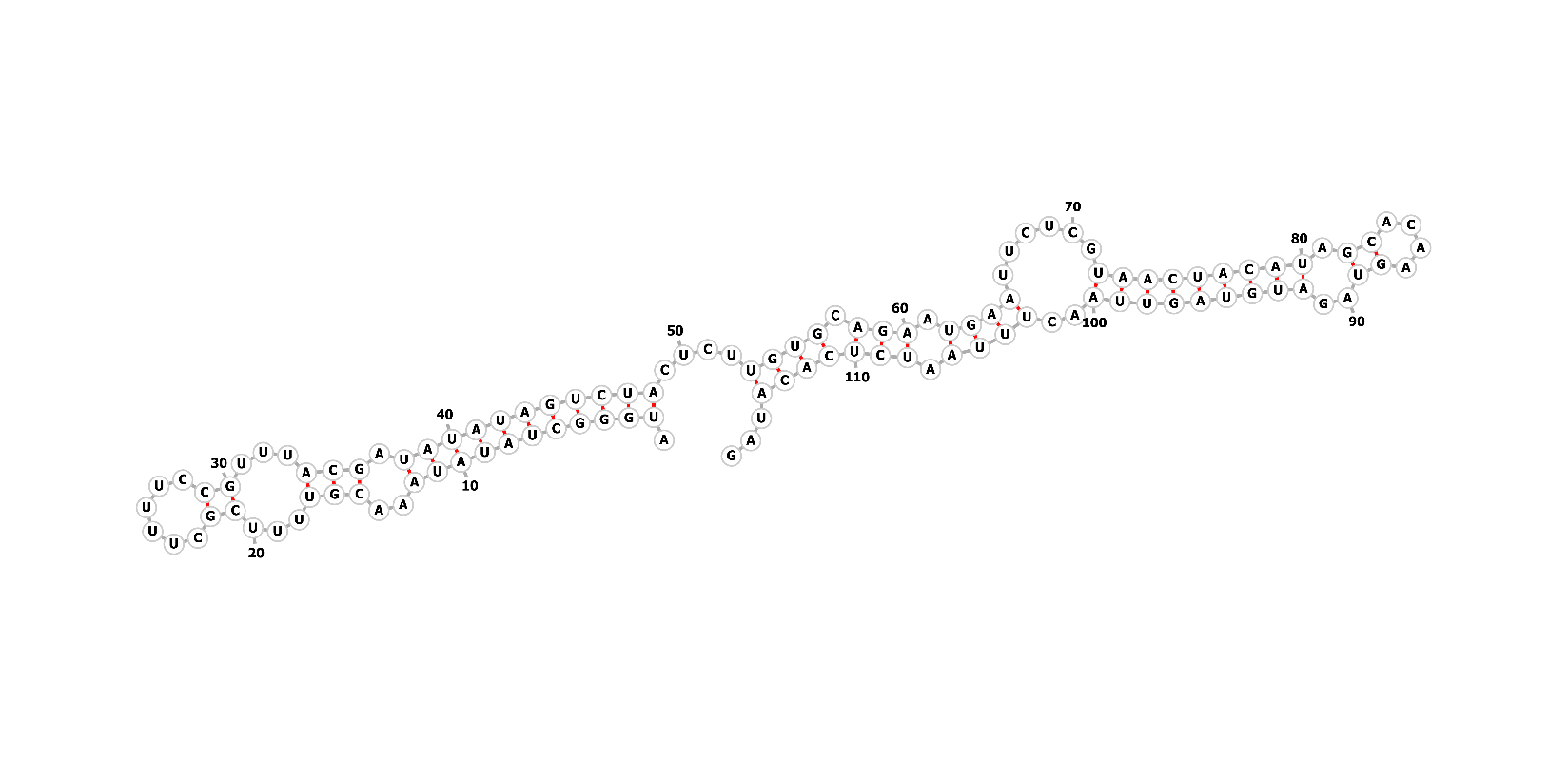


**Figure S8i.** Orf10 RNA secondary structure.


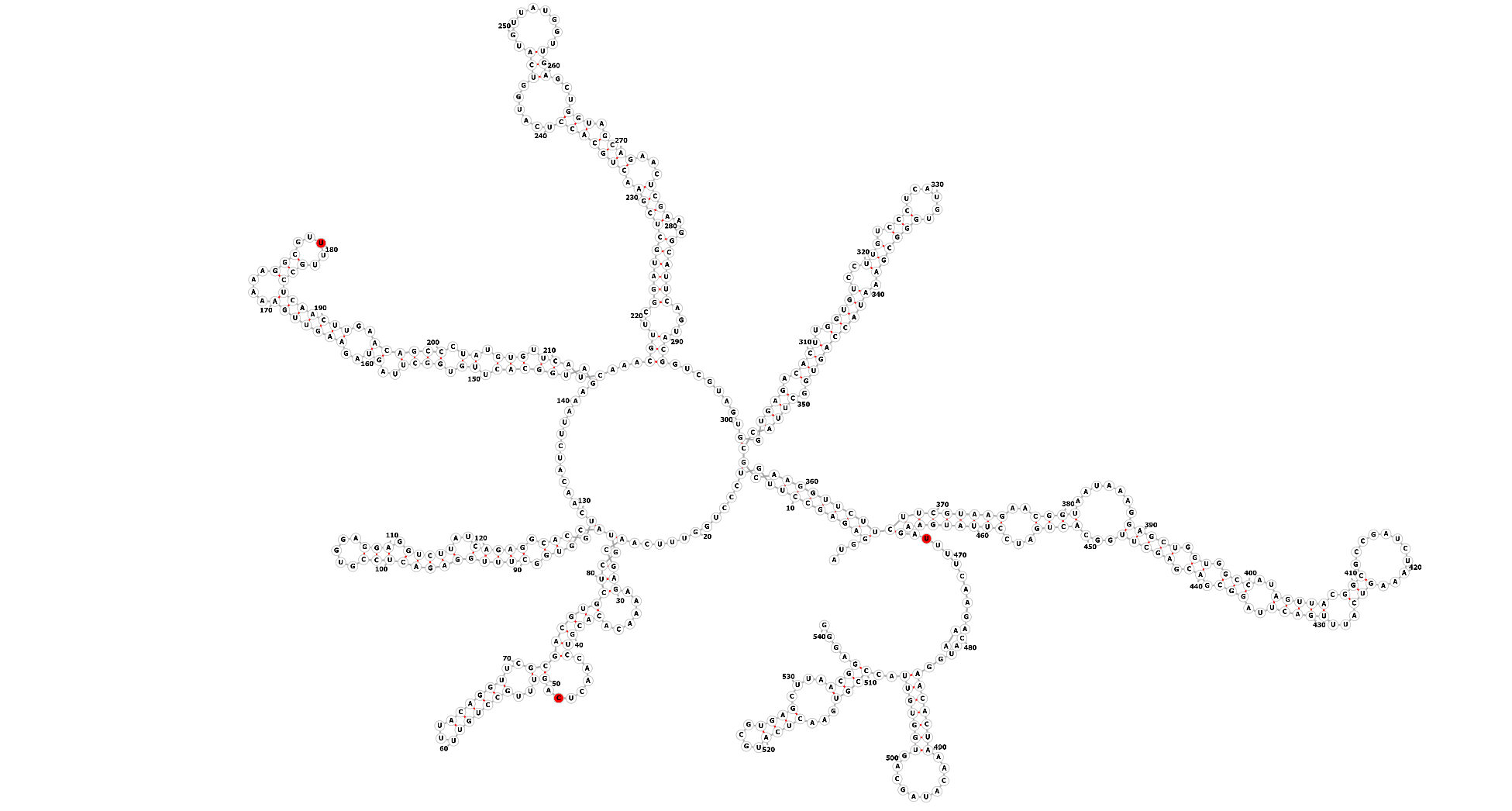


**Figure S8j.** NSP1 RNA secondary structure. Red circles represent top synonymous NT mutations.


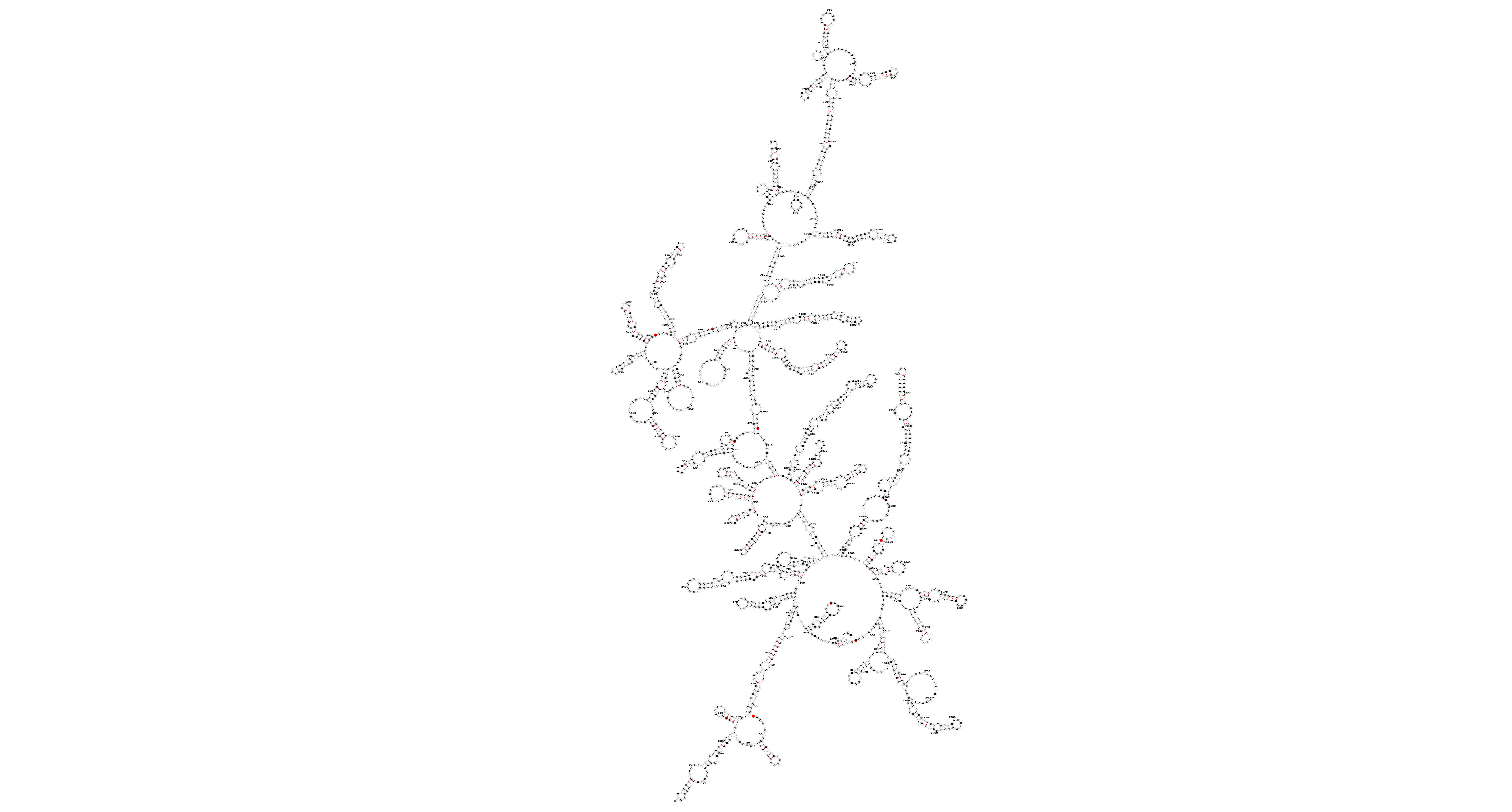


**Figure S8k.** NSP2 RNA secondary structure. Red circles represent top synonymous NT mutations.


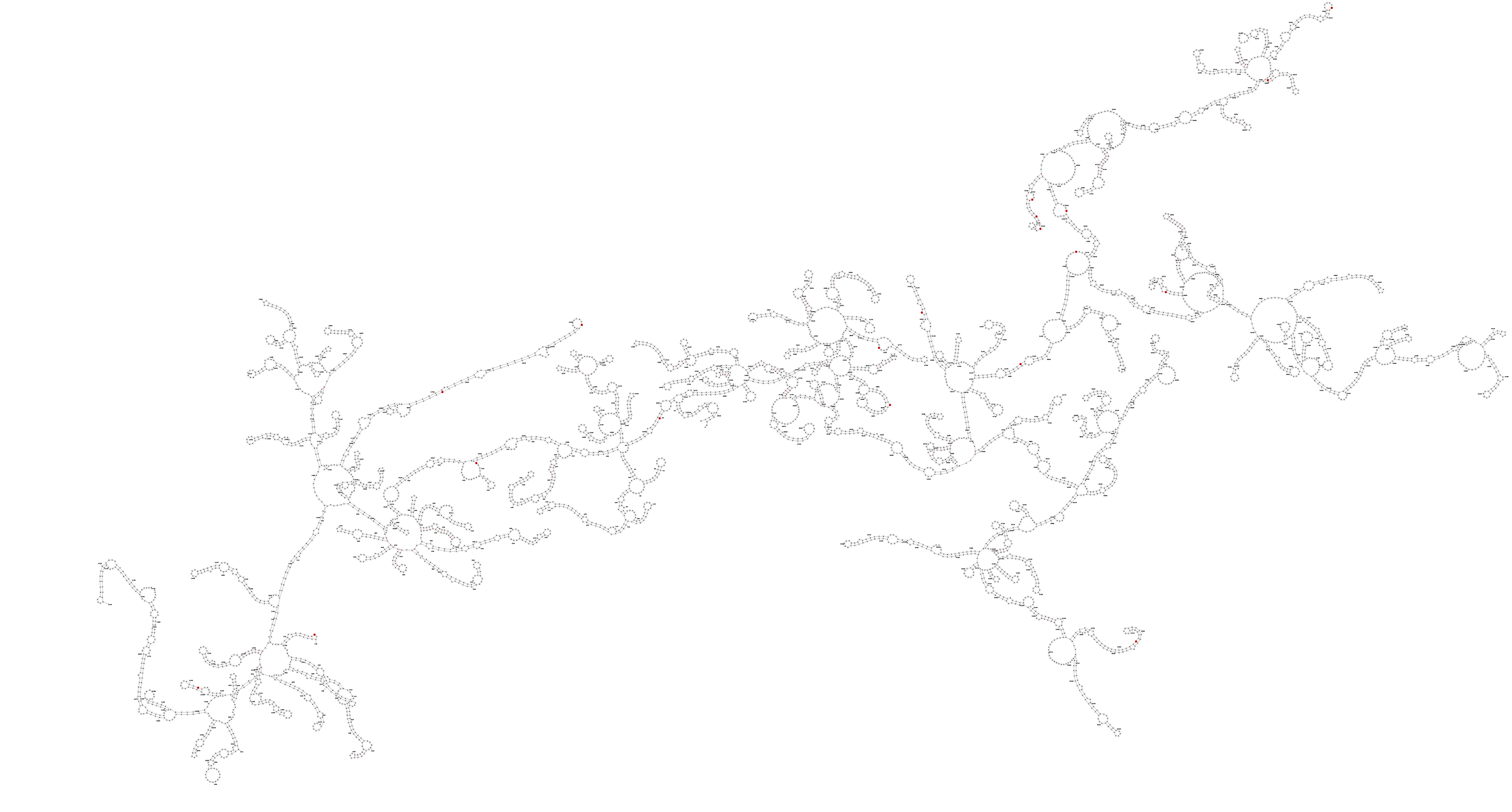


**Figure S8l.** NSP3 RNA secondary structure. Red circles represent top synonymous NT mutations.


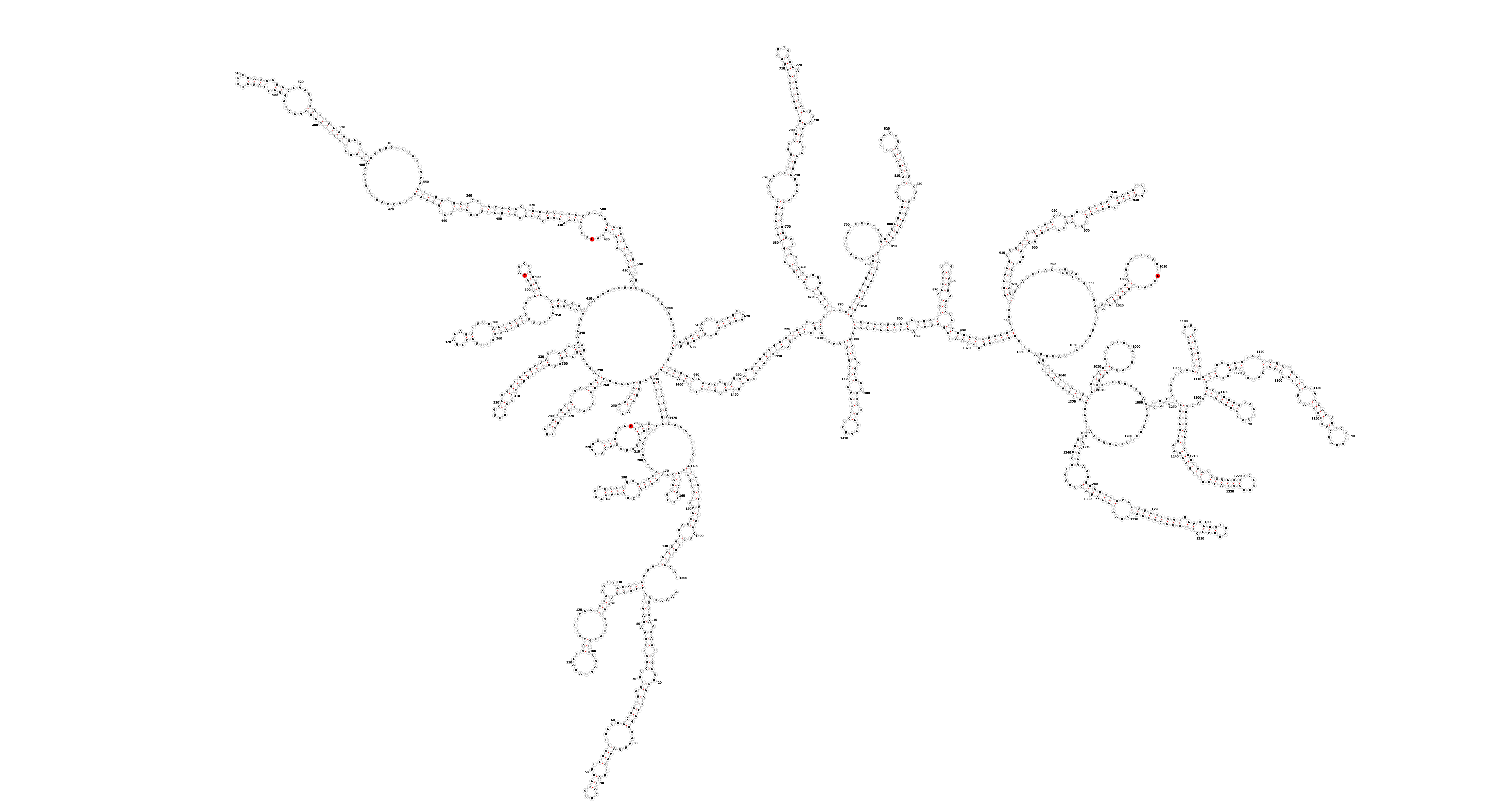


**Figure S8m.** NSP4 RNA secondary structure. Red circles represent top synonymous NT mutations.


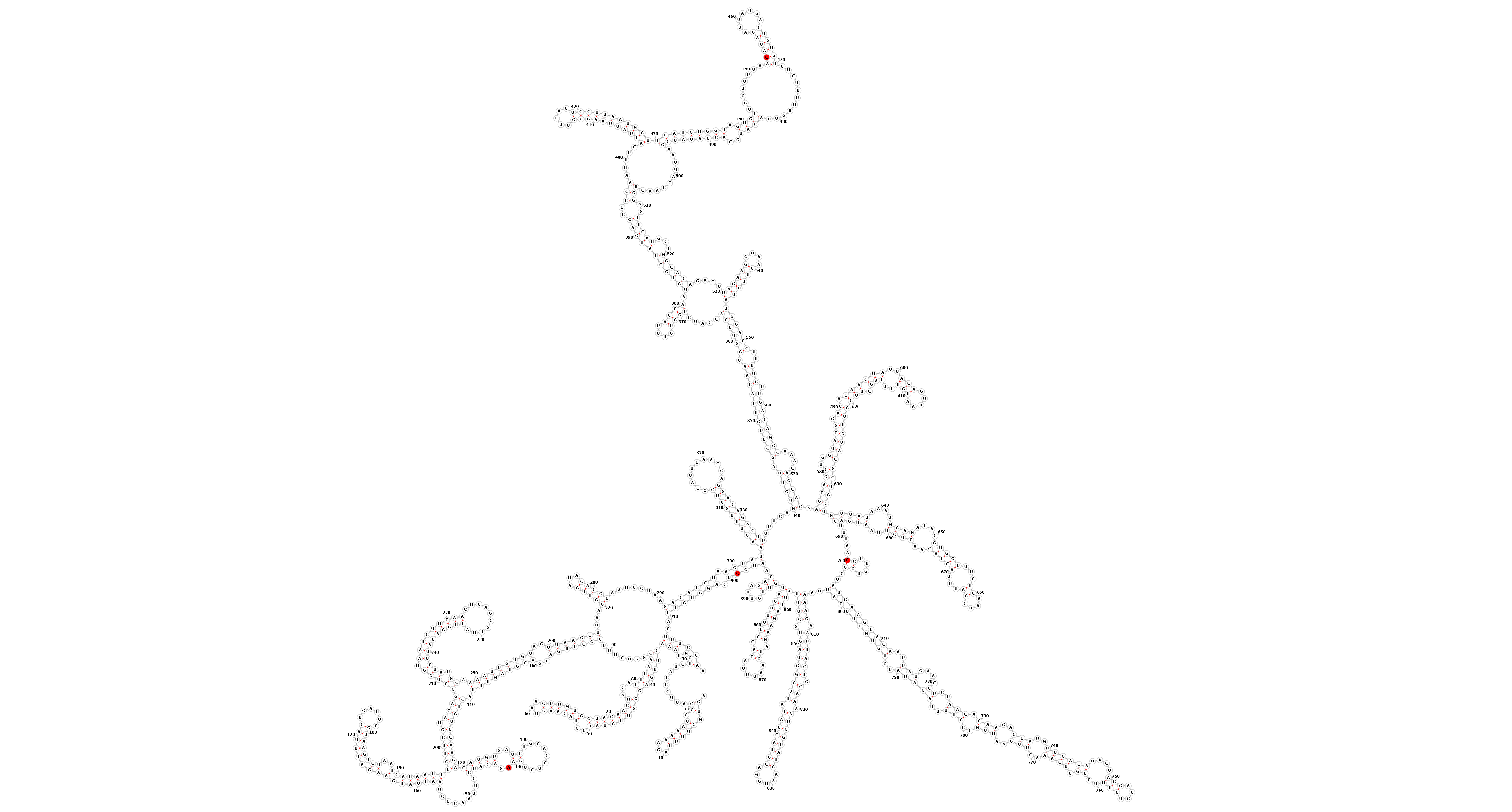


**Figure S8n.** NSP5 RNA secondary structure. Red circles represent top synonymous NT mutations.


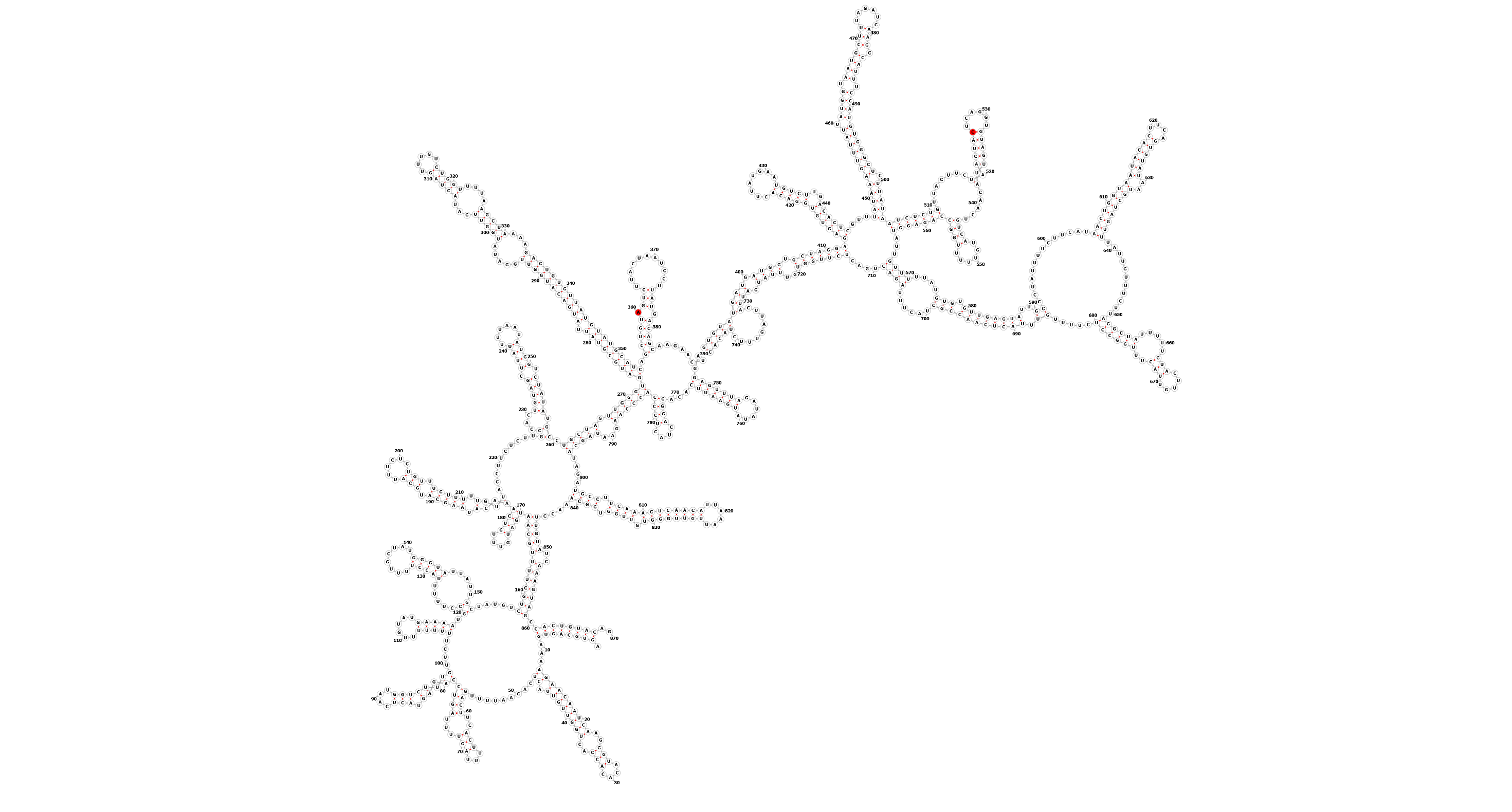


**Figure S8o.** NSP6 RNA secondary structure. Red circles represent top synonymous NT mutations.


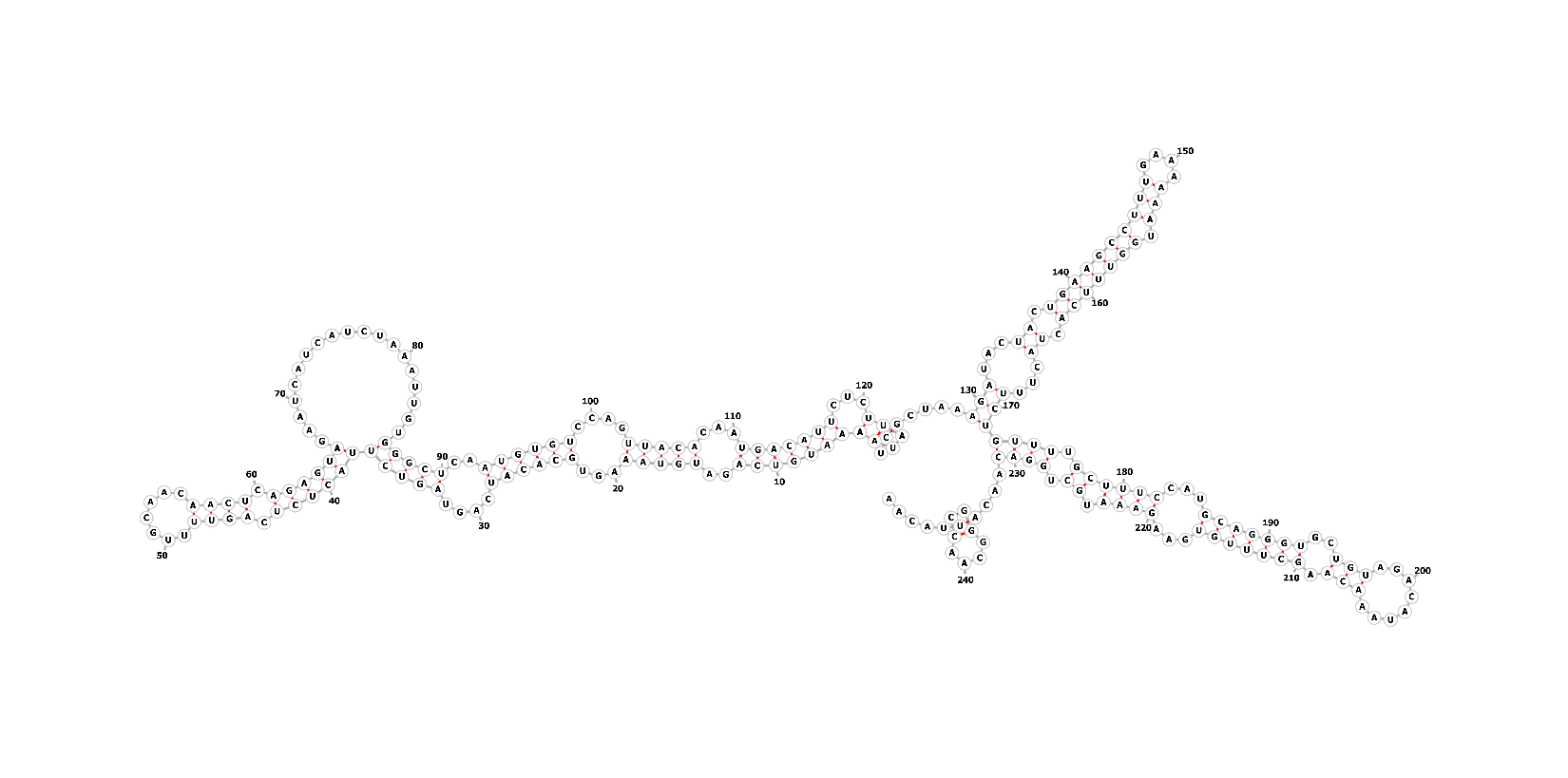


**Figure S8p.** NSP7 RNA secondary structure.


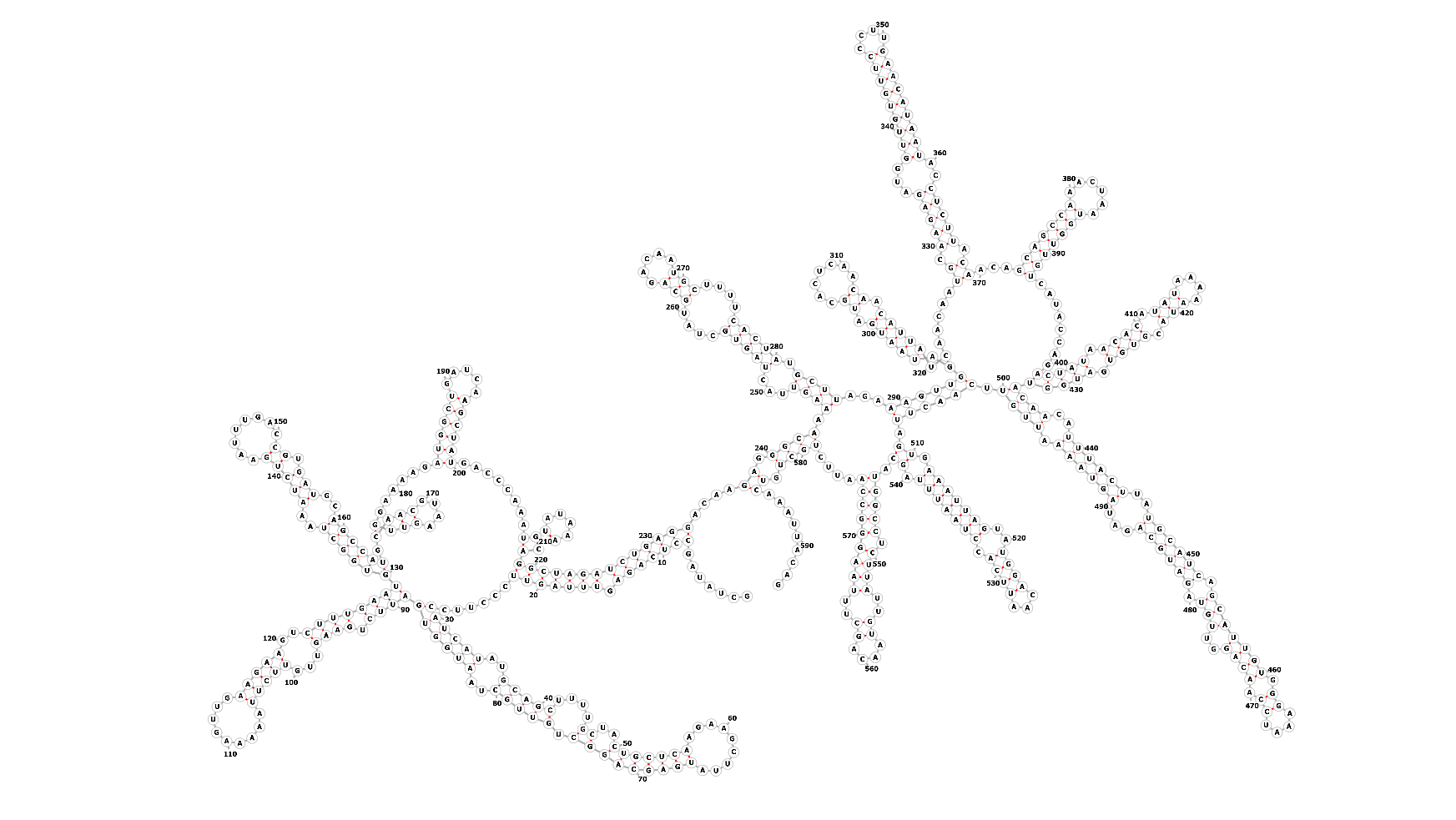


**Figure S8q.** NSP8 RNA secondary structure.


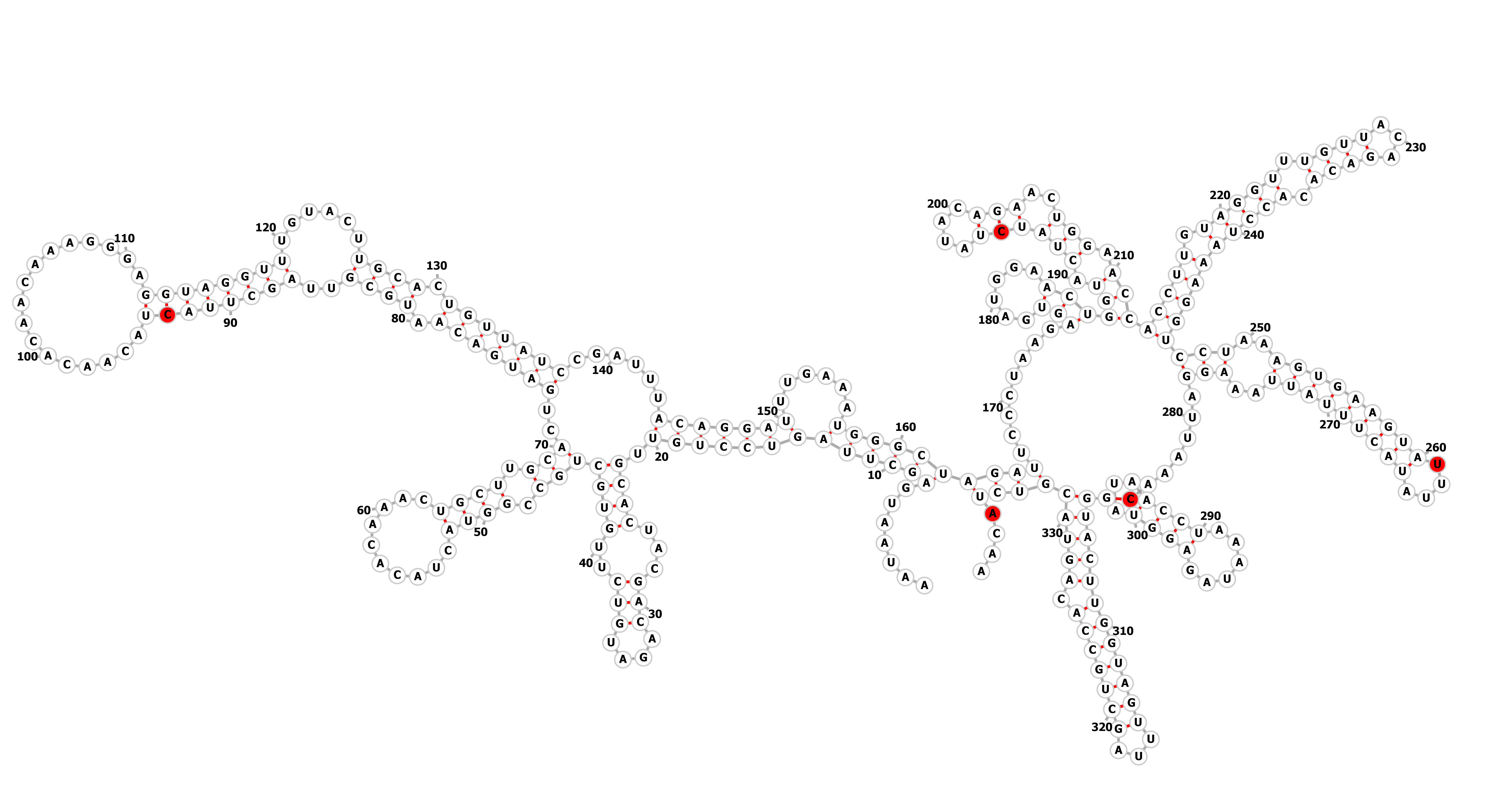


**Figure S8r.** NSP9 RNA secondary structure. Red circles represent top synonymous NT mutations.


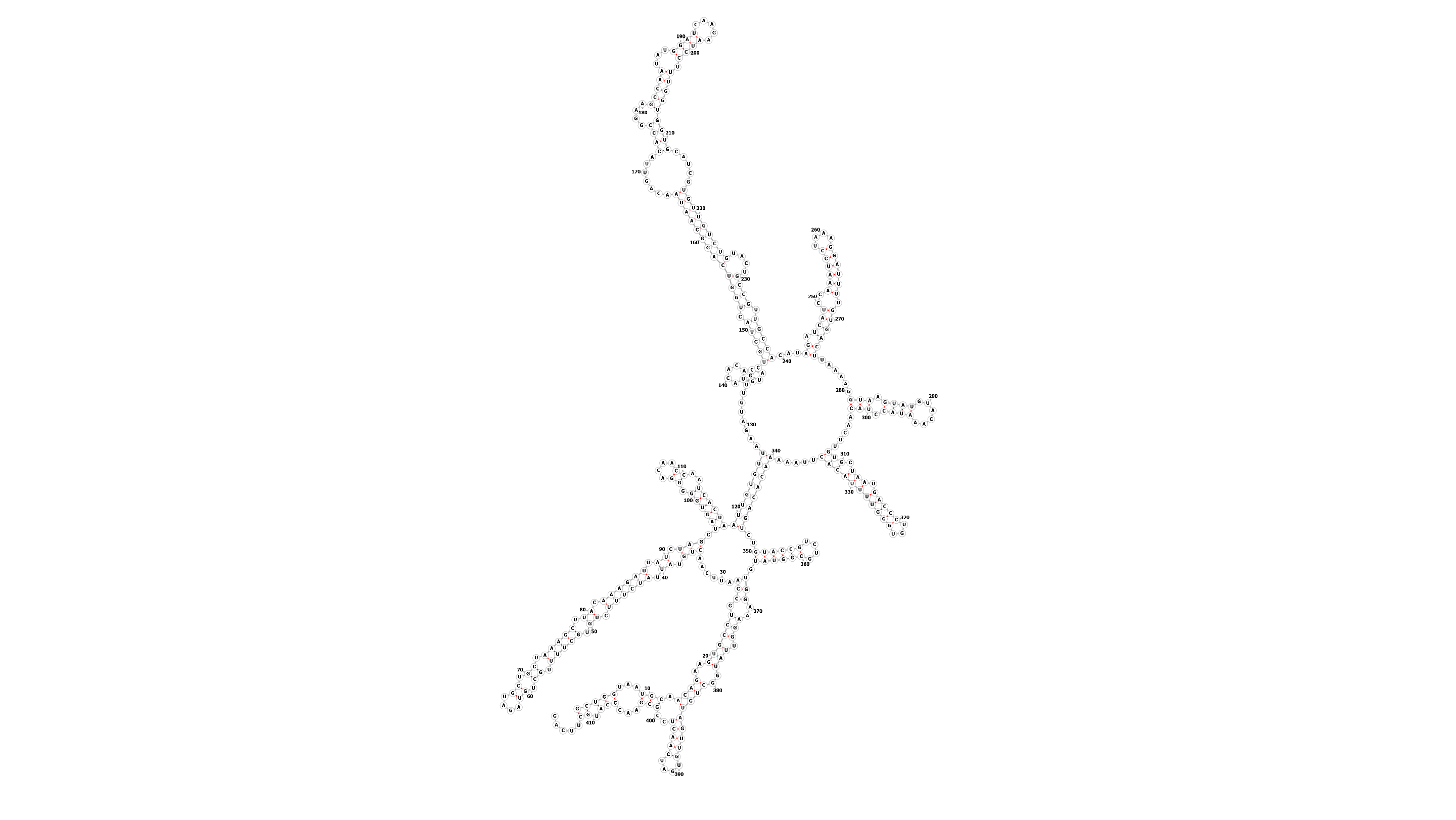


**Figure S8s.** NSP10 RNA secondary structure.


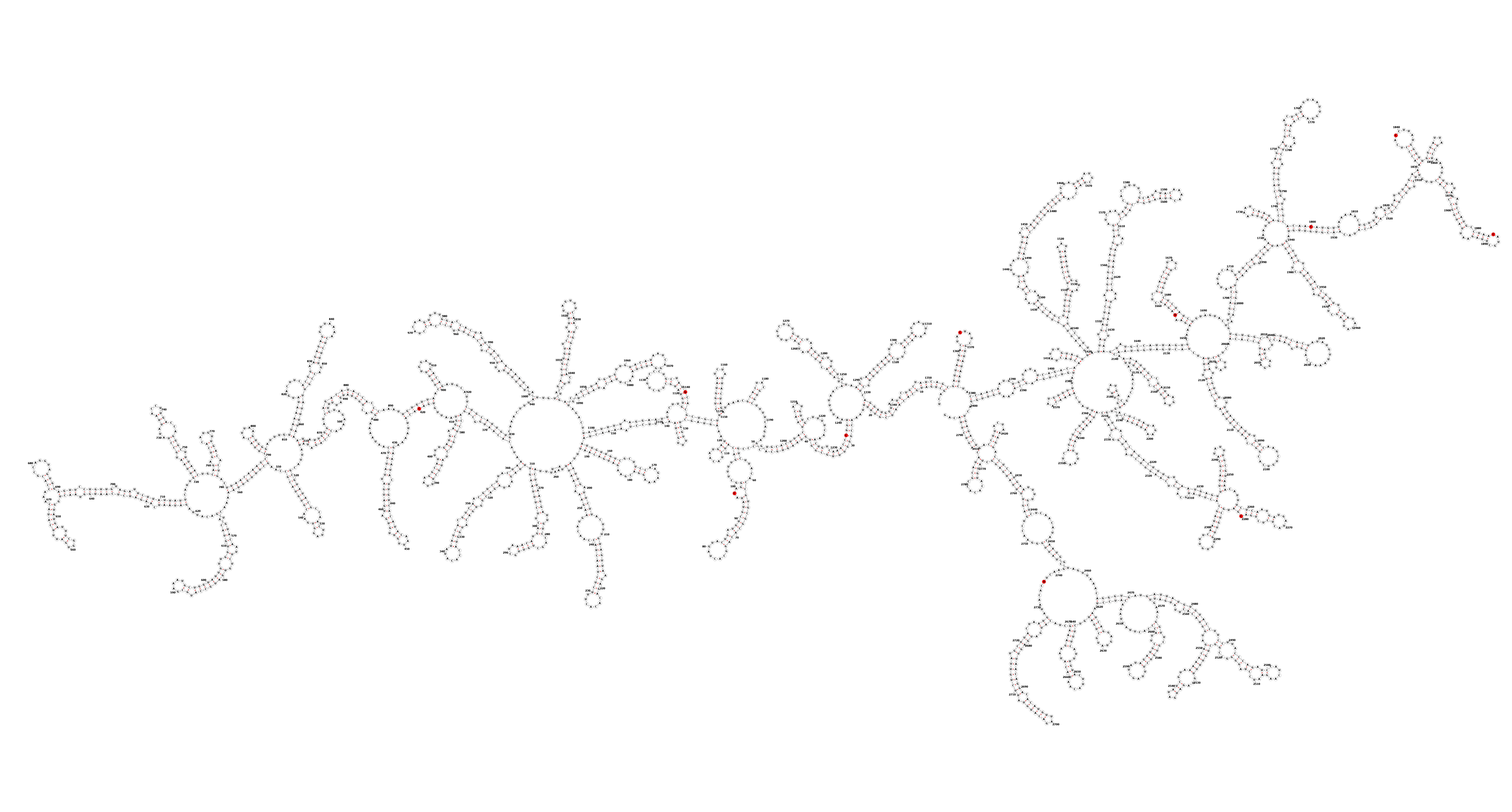


**Figure S8t.** NSP11 RNA secondary structure. Red circles represent top synonymous NT mutations.

**
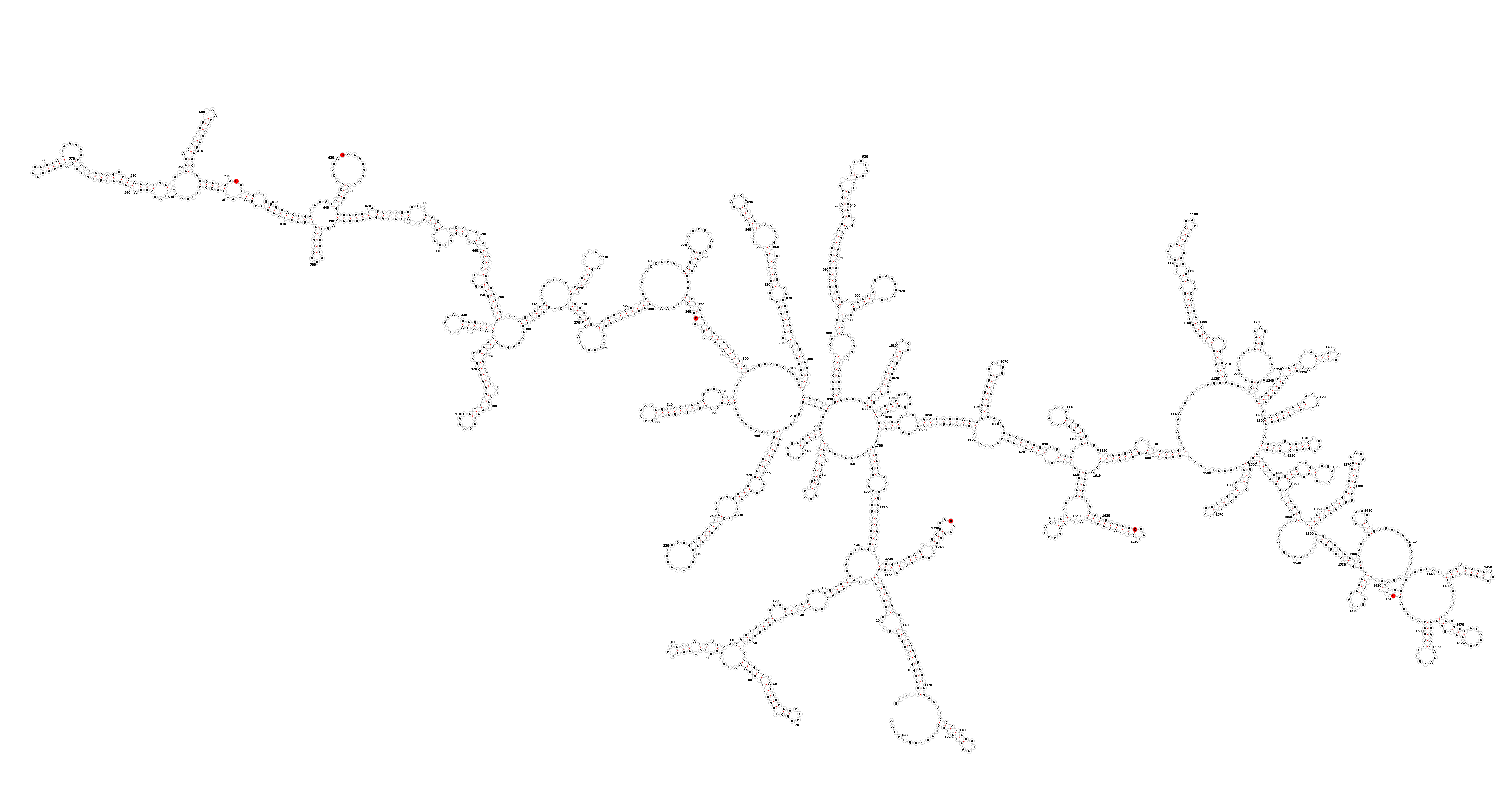
**

**Figure S8u.** NSP12 RNA secondary structure. Red circles represent top synonymous NT mutations.

**
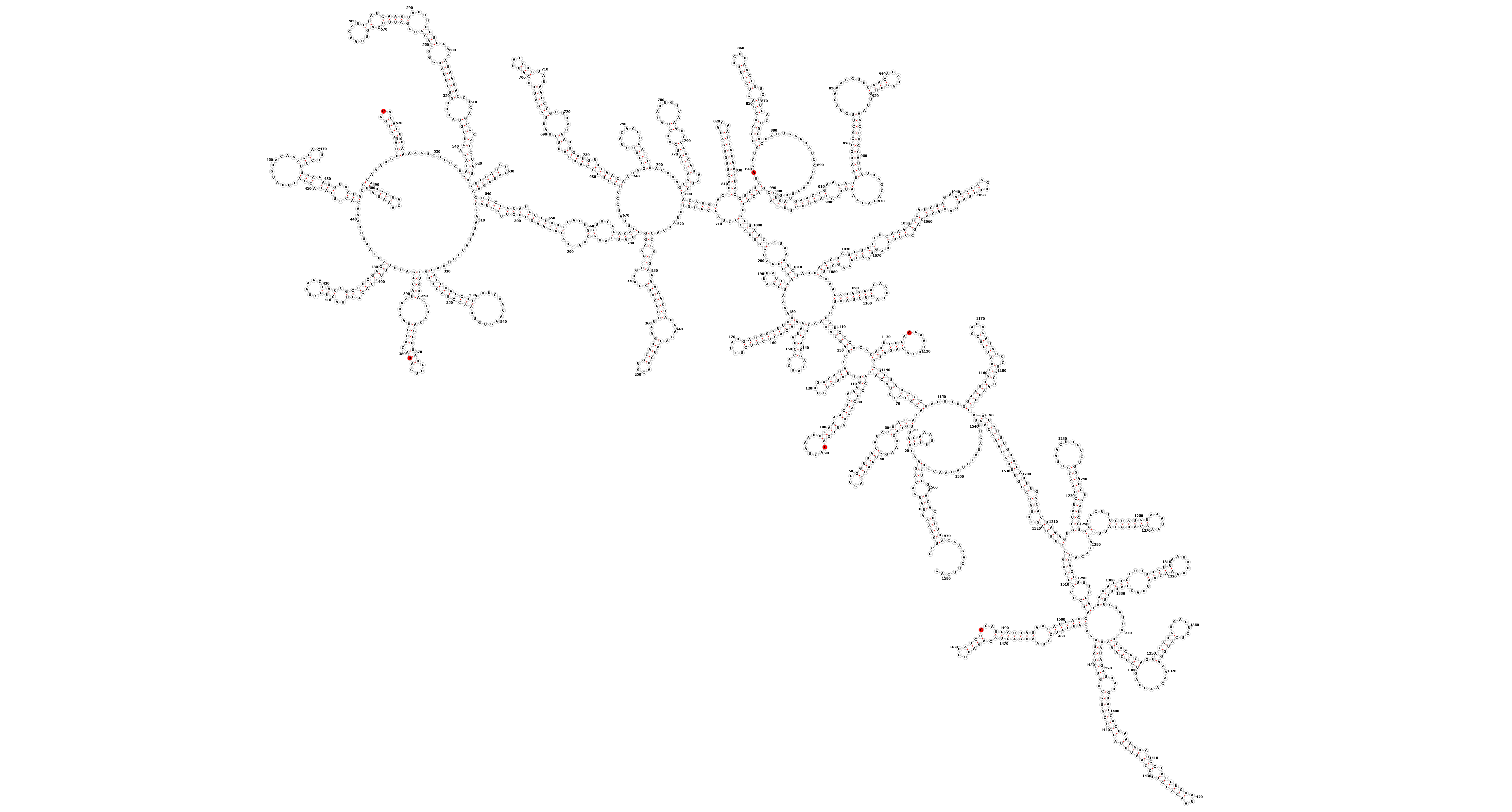
**

**Figure S8v.** NSP13 RNA secondary structure. Red circles represent top synonymous NT mutations.


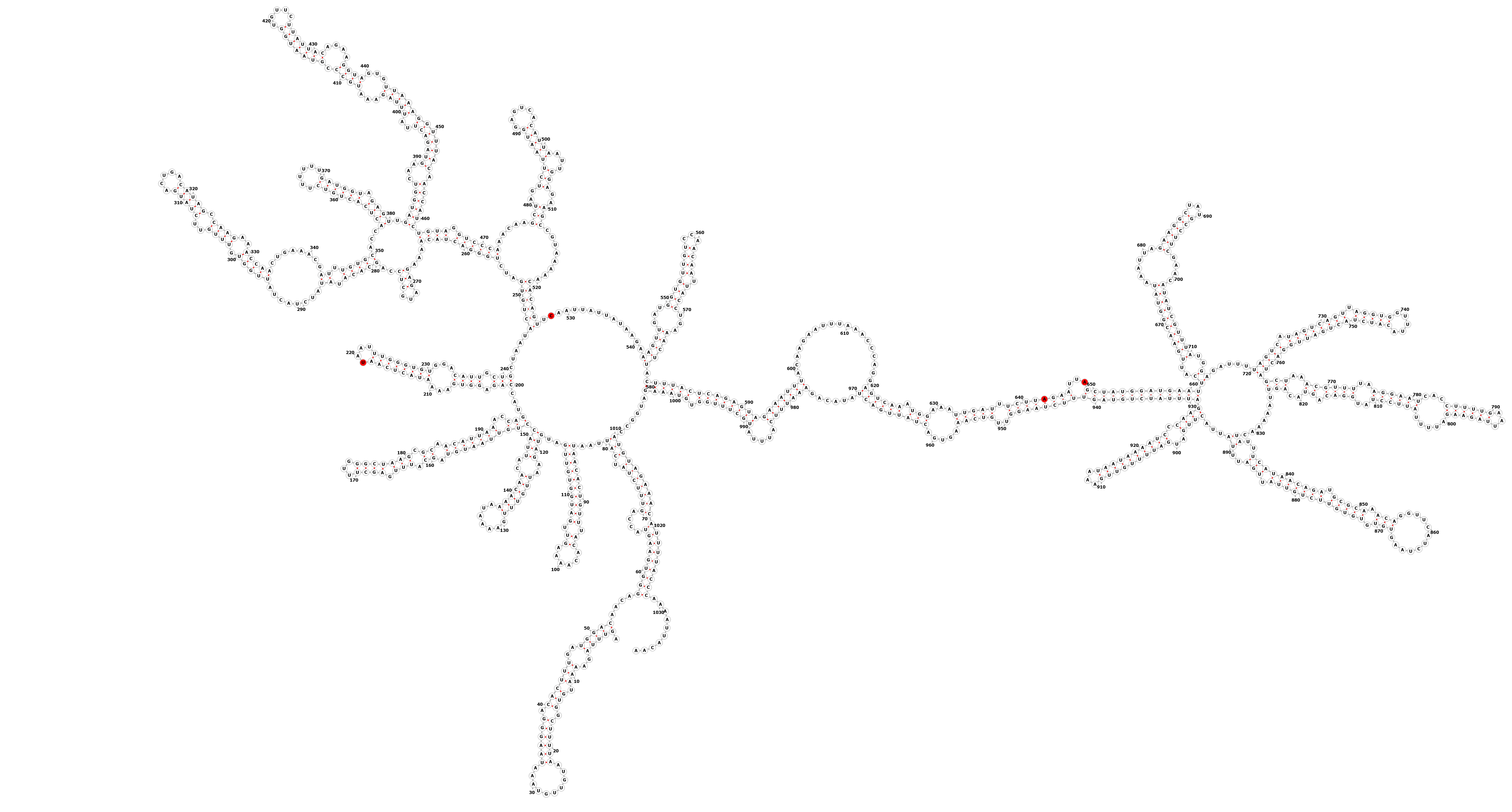


**Figure S8w.** NSP14 RNA secondary structure. Red circles represent top synonymous NT mutations.

**
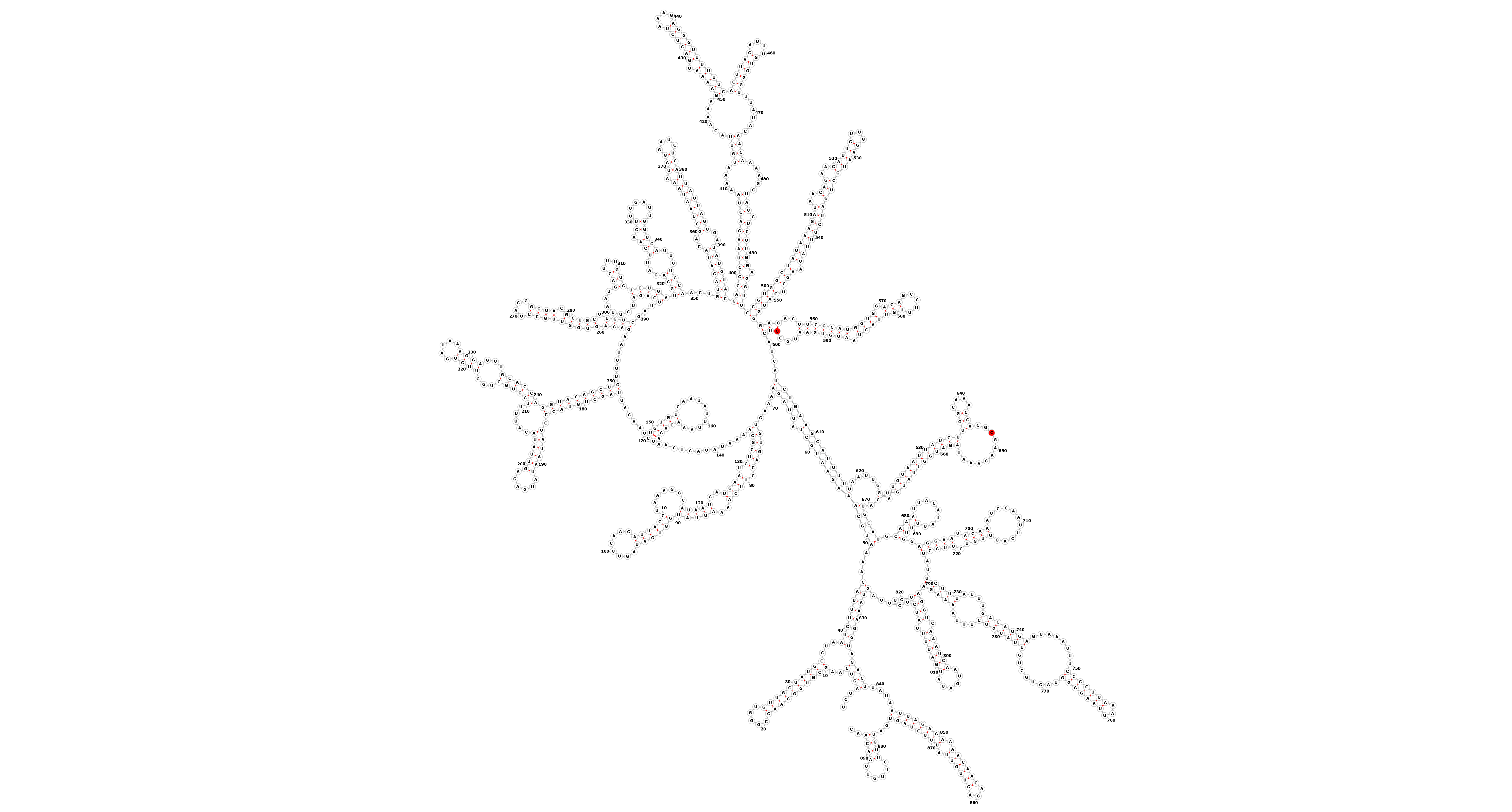
**

**Figure S8x.** NSP15 RNA secondary structure. Red circles represent top synonymous NT mutations.


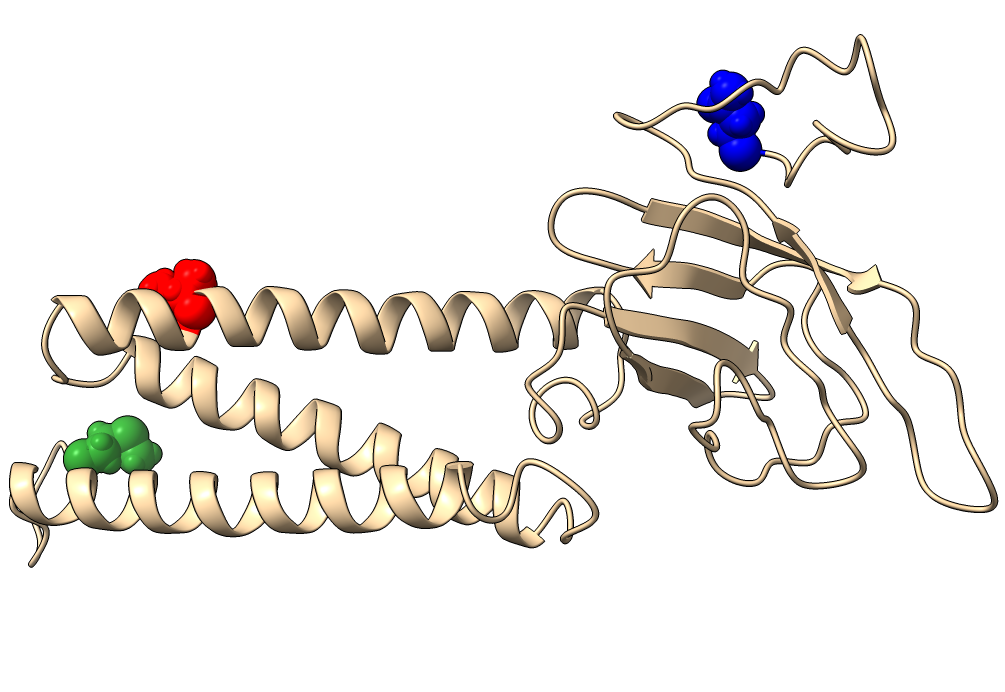


**Figure S9a.** M protein structure (PDB ID: QHD43419**)**. N- and C-termini are represented via green and blue spheres. Top mutations are represented via red spheres.


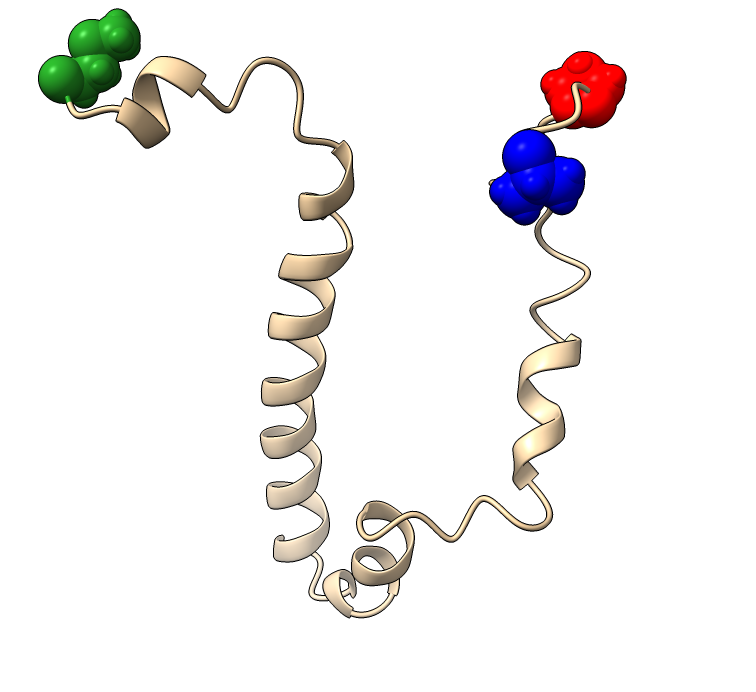


**Figure S9b.** E protein structure (**PDB ID:** 5X29**)**. N- and C-termini are represented via green and blue spheres. Top mutations are represented via red spheres.


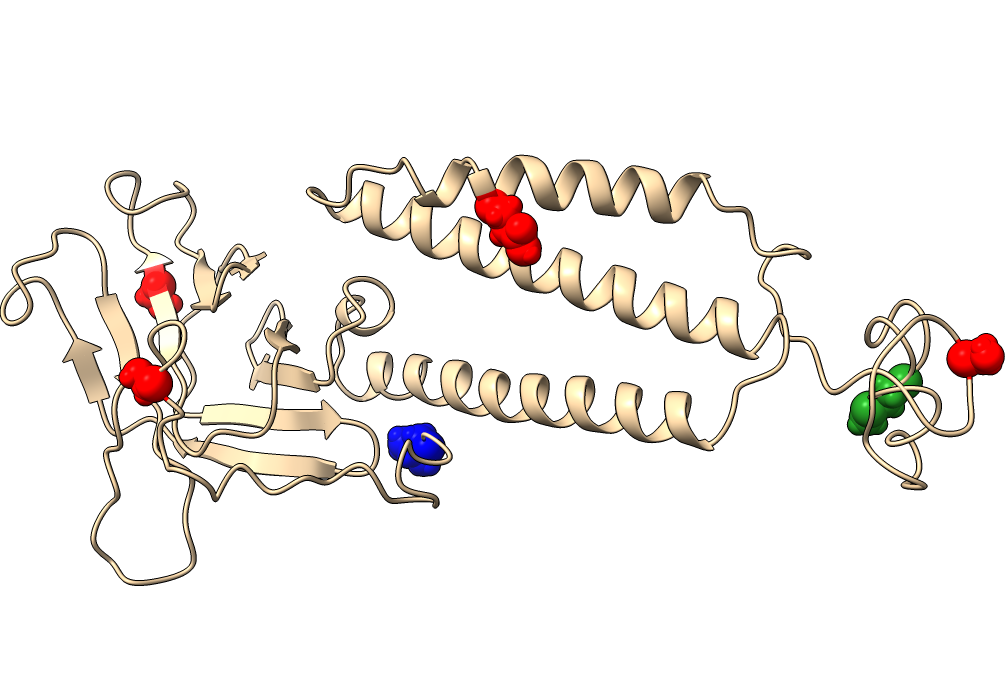


**Figure S9c.** Orf3a protein structure (**PDB ID:** 7KJR). N- and C-termini are represented via green and blue spheres. Top mutations are represented via red spheres.


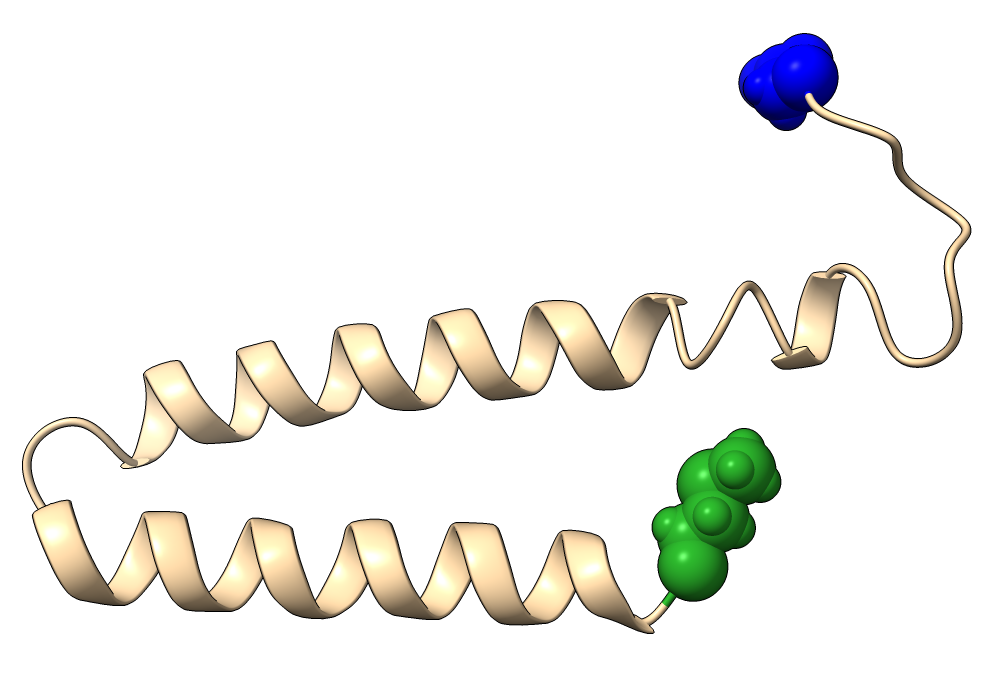


**Figure S9d.** Orf6 protein structure (**PDB ID:** QHD43420). N- and C-termini are represented via green and blue spheres.

**
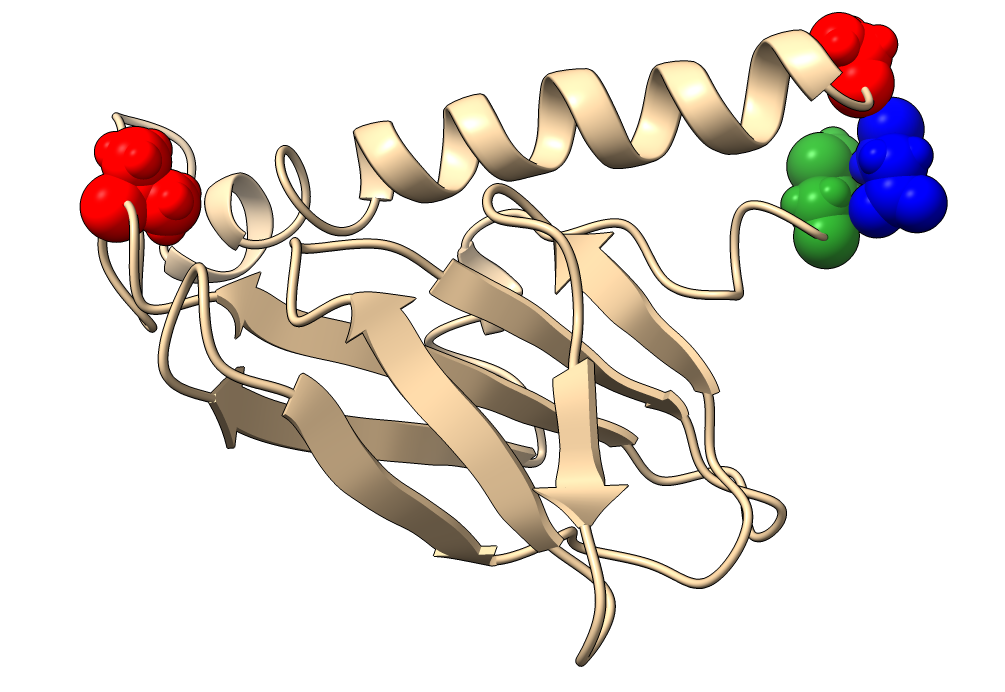
**

**Figure S9e.** Orf7a protein structure (**PDB ID:** QHD43421). N- and C-termini are represented via green and blue spheres. Top mutations are represented via red spheres.


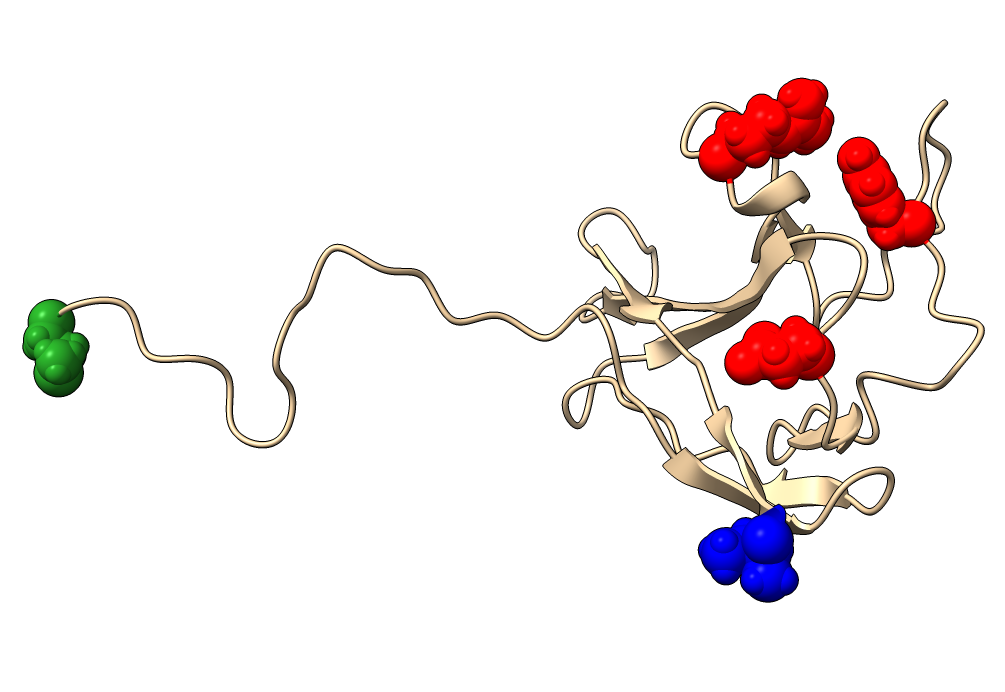


**Figure S9f.** Orf8 protein structure (**PDB ID:** 7JX6). N- and C-termini are represented via green and blue spheres. Top mutations are represented via red spheres.


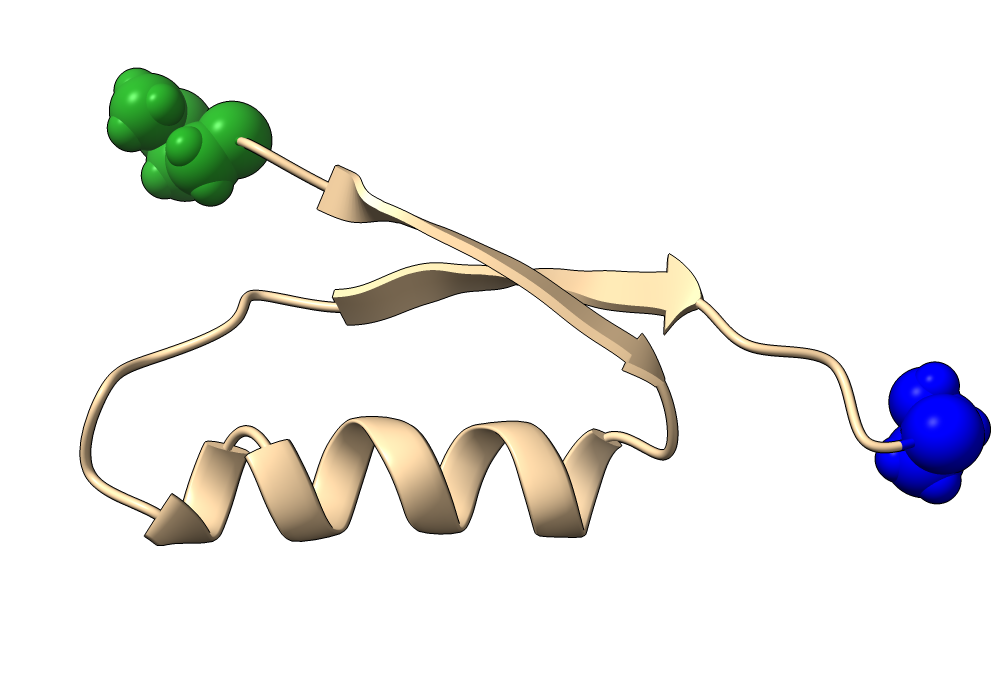


**Figure S9g.** Orf10 protein structure (**PDB ID:** QHI42199). N- and C-termini are represented via green and blue spheres.

**Figure S9h.** NSP1 protein structure (**PDB ID:** QHD43415_1). N- and C-termini are represented via green and blue spheres.

**Figure S9i.** NSP2 protein structure (**PDB ID:** QHD43415_2). N- and C-termini are represented via green and blue spheres. Top mutations are represented via red spheres.

**Figure S9j.** NSP3 protein structure (**PDB ID:** QHD43415_3). N- and C-termini are represented via green and blue spheres. Top mutations are represented via red spheres.

**Figure S9k.** NSP4 protein structure (**PDB ID:** QHD43415_4). N- and C-termini are represented via green and blue spheres. Top mutations are represented via red spheres.

**Figure S9l.** NSP6 protein structure (**PDB ID:** QHD43415_6). N- and C-termini are represented via green and blue spheres. Top mutations are represented via red spheres.

**Figure S9m.** NSP7 protein structure (**PDB ID:** 6M5I). N- and C-termini are represented via green and blue spheres

**Figure S9n.** NSP8 protein structure (**PDB ID:** 7CYQ). N- and C-termini are represented via green and blue spheres.

**Figure S9o.** NSP9 protein structure (**PDB ID:** 7KRI). N- and C-termini are represented via green and blue spheres.

**Figure S9p.** NSP10 protein structure (**PDB ID:** 7N0D). N- and C-termini are represented via green and blue spheres.

**Figure S9q.** NSP12 protein structure (**PDB ID:** 6XEZ). N- and C-termini are represented via green and blue spheres. Top mutations are represented via red spheres.

**Figure S9r.** NSP13 protein structure (**PDB ID:** 7EGQ). N- and C-termini are represented via green and blue spheres. Top mutations are represented via red spheres.

**Figure S9s.** NSP14 protein structure (**PDB ID:** 6WXC). N- and C-termini are represented via green and blue spheres.

**Figure S9t.** NSP15 protein structure (**PDB ID:** 716R). N- and C-termini are represented via green and blue spheres.

| **NSP5** | | | |
| --- | --- | --- | --- |
| **Nirmatrelvir** | | **Ensitrelvir** | |
| **Lufotrelvir** | | **GC376** | |
| **NSP11** | | | |
| **Dasabuvir** | **Favipiravir Triphosphate** | | **Galidesivir Triphosphate** |
| **Ribavirin Triphosphate** | **Sofosbuvir Triphosphate** | | **Remdesivir Triphosphate** |

**Figure S10.** 2D chemical structures of the FDA-approved antiviral compounds docked to the NSP5 and NSP11 wild-type and mutant homology model structures.
